# Initiator tRNA lacking 1-methyladenosine is targeted by the rapid tRNA decay pathway in evolutionarily distant yeast species

**DOI:** 10.1371/journal.pgen.1010215

**Published:** 2022-07-28

**Authors:** Monika Tasak, Eric M. Phizicky

**Affiliations:** Department of Biochemistry and Biophysics, Center for RNA Biology, University of Rochester School of Medicine, Rochester, New York, United States of America; Eunice Kennedy Shriver National Institute of Child Health and Human Development: National Institute of Child Health and Human Development, UNITED STATES

## Abstract

All tRNAs have numerous modifications, lack of which often results in growth defects in the budding yeast *Saccharomyces cerevisiae* and neurological or other disorders in humans. In *S*. *cerevisiae*, lack of tRNA body modifications can lead to impaired tRNA stability and decay of a subset of the hypomodified tRNAs. Mutants lacking 7-methylguanosine at G_46_ (m^7^G_46_), N_2_,N_2_-dimethylguanosine (m^2,2^G_26_), or 4-acetylcytidine (ac^4^C_12_), in combination with other body modification mutants, target certain mature hypomodified tRNAs to the rapid tRNA decay (RTD) pathway, catalyzed by 5’-3’ exonucleases Xrn1 and Rat1, and regulated by Met22. The RTD pathway is conserved in the phylogenetically distant fission yeast *Schizosaccharomyces pombe* for mutants lacking m^7^G_46_. In contrast, *S*. *cerevisiae trm6/gcd10* mutants with reduced 1-methyladenosine (m^1^A_58_) specifically target pre-tRNA_i_^Met(CAU)^ to the nuclear surveillance pathway for 3’-5’ exonucleolytic decay by the TRAMP complex and nuclear exosome. We show here that the RTD pathway has an unexpected major role in the biology of m^1^A_58_ and tRNA_i_^Met(CAU)^ in both *S*. *pombe* and *S*. *cerevisiae*. We find that *S*. *pombe trm6Δ* mutants lacking m^1^A_58_ are temperature sensitive due to decay of tRNA_i_^Met(CAU)^ by the RTD pathway. Thus, *trm6Δ* mutants had reduced levels of tRNA_i_^Met(CAU)^ and not of eight other tested tRNAs, overexpression of tRNA_i_^Met(CAU)^ restored growth, and spontaneous suppressors that restored tRNA_i_^Met(CAU)^ levels had mutations in *dhp1/RAT1* or *tol1/MET22*. In addition, deletion of *cid14*/*TRF4* in the nuclear surveillance pathway did not restore growth. Furthermore, re-examination of *S*. *cerevisiae trm6* mutants revealed a major role of the RTD pathway in maintaining tRNA_i_^Met(CAU)^ levels, in addition to the known role of the nuclear surveillance pathway. These findings provide evidence for the importance of m^1^A_58_ in the biology of tRNA_i_^Met(CAU)^ throughout eukaryotes, and fuel speculation that the RTD pathway has a major role in quality control of body modification mutants throughout fungi and other eukaryotes.

## Author summary

tRNA modifications are highly conserved, and their lack frequently results in growth defects in the yeast *Saccharomyces cerevisiae* and neurological disorders in humans. In *S*. *cerevisiae* lack of 1-methyladenosine at N_58_ (m^1^A_58_) in the tRNA body is lethal due to 3’-5’ decay of pre-tRNA_i_^Met^ by the nuclear surveillance pathway. By contrast, lack of any of three other body modifications causes growth defects due to 5’-3’ decay of specific hypomodified tRNAs by the rapid tRNA decay (RTD) pathway. Despite their importance, little is known about either tRNA_i_^Met^ quality control or tRNA decay pathways in eukaryotes other than *S*. *cerevisiae*.

Here we show an unexpected role of the RTD pathway in quality control of tRNA_i_^Met^ lacking m^1^A_58_ in the phylogenetically distant yeast species *Schizosaccharomyces pombe* and *S*. *cerevisiae*. We find that *S*. *pombe trm6Δ* mutants, lacking m^1^A_58_, are temperature sensitive due to decay of tRNA_i_^Met(CAU)^ primarily by the RTD pathway. Furthermore, re-investigation of *S*. *cerevisiae trm6* mutants revealed a significant role of the RTD pathway, in addition to the nuclear surveillance pathway, in decay of tRNA_i_^Met(CAU)^. Our results suggest that throughout eukaryotes the RTD pathway has a major role in decay of hypomodified tRNAs and that m^1^A_58_ is crucial to tRNA_i_^Met(CAU)^ biology.

## Introduction

tRNAs are central to the process of translation, a role that is enabled by their extensive and highly conserved post-transcriptional modifications [1–3]. Lack of any of a number of modifications causes growth defects in the budding yeast *Saccharomyces cerevisiae* [3], as well as a number of neurological or mitochondrial disorders in humans [4, 5]. Lack of modifications in and around the anticodon loop (ACL) frequently reduces the efficiency and/or fidelity of mRNA decoding [6–8], disrupts reading-frame maintenance [9, 10], or decreases charging efficiency and/or fidelity [11]. Lack of modifications in the main tRNA body (outside the ACL) often results in altered folding [12] or reduced tRNA stability, leading to degradation of a subset of the hypomodified tRNAs by one of two characterized decay pathways [13–15].

The rapid tRNA decay (RTD) pathway degrades a subset of the tRNA species lacking any of several body modifications. Degradation of tRNAs by the RTD pathway is catalyzed by the 5’-3’ exonucleases Rat1 and Xrn1, and inhibited by a *met22Δ* mutation [16] due to accumulation of the Met22 substrate adenosine 3’,5’ bisphosphate (pAp) [17, 18]. In *S*. *cerevisiae*, lack of m^7^G_46_, m^2,2^G_26_, or ac^4^C_12_ is known to trigger RTD, and is associated with temperature sensitivity, particularly in combination with lack of other tRNA body modifications. Thus, an *S*. *cerevisiae trm8Δ trm4Δ* mutant (lacking m^7^G_46_ and m^5^C), is temperature sensitive due to decay of mature tRNA^Val(AAC)^ by the RTD pathway [14, 16]. Similarly, an *S*. *cerevisiae tan1Δ trm44Δ* mutant (lacking ac^4^C_12_ and Um_44_) and a *trm1Δ trm4Δ* mutant (lacking m^2,2^G_26_ and m^5^C) are each temperature sensitive due to decay of tRNA^Ser(CGA)^ and tRNA^Ser(UGA)^ [16, 19, 20]. Moreover, each of the corresponding *trm8Δ*, *tan1Δ*, and *trm1Δ* single mutants has an RTD signature, as their temperature sensitivity is suppressed by a *met22Δ* mutation, and is associated with decay of one or more hypomodified tRNA substrates [20].

In addition, recent results show that the RTD pathway also acts on a subset of tRNA species lacking m^7^G_46_ in the phylogenetically distant fission yeast *Schizosaccharomyces pombe*. Thus, the temperature sensitivity of *S*. *pombe trm8Δ* mutants is due to decay of tRNA^Tyr(GUA)^ and to some extent tRNA^Pro(AGG)^, and both the decay and the temperature sensitivity are suppressed by mutations in the *RAT1* ortholog *dhp1* [15].

The other major decay pathway that targets tRNAs lacking a body modification in *S*. *cerevisiae* is the nuclear surveillance pathway, which degrades the precursor of initiator tRNA (pre-tRNA_i_^Met(CAU)^) lacking m^1^A_58_ [13, 21]. Degradation by this pathway is catalyzed by Trf4 of the TRAMP complex, which oligoadenylates the 3’ end of pre-tRNA_i_^Met(CAU)^, followed by its 3’-5’ exonucleolytic degradation by Rrp6 and Rrp44 of the nuclear exosome [13, 22–25]. The m^1^A_58_ modification is found on numerous tRNA species in *S*. *cerevisiae*, but tRNA_i_^Met(CAU)^ is uniquely different from other tRNAs due in part to its non-canonical nucleotides at A_20_, A_54_, and A_60_ and an unusual substructure involving these residues and m^1^A_58_ [26], presumably accounting for its unique sensitivity to decay in strains lacking m^1^A_58_ [13]. In addition, the nuclear surveillance pathway is also known to target about 50% of all tRNA transcripts, possibly due to stochastic errors during transcription, mis-folding, or natural competition between decay and processing [27].

Understanding the quality control of tRNA_i_^Met(CAU)^ is crucial because of its central role in translation initiation. In this role, tRNA_i_^Met(CAU)^ is a component of the eukaryotic ternary complex that binds the 40S ribosome subunit to form the 43S pre-initiation complex, which in turn binds capped mRNAs and scans their sequence for an appropriate AUG start codon [28]. Moreover, tRNA_i_^Met(CAU)^ is only involved in translation initiation and has its own dedicated set of factors for its delivery to the 40S subunit [29], whereas all other tRNAs participate only in elongation, and their delivery only involves the elongation factor eEF-1A [30], which does not participate in translation initiation. In addition, tRNA_i_^Met(CAU)^ levels are important in regulating the general amino acid control pathway (integrated stress response in humans) by regulation of translation of the transcription factor Gcn4 (human ATF4), which re-programs gene expression in the cell [31, 32]. Furthermore, in human breast epithelial cells, overexpression of tRNA_i_^Met(CAU)^ causes increased cell proliferation and metabolic activity [33], and in mouse, elevated expression of tRNA_i_^Met(CAU)^ stimulates cell migration and drives melanoma invasion [34].

Despite the importance of tRNA_i_^Met(CAU)^ levels in *S*. *cerevisiae* and humans, it is not clear how tRNA_i_^Met(CAU)^ levels are regulated in eukaryotes. Although there is compelling evidence in *S*. *cerevisiae* that the nuclear surveillance pathway targets pre-tRNA_i_^Met(CAU)^ for decay in *trm6/gcd10* or *trm61/gcd14* mutants with reduced m^1^A_58_, available information in other eukaryotes suggests the possibility of alternative pathways. HeLa cells that are heat shocked at 43°C undergo decay of tRNA_i_^Met(CAU)^ by Xrn1 and Rat1 over several hours, but it is not clear why decay occurred, as there was no obvious change in the modifications or physical stability of the remaining tRNA_i_^Met(CAU)^ [35]. Moreover, tRNA_i_^Met(CAU)^ levels in human cells were upregulated by knockdown of ALKBH1, which has an m^1^A-demethylase activity, and glucose starvation led to increased ALKBH1, linked to reduced tRNA_i_^Met(CAU)^ and reduced translation [36]. Although the mechanisms by which tRNA_i_^Met(CAU)^ levels are regulated are not known, it is clear that there is a link between m^1^A_58_ modification status and tRNA_i_^Met(CAU)^ levels in eukaryotes. Thus, depletion of TRM6 or TRM61 in human cells results in reduced levels of tRNA_i_^Met(CAU)^ and slow growth, which is partially rescued by overexpression of tRNA_i_^Met(CAU)^ [37]. Similarly, *Arabidopsis thaliana trm61* mutants have reduced tRNA_i_^Met(CAU)^ levels, which is associated with shortened siliques [38].

To address the evolutionary role and mechanisms by which m^1^A_58_ influences tRNA_i_^Met(CAU)^ levels and function, we have compared m^1^A_58_ biology in the fission yeast *S*. *pombe* with that in *S*. *cerevisiae*, which diverged ~600 million years ago (Mya) [39]. We show here that the RTD pathway has an unexpected major role in the biology of m^1^A_58_ and tRNA_i_^Met(CAU)^ in both *S*. *pombe* and *S*. *cerevisiae*. We find that *S*. *pombe trm6Δ* mutants lack m^1^A_58_ and are temperature sensitive due to the decay of tRNA_i_^Met(CAU)^ by the RTD pathway, as spontaneous suppressors that restored tRNA_i_^Met(CAU)^ levels had mutations in the *RAT1* ortholog *dhp1* or the *MET22* ortholog *tol1*, whereas mutation of the *TRF4* ortholog *cid14* did not suppress the growth defect. Moreover, we found a major role of the RTD pathway in *S*. *cerevisiae TRM6* biology, as mutation of the RTD pathway components *MET22*, *RAT1*, or *XRN1* each suppressed the temperature sensitivity of *S*. *cerevisiae trm6-504* mutants and restored tRNA_i_^Met(CAU)^ levels. Furthermore, we found that the lethality of *S*. *cerevisiae trm6Δ* mutants was suppressed by inhibition of both the RTD pathway and the nuclear surveillance pathway, but not by inhibition of either pathway alone.

Thus, our results show the importance of tRNA_i_^Met(CAU)^ as a target for m^1^A_58_ modification by Trm6:Trm61 across evolutionarily distant fungal species. Our results also uncover an unexpected conserved evolutionary role of the RTD pathway in tRNA_i_^Met(CAU)^ quality control in *trm6* mutants of both fungal species, as previously found for all three other body modification mutants studied in *S*. *cerevisiae* [16, 20], and the only other body modification mutant studied in *S*. *pombe* [15]. These findings fuel speculation that the RTD pathway has a major role in quality control of other body modification mutants in these organisms, as well as in metazoans.

## Results

### *S. pombe trm6Δ* and *trm61Δ* mutants are temperature sensitive, and lack m^1^A_58_ in their tRNA

**Fig 1:**
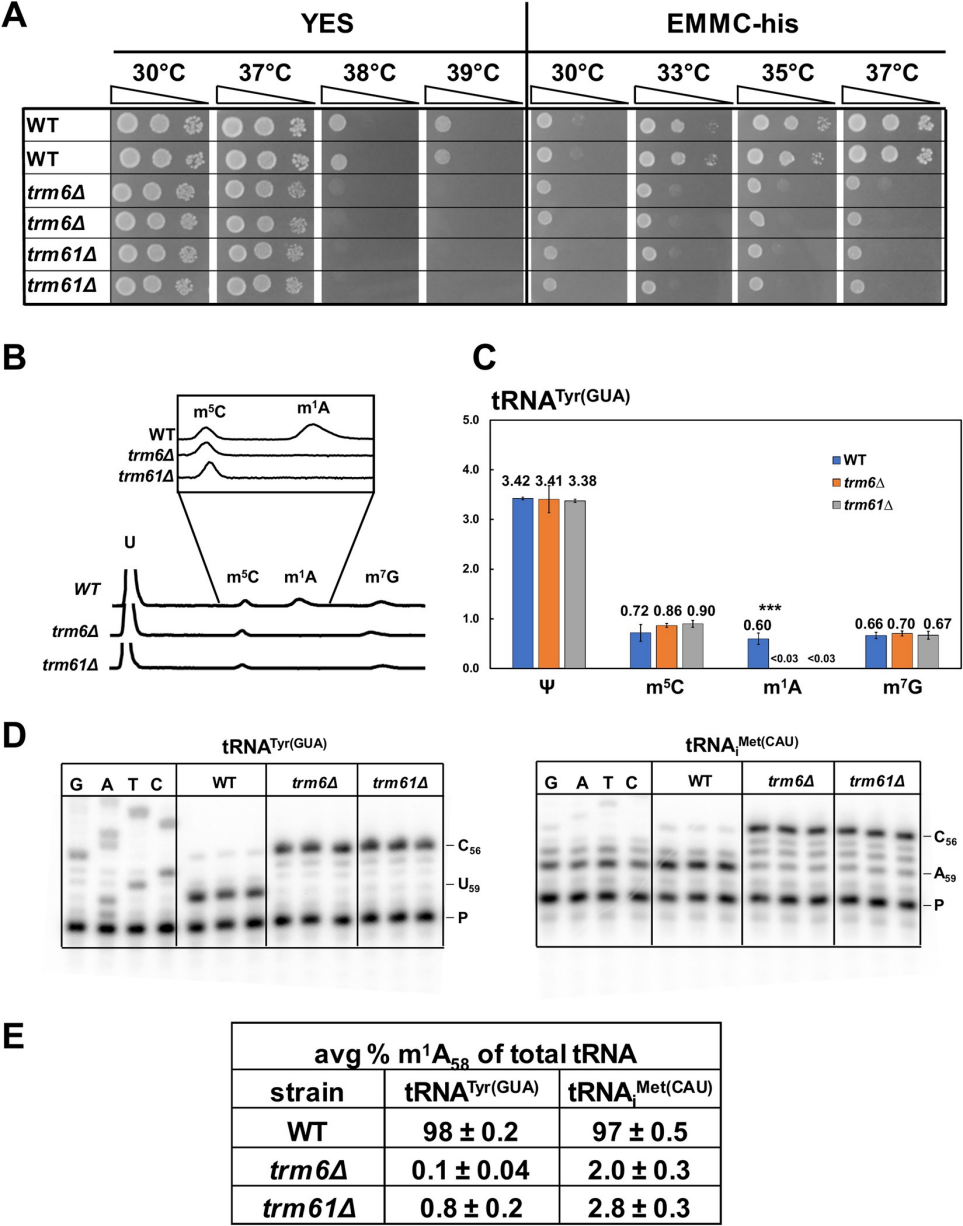
*S*. *pombe trm6Δ* mutants and *trm61Δ* mutants are temperature sensitive and lack m^1^A_58_. ***(A) S*. *pombe trm6Δ* and *trm61Δ* mutants are temperature sensitive on YES and EMMC-his media.** *S*. *pombe trm6Δ* mutants, *trm61Δ* mutants, and WT cells were grown overnight in YES media at 30°C, diluted to OD_600_ ~0.5, serially diluted 10-fold in YES media, and then 2 μL were spotted onto plates containing YES or EMMC-his media and incubated at the indicated temperatures for 3 days. *(****B)* tRNA**^**Tyr(GUA)**^ **from *S*. *pombe trm6Δ* and *trm61Δ* mutants has no detectable m**^**1**^**A, as measured by HPLC separation of nucleosides.** *S*. *pombe trm6Δ* mutants, *trm61Δ* mutants, and WT cells were grown in biological triplicate in YES media at 30°C and tRNA^Tyr(GUA)^ was purified, and digested to nucleosides, and then nucleosides were separated by HPLC as described in Materials and Methods. *(****C)* Quantification of levels of modified nucleosides of purified tRNA**^**Tyr(GUA)**^ **in *S*. *pombe trm6Δ*, *trm61Δ*, and WT strains.** The chart shows average moles/mol of nucleosides with associated standard deviations; WT, blue; *trm6Δ*, orange; *trm61Δ*, gray. *(****D)* tRNA**^**Tyr(GUA)**^ **and tRNA**_**i**_^**Met(CAU)**^ **from *S*. *pombe trm6Δ* and *trm61Δ* mutants have little or no detectable m**^**1**^**A**_**58**_. Bulk RNA from the growth for Fig 1B was analyzed by poison primer extension assay, as described in Materials and Methods, with primer OMT 775 (complementary to tRNA_i_^Met(CAU)^ nt 76–61) and primer OMT 477 (complementary to tRNA^Tyr(GUA)^ 76–61) in the presence of ddGTP. The poison primer extension produces a stop at C_56_ for both tRNA_i_^Met(CAU)^ and tRNA^Tyr(GUA)^, and the presence of m^1^A_58_ results in a stop at N_59_. A sequencing ladder is shown at the left. ***(E)* Quantification of poison primer extension of tRNA**^**Tyr(GUA)**^ **and tRNA**_**i**_^**Met(CAU)**^. For each primer extension, the signals at N_59_ and C_56_ were first corrected by subtraction of the signals at A_58_ and N_57_ respectively.

To begin analysis of the biology of *S*. *pombe* Trm6 and Trm61, we investigated the growth phenotype of *trm6Δ* and *trm61Δ* mutants which, unlike the corresponding *S*. *cerevisiae* mutants, are reported to be viable [40]. To guard against background mutations that might have accumulated in the deletion collection, we first re-made the *trm6Δ* and *trm61Δ* mutants in a wild type (WT) strain, using appropriate kanamycin resistance cassettes, and then compared the growth of two independent *trm6Δ* and *trm61Δ* transformants relative to the WT parent. Each tested *S*. *pombe trm6Δ* and *trm61Δ* mutant grew nearly as well as WT at lower temperatures, but was temperature sensitive on rich (YES) media at 38°C, and on minimal complete media lacking histidine (EMMC-His) at 33°C (Fig 1A). As expected if these *trm6Δ* and *trm61Δ* phenotypes were due to the corresponding deletions, the growth defects were fully complemented after introduction of an *S*. *pombe* [*leu2*^*+*^] plasmid expressing P_*trm6*_ *trm6*^*+*^ or P_*trm61*_ *trm61*^*+*^ respectively (S1A and S1B Fig).

Because the Trm6:Trm61 complex is essential in *S*. *cerevisiae* for m^1^A_58_ modification of substrate tRNAs [21, 41], we examined *S*. *pombe trm6Δ* and *trm61Δ* mutants for m^1^A_58_, to guard against the possibility that the mutants were alive because there is another protein that can catalyze some m^1^A_58_ modification. Examination by HPLC of the nucleoside composition of purified tRNA^Tyr(GUA)^ from *trm6Δ* and *trm61Δ* mutants revealed that m^1^A levels were less than 0.03 moles/mole, compared to 0.60 moles/mole in WT cells, whereas levels of Ψ, m^5^C, and m^7^G were very similar in the tRNA^Tyr(GUA)^ from both mutant and WT cells (Fig 1B and 1C). Poison primer extension of tRNA^Tyr(GUA)^ from WT bulk RNA showed a complete block at U_59_ (98%) due to the presence of m^1^A_58_, which was virtually undetectable in *trm6Δ* and *trm61Δ* mutants (0.1% for *trm6Δ* and 0.8% in *trm61Δ*) (Fig 1D and 1E). Similarly, poison primer extension showed that tRNA_i_^Met(CAU)^ was nearly completely modified with m^1^A_58_ in WT cells (97%), but not visibly modified in *trm6Δ* and *trm61Δ* mutants (although quantification with the high background gave 2.0% for *trm6Δ* and 2.8% in *trm61Δ*). Furthermore, analysis of bulk tRNA modifications revealed that m^1^A modification was less than 0.03% of the levels of cytidine in the *trm6Δ* mutant, compared to 2.5% for WT, whereas levels of Ψ were similar in both strains (16.8% vs 17.9%), as were levels of m^5^C, m^2^G, m^7^G, and inosine (I) (S2 Fig). These results show that *S*. *pombe trm6*^*+*^ and *trm61*^*+*^ are required for all detectable m^1^A_58_ modification of cytoplasmic tRNAs.

### *S. pombe trm6Δ* mutants are temperature sensitive due to reduced levels of tRNA_i_^Met(CAU)^

Since the temperature sensitive growth defect of *S*. *cerevisiae trm6-504* mutants is caused by decreased levels of tRNA_i_^Met(CAU)^ [21], we analyzed levels of tRNA_i_^Met(CAU)^ and other tRNAs in *S*. *pombe trm6Δ* mutants at low and high temperatures to determine if this property was conserved. We sampled cells grown in rich media in triplicate at 30°C and at 3-hour intervals after shift to 38.5°C, and analyzed tRNA levels by northern blot hybridization. We quantified tRNA levels by normalizing relative to tRNA^Gly(GCC)^ at the corresponding temperature and time point, and then relative to the normalized amount in WT cells at 30°C. Note that, as shown previously [15], levels of the usual standards 5S and 5.8S rRNA are significantly affected by temperature changes in *S*. *pombe*, and tRNA^Gly(GCC)^ levels were not affected in WT cells. Note also that in this and all other temperature shift experiments described in this report, cells were shown quantitatively to survive the temperature shift, based on spot tests of the cells on plates.

**Fig 2:**
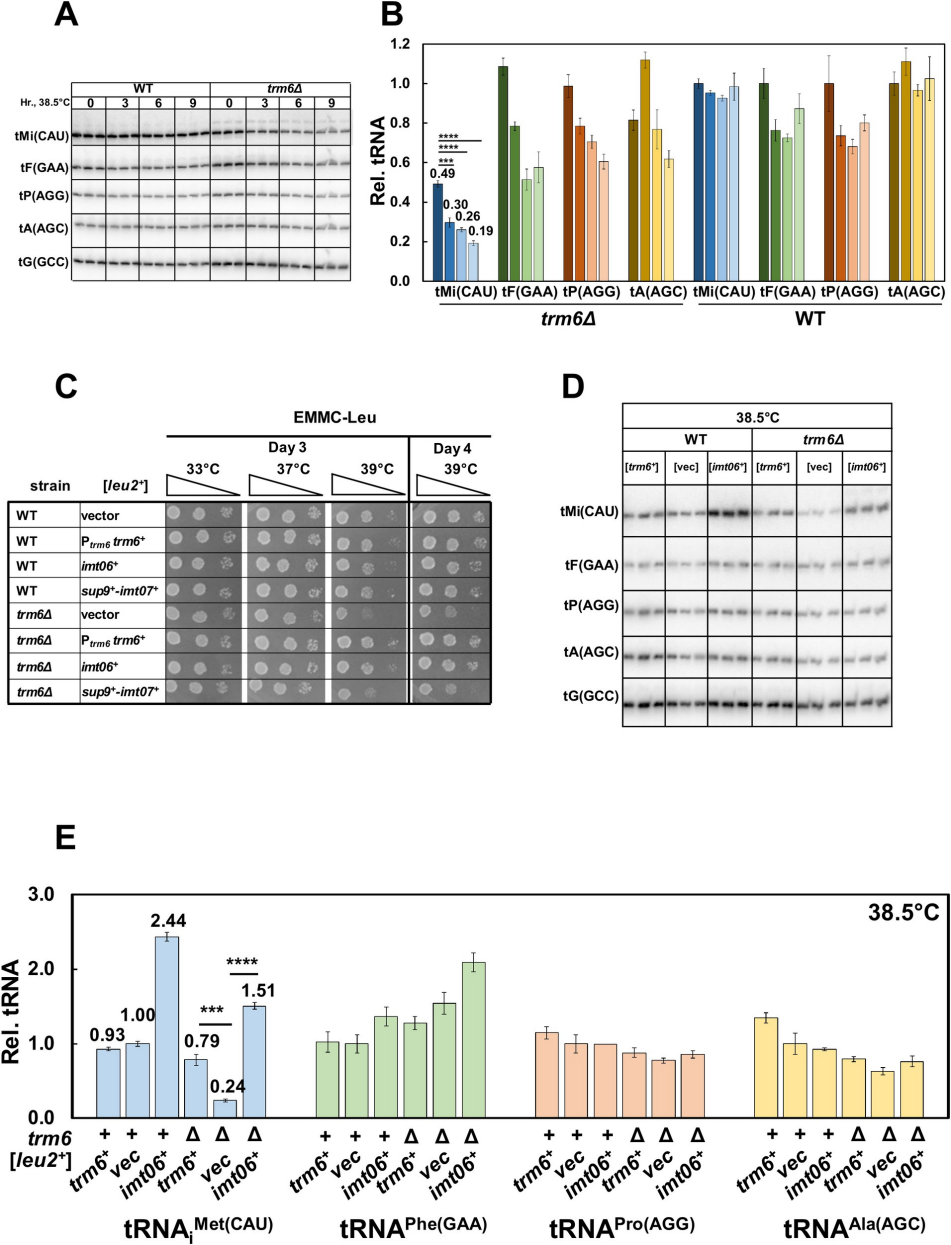
*S*. *pombe trm6Δ* temperature sensitivity is associated with reduced tRNA_i_^Met(CAU)^ levels. ***(A)* Northern analysis of tRNAs in *S*. *pombe trm6Δ* and WT cells before and after shift from 30°C to 38.5°C.** Strains were grown in YES media at 30°C, shifted to 38.5°C for 9 hours as described in Materials and Methods, and RNA was isolated at the indicated times, and analyzed by northern blotting, with probes as indicated. tMi(CAU), tRNA_i_^Met(CAU)^; tF(GAA), tRNA^Phe(GAA)^; tP(AGG), tRNA^Pro (AGG)^; tA(AGC), tRNA^Ala(AGC)^; tG(GCC), tRNA^Gly(GCC)^. ***(B)* Quantification of tRNA levels in *S*. *pombe trm6Δ* and WT cells at 30°C and 38.5°C.** The bar chart depicts relative levels of tRNA species at each temperature, relative to their levels in the WT strain at 30°C (each itself first normalized to levels of the control tG(GCC)). For each tRNA, the dark shade indicates 30°C, and progressively lighter shades indicate time points (3, 6, 9 hours) Standard deviations for each tRNA measurement are indicated. The statistical significance of tRNA levels was evaluated using a two-tailed Student’s t-test assuming equal variance. ns, not significant; *, p *<* 0.05; **, p *<* 0.01; ***, p *<* 0.001; ****, p<0.0001. tMi(CAU), blue; tF(GAA), green; tP(AGG), orange; tA(AGC), yellow. ***(C)* Overproduction of tRNA**_**i**_^**Met(CAU)**^ **suppresses the ts growth defects of *S*. *pombe trm6Δ* mutants.** Strains with plasmids as indicated were grown overnight in EMMC-Leu media at 30°C and analyzed for growth as in Fig 1A on indicated plates and temperatures. ***(D)* Overproduction of tRNA**_**i**_^**Met(CAU)**^ **restores tRNA**_**i**_^**Met(CAU)**^ **levels in *S*. *pombe trm6Δ* mutants.** Strains containing plasmids as indicated were grown in EMMC-Leu at 30°C and shifted to 38.5°C for 8 hours as described in Materials and Methods, and RNA from cells grown at 38.5°C was isolated and analyzed by northern blotting as in Fig 2A. ***(E)* Quantification of tRNA levels in *S*. *pombe trm6Δ* mutants and WT strains overproducing tRNA**_**i**_^**Met(CAU)**^. tRNA levels were quantified as in Fig 2B.

The northern analysis revealed that tRNA_i_^Met(CAU)^ levels were substantially reduced in the *S*. *pombe trm6Δ* mutants, both at 30°C and 38.5°C. At 30°C, tRNA_i_^Met(CAU)^ levels were 49% of those in WT cells, whereas each of the other eight tRNAs had levels between 82% and 121% of those in WT cells (Figs 2A, 2B and S3). At 38.5°C, tRNA_i_^Met(CAU)^ levels decreased further in the *trm6Δ* mutants, to 30% of WT levels after 3 hours, whereas levels of each of the other 8 tRNAs ranged from 78% to 114% of WT levels. Furthermore, although tRNA levels generally decreased after longer times at 38.5°C, tRNA_i_^Met(CAU)^ levels decreased the most, to 19% of those in WT after 9 hours, compared to 53% to 90% for the other eight tRNAs. These results indicate that *S*. *pombe trm6Δ* mutants are associated with reduced levels of tRNA_i_^Met(CAU)^ at 30°C, and with further reduced tRNA_i_^Met(CAU)^ levels at 38.5°C.

To test if the temperature sensitivity of *S*. *pombe trm6Δ* mutants is caused by decreased levels of tRNA_i_^Met(CAU)^, we examined suppression of the *trm6Δ* growth defect upon tRNA_i_^Met(CAU)^ overexpression. In *S*. *pombe*, only one of the four genes encoding tRNA_i_^Met(CAU)^ is expressed in the usual manner, as a stand-alone tRNA gene. By contrast, the other three tRNA_i_^Met(CAU)^ genes are expressed in tandem with a tRNA^Ser^ species as a tRNA^Ser^-tRNA_i_^Met^ dimeric transcript, which is then processed into single tRNAs [42], similar to the tRNA^Arg^-tRNA^Asp^ tandem genes in *S*. *cerevisiae* [43, 44]. To test for suppression of the *trm6Δ* growth defect, we overexpressed either the stand-alone *SPBTRNAMET*.*06 (imt06*^*+*^) gene, or the tandem tRNA^Ser^-tRNA_i_^Met^ gene pair with *sup9*^*+*^ and *SPCTRNAMET*.*07* (*imt07*^*+*^), each on a [*leu2*^*+*^] plasmid. We found that the temperature sensitive growth defect of *S*. *pombe trm6Δ* mutants on EMMC-leu media was completely suppressed by expression of the stand-alone *imt06*^*+*^ gene, growing identically to that of an *S*. *pombe trm6Δ* [P_*trm6*_ *trm6*^*+*^] strain at high temperature (Fig 2C), whereas expression of *imt07*^*+*^ from the tRNA^Ser^-tRNA_i_^Met^ tandem gene only modestly suppressed the *trm6Δ* temperature sensitivity. Consistent with this result, northern analysis showed that levels of tRNA_i_^Met(CAU)^ are much higher in strains overexpressing *imt06*^*+*^ than in strains overexpressing *imt07*^*+*^ (S4 Fig). Similarly, the temperature sensitivity of the *S*. *pombe trm61Δ* strain was completely suppressed by expression of the stand-alone *imt06*^*+*^ gene (S5 Fig). As expected, northern analysis showed that *trm6Δ* strains expressing [*imt06*^*+*^ *leu2*^*+*^] had substantially more tRNA_i_^Met(CAU)^ than the *trm6Δ* [*leu2*^*+*^] vector control strains at 38.5°C (6.3 fold; relative levels of 1.51 vs 0.24) while the levels of other tested tRNAs remained unchanged (Fig 2D and 2E). These results suggest strongly that the temperature sensitivity of *S*. *pombe trm6Δ* mutants is caused by the loss of tRNA_i_^Met(CAU)^ and that this tRNA is the major physiologically important tRNA substrate of Trm6:Trm61 methyltransferase.

### Mutations in the Rapid tRNA decay pathway restore growth and tRNA_i_^Met(CAU)^ levels in *S. pombe trm6Δ* mutants

To identify potential mechanisms that contribute to the loss of tRNA_i_^Met(CAU)^ in *S*. *pombe trm6Δ* mutants, we isolated and analyzed spontaneous suppressors of the temperature sensitivity. Among 25 temperature resistant suppressors from 14 cultures, we found three with increased levels of tRNA_i_^Met(CAU)^ but not of a control tRNA, and whole genome sequencing revealed that two of these had mutations in *dhp1*^*+*^ (*dhp1-5* and *dhp1-6*) and one had a mutation in *tol1*^*+*^ (*tol1-1*). *dhp1*^*+*^ is the ortholog of *S*. *cerevisiae RAT1*, which encodes one of the two 5’-3’ exonucleases involved in RTD, and *tol1*^*+*^ is the ortholog of *S*. *cerevisiae MET22*, deletion of which inhibits RTD by inhibiting 5’-3’ exonucleases [16–18].

**Fig 3:**
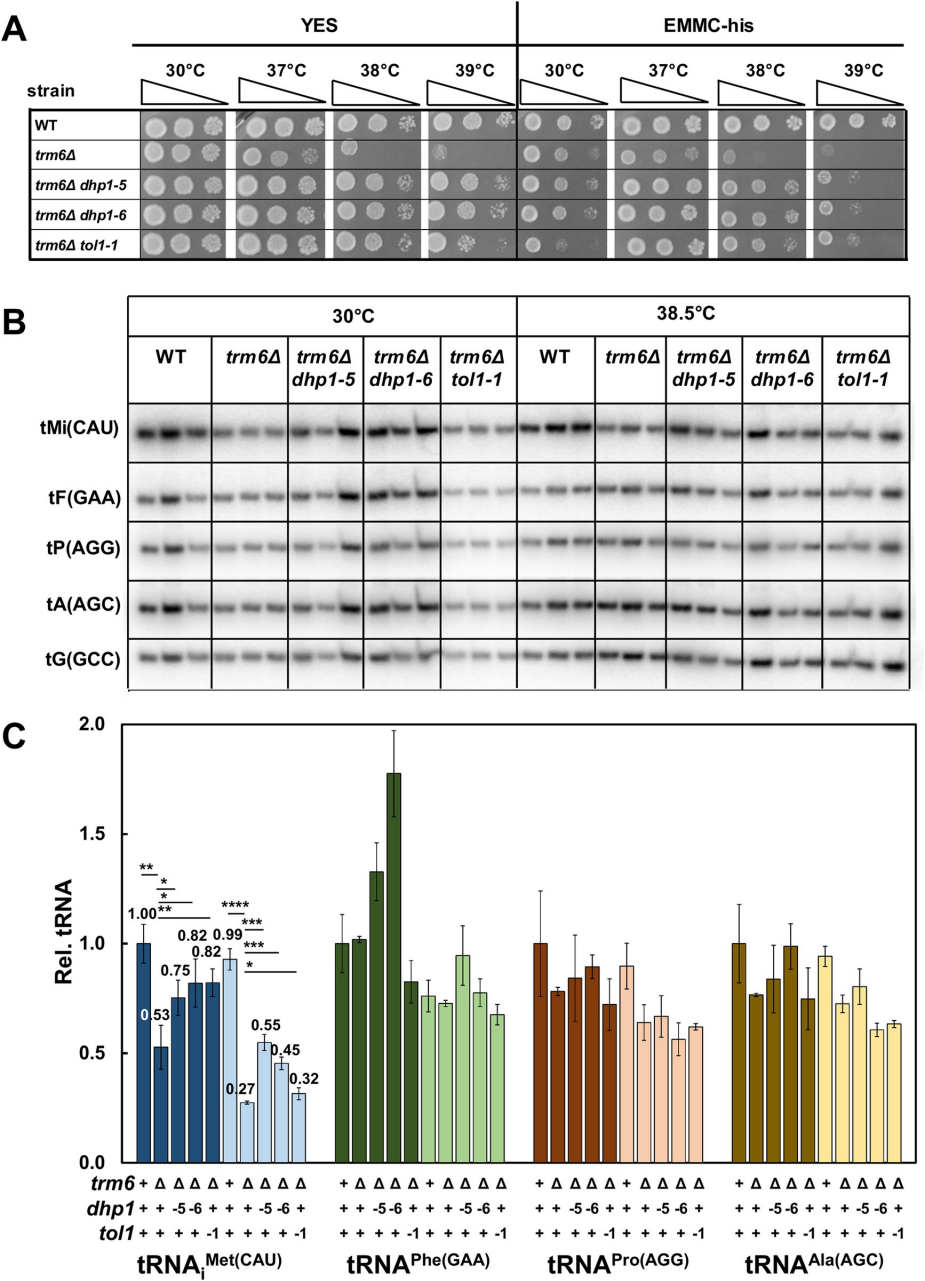
Spontaneous suppressors of *S*. *pombe trm6Δ* mutants with mutations in *dhp1* and *tol1* restore growth and increase tRNA_i_^Met(CAU)^ levels of *S*. *pombe trm6Δ* mutants. ***(A)* Spontaneous suppressors of *S*. *pombe trm6Δ* mutants with mutations in *dhp1* and *tol1* restore growth at high temperatures.** Strains as indicated were grown overnight in YES media at 30°C and analyzed for growth as in Fig 1A on indicated plates and temperatures. ***(B)* Spontaneous suppressors of *S*. *pombe trm6Δ* mutants with mutations in *dhp1* and *tol1* restore tRNA**_**i**_^**Met(CAU)**^ **levels after growth at 38.5°C.** Strains were grown in YES media at 30°C and shifted to 38.5°C for 8 hours as described in Materials and Methods, and RNA was isolated and analyzed by northern blotting as in Fig 2A. ***(C)* Quantification of tRNA levels of *S*. *pombe trm6Δ dhp1* and *tol1* mutants.** tRNA levels were quantified as in Fig 2B, with dark and light shades as indicated for 30°C and 38.5°C.

Growth analysis on plates showed that the *trm6Δ dhp1-5* and *trm6Δ dhp1-6* mutants were nearly as healthy at high temperatures as the WT strain on both YES and EMMC-his media, whereas the *trm6Δ tol1-1* mutant was slightly less healthy at higher temperatures (Fig 3A). Northern analysis of tRNA from strains grown at 30°C and after temperature shift to 38.5°C showed that the *dhp1* and *tol1* suppressors substantially restored tRNA_i_^Met(CAU)^ levels at both high and low temperatures, without affecting any of a number of other tRNAs (Fig 3B and 3C). At 38.5°C, tRNA_i_^Met(CAU)^ levels increased from 27% in *trm6Δ* strains (relative to WT at 30°C) to 55%, 45%, and 32% in the *trm6Δ dhp1-5*, *trm6Δ dhp1-6*, and *trm6Δ tol1-1* mutants respectively, with no significant change in the levels of tRNA^Phe(GAA)^, tRNA^Pro(AGG)^, and tRNA^Ala(AGC)^ (Fig 3B and 3C). This increase in tRNA_i_^Met(CAU)^ levels at 38.5°C accounts for the temperature resistance of the strains, and reflects the weaker suppression in the *trm6Δ tol1-1* strain. At 30°C, tRNA_i_^Met(CAU)^ levels also increased, from 53% of WT in the *trm6Δ* strains to 75%, 82%, and 82% in the *trm6Δ dhp1-5*, *trm6Δ dhp1-6*, and *trm6Δ tol1-1* mutants, again with little change in the levels of other tRNAs. Thus, it appears that the observed decay is occurring at both temperatures.

Two lines of evidence suggest that the *dhp1* mutations were responsible for the suppression in the *trm6Δ dhp1* mutants. First, the isolation of two different *dhp1* missense mutations in genetically independent suppressors argues strongly that the relevant suppressing mutation is that in *dhp1*. Whole genome sequencing typically results in only a few mutations, and finding two independent suppressors with different *dhp1* mutations would be highly unlikely to occur by chance. Furthermore, like *S*. *cerevisiae RAT1*, *dhp1*^*+*^ is an essential gene in *S*. *pombe*, and thus there are limited mutations that would reduce function without killing the cell. Indeed, alignments show that neither of the *dhp1* mutations is completely conserved, although each is likely to be important; the S737P (*dhp1-5*) mutation is predicted to disrupt the central portion of an α-helix, and the Y669C (*dhp1-6*) mutation is within a highly conserved block of amino acids (S6A and S6B Fig). Second, complementation experiments showed that introduction of an additional chromosomal copy of *dhp1*^*+*^ at *ura4*^*+*^ restored the temperature sensitivity of the *trm6Δ dhp1-5* mutant, to a level similar to that of a *trm6Δ* control strain. The increased gene dosage of *dhp1*^*+*^ had no effect on growth of the WT strain, and a barely detectable inhibitory effect on growth of the *trm6Δ* strain (S6C Fig), which we attribute to the 2-fold overproduction of Dhp1 and the presumed sensitivity of the *trm6Δ* strain to any further reduction in tRNA_i_^Met(CAU)^ levels. We therefore conclude that the isolated *dhp1* mutations were responsible for the suppression of the temperature sensitive growth defect of *S*. *pombe trm6Δ* mutants.

Similarly, we infer that the *tol1-1* mutation is responsible for suppression because expression of P_*tol1*_ *tol1*^*+*^ on a [*leu2*^*+*^] plasmid restored temperature sensitivity to the *trm6Δ tol1-1* strain, with no effect on growth of the *trm6Δ* or the WT strain (S7 Fig). The *tol1-A151D* (*tol1-1*) point mutation is located in a highly conserved region of the essential *tol1*^*+*^ gene [45], presumably resulting in a partial loss of function variant (S8A and S8B Fig).

The discovery of *dhp1* and *tol1* mutations as suppressors of the *S*. *pombe trm6Δ* temperature sensitivity demonstrates the involvement of the RTD pathway in decay of tRNA_i_^Met(CAU)^ lacking m^1^A_58_ in *S*. *pombe*. However, this result was unexpected, because it is well established in *S*. *cerevisiae* that *trm6* mutants trigger decay of pre-tRNA_i_^Met(CAU)^ by the nuclear surveillance pathway in vivo [13, 22, 24, 46] and in vitro [47].

### The exacerbated growth defect of an *S. pombe trm6Δ imt06Δ* mutant is due to further reduction in tRNA_i_^Met(CAU)^ levels, and suppressors of the growth defect are in the RTD pathway

**Fig 4:**
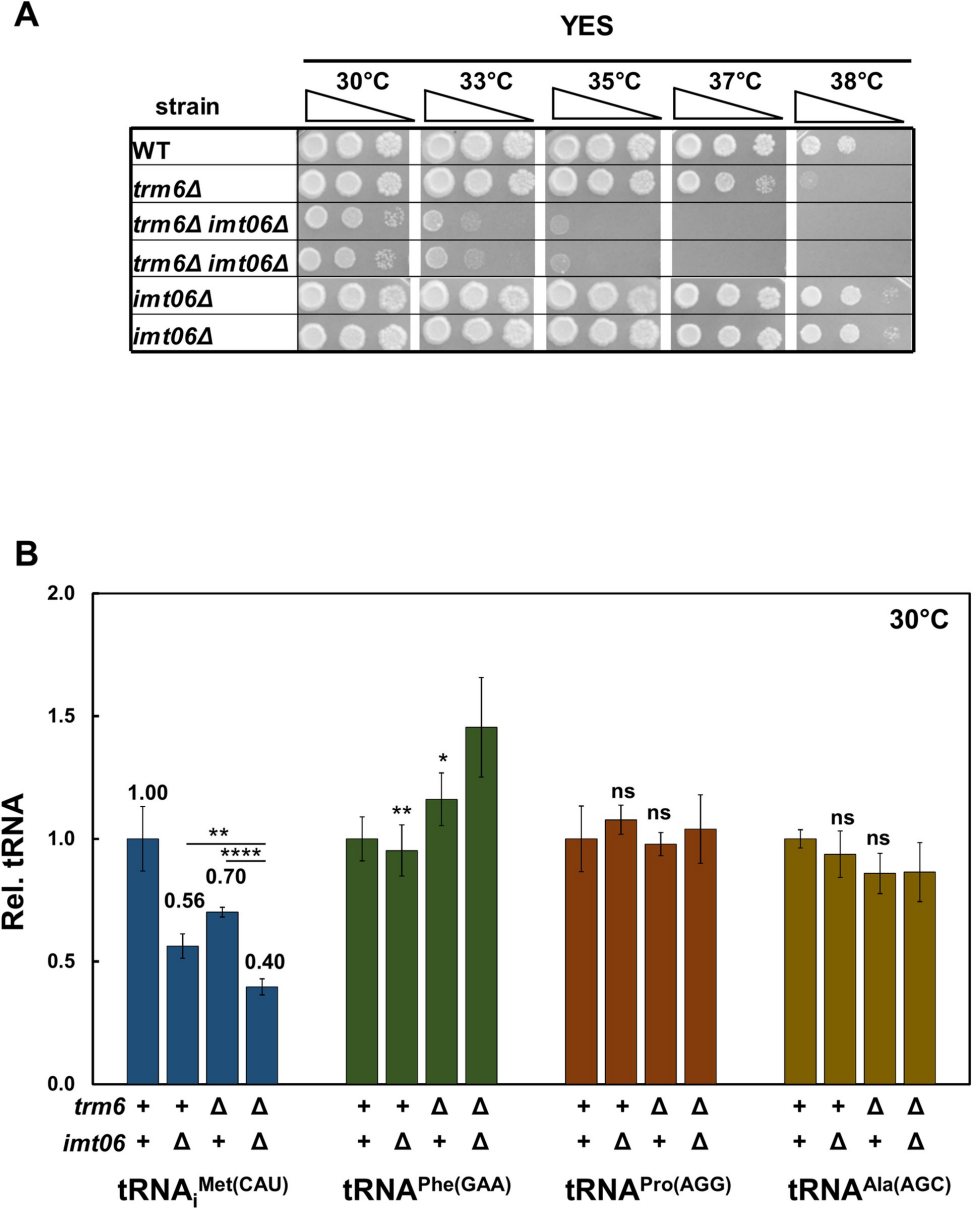
Deletion of one of the four *S*. *pombe* genes encoding tRNA_i_^Met(CAU)^ in an *S*. *pombe trm6Δ* mutant exacerbates its growth defect and further reduces tRNA_i_^Met(CAU)^ levels. ***(A)* Deletion of the *imt06***^***+***^ **gene encoding tRNA**_**i**_^**Met(CAU)**^ **in an *S*. *pombe trm6Δ* mutant severely exacerbates its growth.** Strains were grown overnight in YES media at 30°C and analyzed for growth as in Fig 1A on indicated plates and temperatures for 4 days. ***(B)* Quantification of tRNA**_**i**_^**Met(CAU)**^ **levels in *S*. *pombe trm6Δ imt06Δ* mutants at 30°C.** tRNA levels were quantified as in Fig 2B.

To obtain a more robust set of suppressors of the *trm6Δ* temperature sensitivity, we further reduced tRNA_i_^Met(CAU)^ levels in the *trm6Δ* mutants by introduction of an *imt06Δ* mutation, decreasing the number of tRNA_i_^Met(CAU)^ genes from four to three. As anticipated, the resulting *trm6Δ imt06Δ* strain grew very poorly at 30°C, and was temperature sensitive at higher temperatures, not growing at all at 37°C, whereas the *imt06Δ* mutant had no growth defect at any tested temperature (Figs 4A and S9A). Moreover, the *trm6Δ imt06Δ* growth defect was strictly due to the loss of tRNA_i_^Met(CAU)^ because the *trm6Δ imt06Δ* strains expressing both an integrated copy of *imt06*^*+*^ and a [*leu2*^*+*^ *imt06*^*+*^] plasmid grew as well as WT on YES media at all temperatures up to 39°C (S9B Fig). As anticipated, tRNA_i_^Met(CAU)^ levels in the *trm6Δ imt06Δ* strain at 30°C were substantially reduced from those in the *trm6Δ* mutants (40% vs 70% of WT), consistent with the 44% reduction in tRNA_i_^Met(CAU)^ levels in the *imt06Δ* strains (Figs 4B and S9C). As also expected, the levels of other tested tRNAs in these strains were virtually unaffected. These results show a prominent synthetic growth defect in the *S*. *pombe trm6Δ imt06Δ* strain, due only to reduced levels of tRNA_i_^Met(CAU)^.

**Fig 5:**
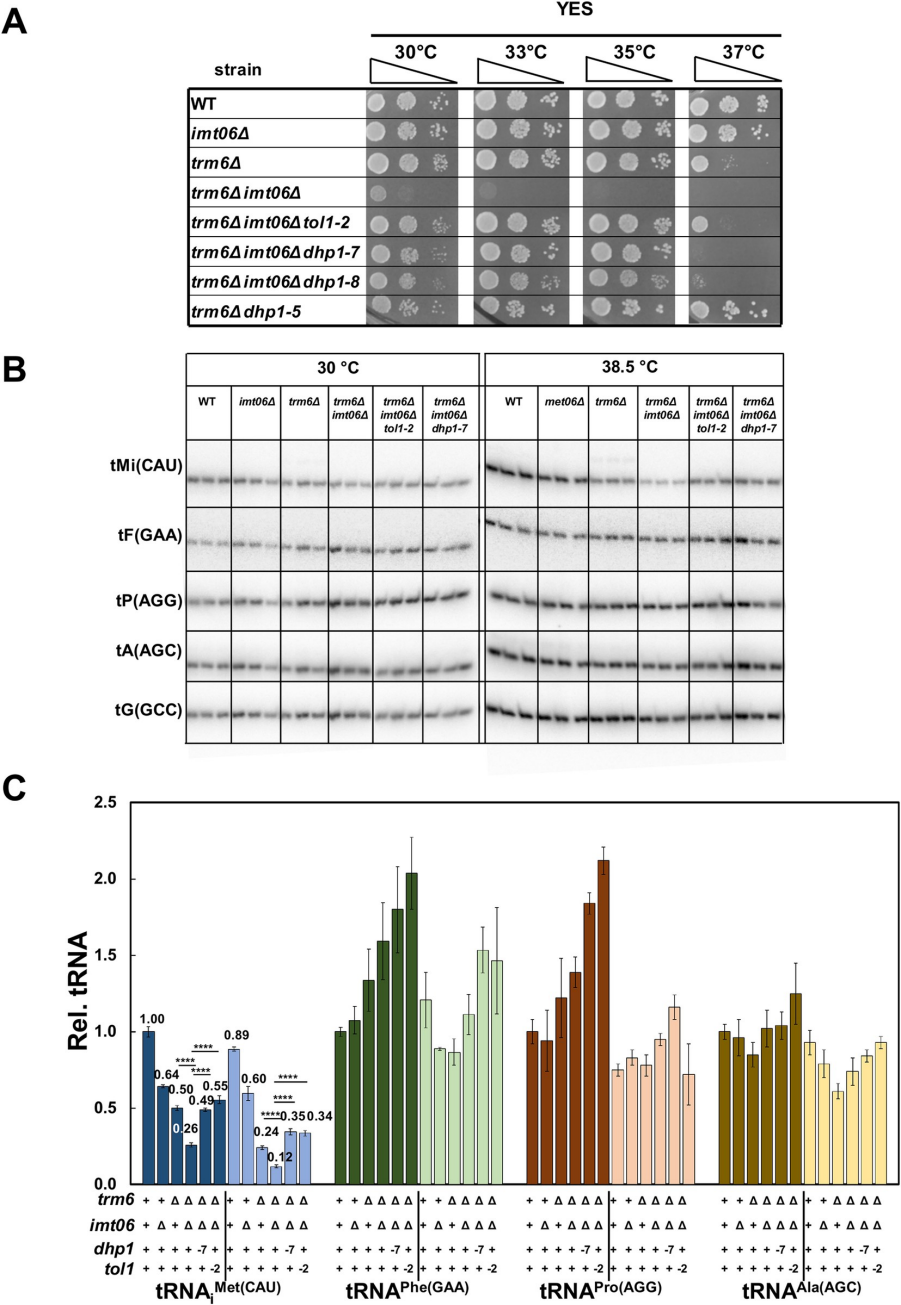
Spontaneous suppressors of *S*. *pombe trm6Δ imt06Δ* mutants with mutations in *dhp1* and *tol1* restore growth and increase tRNA_i_^Met(CAU)^ levels. ***(A)* Spontaneous suppressors of *S*. *pombe trm6Δ imt06Δ* mutants with mutations in *dhp1* and *tol1* suppress the growth defect.** Strains were grown overnight in YES media at 30°C and analyzed for growth as in Fig 1A on indicated plates and temperatures. ***(B)* Spontaneous suppressors of *S*. *pombe trm6Δ imt06Δ* mutants with mutations in *dhp1* and *tol1* restore tRNA**_**i**_^**Met(CAU)**^ **levels at low and high temperatures.** Strains were grown in YES media at 30°C and shifted to 38.5°C for 6 hours as described in Materials and Methods, and RNA was isolated and analyzed by northern blotting as in Fig 2A. ***(C)* Quantification of tRNA**_**i**_^**Met(CAU)**^ **levels in *S*. *pombe trm6Δ imt06Δ dhp1-7* and *trm6Δ imt06Δ tol1-2* mutants.** tRNA levels were quantified as in Fig 2B.

Analysis of spontaneous suppressors of the severe growth defect of *trm6Δ imt06Δ* mutants revealed additional mutations in the RTD pathway. Of fifteen suppressors isolated from six independent cultures of *trm6Δ imt06Δ* strains after plating on YES media at 35°C, eleven had increased tRNA_i_^Met(CAU)^ levels at both 30°C and 38.5°C, relative to a control tRNA, and whole genome sequencing of nine of these eleven suppressors revealed eight with *dhp1* mutations (six alleles; *dhp1-7* to *dhp1-12*), and one with a *tol1* mutation (*tol1-2*). All six new *dhp1* mutations and the *tol1-2* mutation were in conserved regions of the respective proteins (S10 Fig). Growth comparisons showed that the *dhp1-7*, *dhp1-8*, and *tol1-2* mutations each efficiently rescued the *trm6Δ imt06Δ* growth defect at 35°C, with the *trm6Δ imt06Δ tol1-2* strain growing almost as well as the original *trm6Δ* strain at 37°C (Fig 5A). Moreover, the growth phenotype of the *trm6Δ imt06Δ tol1-2* mutant was fully complemented upon introduction of a [*leu2*^*+*^ P_*tol1*_ *tol1*^*+*^] plasmid (S11 Fig). Consistent with the growth suppression, tRNA_i_^Met(CAU)^ levels were increased from 26% of WT in the *trm6Δ imt06Δ* mutant at 30°C to 49% and 55% in the corresponding *dhp1-7* and the *tol1*-2 suppressors, and from 12% at 38.5°C to 35% and 34% in the suppressors, whereas control tRNA levels were largely unchanged at each temperature (Fig 5B and 5C).

These findings underscore the crucial role of Dhp1 and Tol1, and thus of the RTD pathway, in quality control of tRNA_i_^Met(CAU)^ in *S*. *pombe trm6Δ* mutants. Indeed, the isolation of eight genetically distinct *dhp1/rat1* alleles (two in *trm6Δ* mutants and six in *trm6Δ imt06Δ* mutants) and two distinct *tol1/met22* alleles (one each in the *trm6Δ* mutant and the *trm6Δ imt06Δ* mutant) argues that the genetic landscape of *trm6Δ* suppressors has been nearly saturated, particularly considering that both *dhp1* and *tol1* are essential in *S*. *pombe*.

Further examination suggests the lack of participation of the Trf4 ortholog Cid14 of the nuclear surveillance pathway [48–50] in quality control of tRNA_i_^Met(CAU)^ in *S*. *pombe trm6Δ* mutants. A *cid14Δ* mutation was introduced into WT and *trm6Δ* strains and independent isolates were confirmed by PCR (Materials and Methods), after which the resulting strains were shown to be sensitive to 5-fluorouracil (5-FU) (S12A and S12B Fig), as previously reported [48, 51]. We observed little, if any, suppression of the *trm6Δ* growth defect in the *trm6Δ cid14Δ* strains (S13A Fig), and only very minor restoration of tRNA_i_^Met(CAU)^ levels at high temperature, relative to levels in *trm6Δ* mutants (21% vs 18%, compared to 39% in the *trm6Δ dhp1-5* strain) (S13B and S13C Fig). Thus, we infer that tRNA_i_^Met(CAU)^ is degraded in *S*. *pombe trm6Δ* and *trm6Δ imt06Δ* mutants primarily by the RTD pathway, and not appreciably by the TRAMP complex of the nuclear surveillance pathway.

### A *met22Δ* mutation substantially suppresses the *S. cerevisiae trm6-504* temperature sensitivity and partially restores tRNA_i_^Met(CAU)^ levels at low and high temperatures

**Fig 6:**
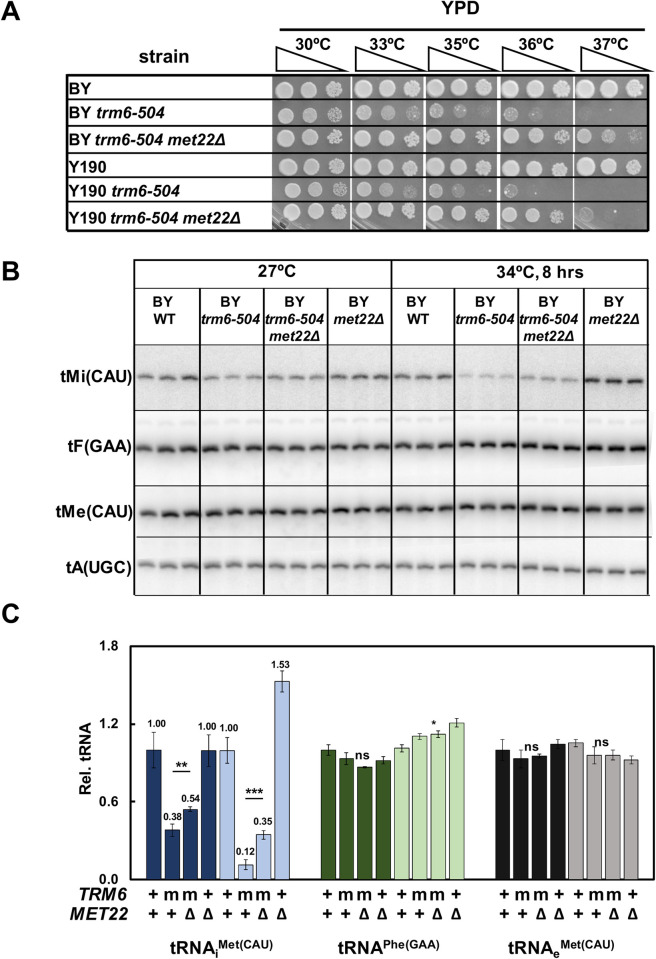
The temperature sensitivity and reduced tRNA_i_^Met(CAU)^ levels in *S*. *cerevisiae trm6-504* mutants are suppressed by a *met22Δ* mutation. ***(A)* A *met22Δ* mutation substantially suppresses the *S*. *cerevisiae trm6-504* temperature sensitivity.** Strains were grown overnight in YPD media at 30°C and analyzed for growth on indicated plates and temperatures. BY; standard BY4741 WT strain background; Y190, background of original *trm6-504* mutant ***(B)* A *met22Δ* mutation substantially restores tRNA**_**i**_^**Met(CAU)**^ **levels in *S*. *cerevisiae trm6-504* mutants.** Strains were grown in YPD media at 27°C and shifted to 34°C for 8 hours as described in Materials and Methods, and RNA was isolated and analyzed by northern blotting. ***(C)* Quantification of northern analysis of tRNA**_**i**_^**Met(CAU)**^ **levels in *S*. *cerevisiae* BY *trm6-504* mutants.** tRNA levels were quantified as in Fig 2B. m, *trm6-504* mutant.

Because of our discovery of the predominant role of the RTD pathway in tRNA_i_^Met(CAU)^ quality control in *S*. *pombe trm6Δ* mutants, we examined the participation of the RTD pathway in tRNA_i_^Met(CAU)^ quality control in the well-studied *S*. *cerevisiae trm6-504*^*ts*^ mutant, which had previously been shown to trigger tRNA_i_^Met(CAU)^ decay by the nuclear surveillance pathway [13, 22, 23]. We found that deletion of *MET22* substantially suppressed the temperature sensitive growth defect of an *S*. *cerevisiae trm6-504*^*ts*^ mutant, both in its original background (Y190) and in the BY4741 (BY) background, with obvious suppression at 36°C in both backgrounds and at 37°C in the BY background (Fig 6A). Consistent with the growth suppression, tRNA_i_^Met(CAU)^ levels were increased from 12% of WT in the BY *trm6-504* strain to 35% in the *met22Δ* derivative at 34°C, and from 38% to 54% at 27°C, with little effect on other tested tRNAs (Fig 6B and 6C). Similar restoration of tRNA_i_^Met(CAU)^ levels was observed in the *met22Δ* derivative of the original Y190 *trm6-504* strain, with no effect on other tested tRNAs (S14 Fig). These results show that *MET22* regulates tRNA_i_^Met(CAU)^ levels in *trm6-504* strains regardless of their genetic background and suggest the involvement of the RTD pathway in tRNA_i_^Met(CAU)^ quality control in *S*. *cerevisiae trm6-504* mutants.

### Each of the RTD pathway exonucleases has a significant role in tRNA_i_^Met(CAU)^ quality control in *S. cerevisiae* BY *trm6-504* mutants

Although the restoration of growth and tRNA_i_^Met(CAU)^ levels in a BY *trm6-504 met22Δ* mutant suggested the involvement of the RTD pathway, we sought to provide additional evidence by directly testing the roles of the RTD pathway exonucleases Rat1 and Xrn1 in tRNA_i_^Met(CAU)^ quality control in the *trm6-504* mutant. As Rat1 is essential [52], we tested the role of Rat1 using the *rat1-107* mutation, which we had previously isolated as a suppressor of RTD in *trm8Δ trm4Δ* mutants [16].

**Fig 7:**
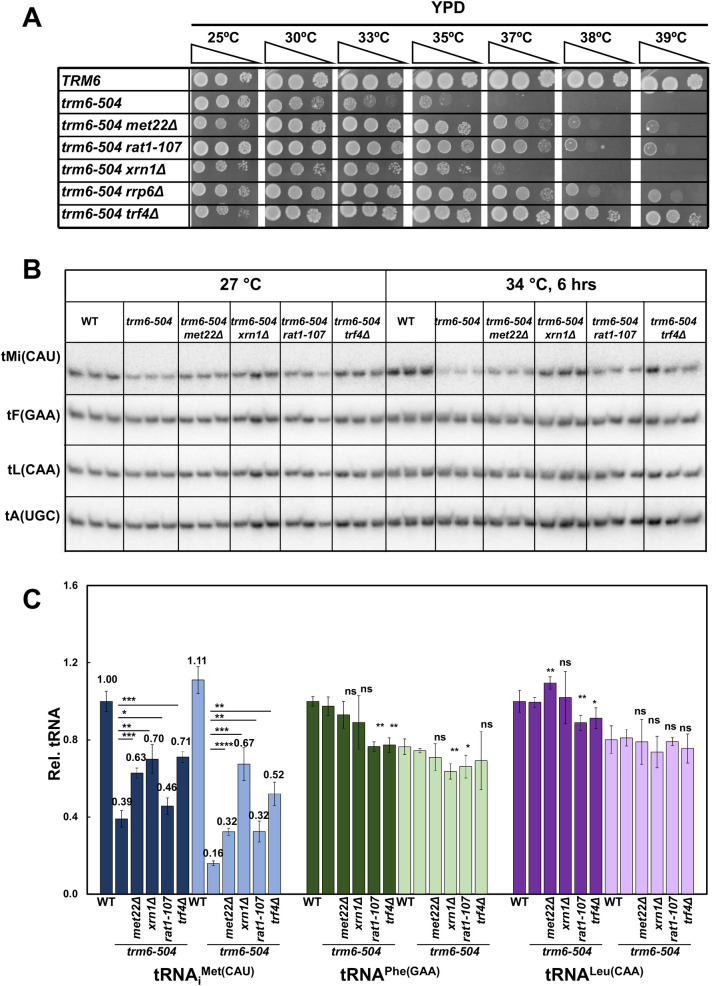
The RTD and nuclear surveillance pathways are each involved in tRNA_i_^Met(CAU)^ quality control in *S*. *cerevisiae trm6-504* mutants. ***(A)* The *S*. *cerevisiae trm6-504* mutant growth defect is substantially suppressed by mutations in individual components of the RTD and nuclear surveillance pathways.** Strains were grown overnight in YPD media at 30°C and analyzed for growth on indicated plates and temperatures. ***(B)* tRNA**_**i**_^**Met(CAU)**^ **levels are substantially restored in *S*. *cerevisiae trm6-504* strains with mutations in individual components of the RTD and nuclear surveillance pathways.** Strains were grown in YPD at 27°C and shifted to 34°C for 6 hours as described in Materials and Methods, and RNA was isolated and analyzed by northern blotting. ***(C)* Quantification of tRNA**_**i**_^**Met(CAU)**^ **levels in *S*. *cerevisiae trm6-504* strains with mutations in the RTD and nuclear surveillance pathways.** Dark and light colors indicate growth at 27°C and 34°C.

We found that mutation of each of the RTD exonucleases efficiently suppressed both the temperature sensitivity and the reduced tRNA_i_^Met(CAU)^ levels of the BY *trm6-504* mutant. Whereas the *trm6-504* mutant was impaired for growth at 33°C and above, the *trm6-504 rat1-107* strain had healthy growth at 37°C and visible growth at 39°C, which was similar to that of the *trm6-504 met22Δ* strain, and the *trm6-504 xrn1Δ* strain grew up to 37°C (Fig 7A), despite the known growth defect of *xrn1Δ* mutants [16]. This growth suppression by mutation of each RTD component was nearly as efficient as that due to mutation of the nuclear surveillance components *RRP6* or *TRF4* (Fig 7A) [13]. Moreover, consistent with the suppression results, the temperature dependent decay of tRNA_i_^Met(CAU)^ in *trm6-504* mutants was efficiently suppressed by mutation of RTD components. Thus, after 6-hour temperature shift to 34°C, relative tRNA_i_^Met(CAU)^ levels in *trm6-504* mutants were restored from 16% to 32%, 67%, and 32% by *met22Δ*, *xrn1Δ*, and *rat1-107* mutations respectively, comparable to the 52% observed in a *trm6-504 trf4Δ* mutant (Fig 7B and 7C). Significant suppression of tRNA_i_^Met(CAU)^ levels was also found at 27°C. A parsimonious interpretation of these results is that all components of the RTD pathway are involved in tRNA_i_^Met(CAU)^ quality control in *trm6-504* mutants, and that the nuclear surveillance pathway and RTD pathway each contribute substantially to this tRNA_i_^Met(CAU)^ quality control.

### tRNA_i_^Met(CAU)^ in *S. cerevisiae trm6-504* mutants and suppressors is fully modified to m^1^A_58_ at both low and high temperatures

As *trm6-504* mutants are known to have reduced, but not absent, m^1^A modification levels [13], we wanted to determine if m^1^A levels were altered in tRNA_i_^Met(CAU)^ as a result of the temperature shift in *trm6-504* mutants. By using poison primer extension to measure m^1^A_58_ modification, we found that A_58_ of tRNA_i_^Met(CAU)^ was nearly fully modified at both 27°C and 34°C in both *trm6-504* mutants (96% and 94%) and WT strains (98% and 97%) (S15 Fig), although tRNA_i_^Met(CAU)^ levels are reduced in *trm6-504* mutants. By contrast, A_58_ of tRNA^Phe(GAA)^ was substantially hypomodified at low temperature in *trm6-504* mutants compared to WT strains (25% vs 83%), and also at high temperature (27% vs 68%) (S15 Fig). As tRNA_i_^Met(CAU)^ is 96% modified at low temperature and present at 39% of WT levels, whereas tRNA^Phe(GAA)^ is 25% modified and present at 97% of WT levels, these findings suggest that tRNA_i_^Met(CAU)^ is the preferred substrate of Trm6:Trm61. In addition, comparison of the tRNA^Phe(GAA)^ modification levels in *trm6-504* mutants at low and high temperature suggests that there is little or no temperature-dependent reduction in the Trm6:Trm61 methyltransferase activity.

To further assess the connection between m^1^A_58_ modification and tRNA decay, we measured m^1^A levels in tRNA_i_^Met(CAU)^ in *trm6-504* strains with mutations in the nuclear surveillance or the RTD pathway. We found that tRNA_i_^Met(CAU)^ was still nearly fully modified to m^1^A_58_ at 34°C in *trm6-504* mutants with suppressing mutations in any of the components of the RTD pathway (*met22Δ*, *rat1-*107 or *xrn1Δ*) or the nuclear surveillance pathway (*trf4Δ* or *rrp6Δ*), with modification levels ranging from 92.6% to 95.2%, compared to 97.6% in WT cells (S16A and S16C Fig). As anticipated, A_58_ modification of tRNA^Phe(GAA)^ was similarly reduced in *trm6-504* mutants and in derivatives with suppressing mutations in the RTD or nuclear surveillance pathway, compared to WT cells (S16B and S16C Fig). Thus, although tRNA_i_^Met(CAU)^ decay at 34°C is inhibited in *trm6-504* strains with mutations in the nuclear surveillance or the RTD pathway, the nearly complete modification of the remaining, undegraded tRNA_i_^Met(CAU)^ in all of these *trm6-504* derivative strains argues for competition between the Trm6:Trm61 enzyme and the decay pathways.

### The lethality of *S. cerevisiae trm6Δ* mutants is suppressed by mutation of both the RTD and the nuclear surveillance pathways, but not either one alone

**Fig 8:**
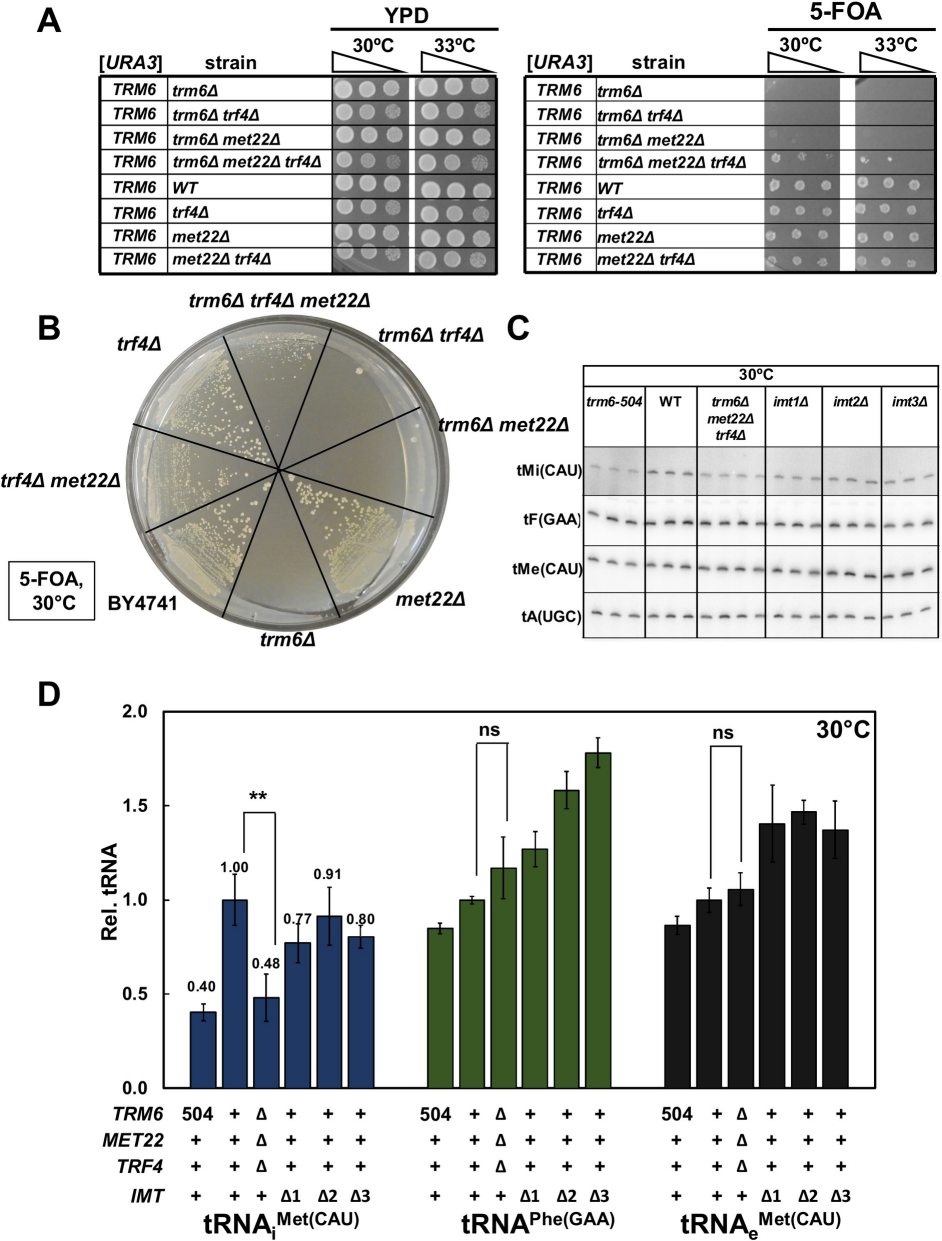
The lethality of an *S*. *cerevisiae trm6Δ* mutant is suppressed by deletion of both *MET22* and *TRF4*, but not by either deletion alone, and results in modest tRNA_i_^Met(CAU)^ levels. ***(A)* Growth test on plates of an *S*. *cerevisiae trm6Δ* [*URA3 TRM6*] strain with a *met22Δ* and/or *trf4Δ* mutation.** Strains were grown overnight in YPD media at 30°C, diluted to OD_600_~3, serially diluted 10-fold in water, and 2 μL were spotted onto YPD or 5-FOA plates as indicated, and grown for 4 days. ***(B)* Pie streak growth test of an *S*. *cerevisiae trm6Δ* [*URA3 TRM6*] strain with a *met22Δ* and/or *trf4Δ* mutation.** Strains were grown overnight in YPD media at 30°C, and then 2 μl of each of the cultures was streaked on 5-FOA plates for single colonies, and then incubated at 30°C for 7 days. ***(C)* Northern analysis of tRNA**_**i**_^**Met(CAU)**^ **in *S*. *cerevisiae trm6Δ met22Δ trf4Δ* mutants.** Strains were grown in YPD at 30°C for 6 hours as described in Materials and Methods, and RNA was isolated and analyzed by northern blotting. ***(D)* Quantification of tRNA**_**i**_^**Met(CAU)**^ **levels in *S*. *cerevisiae trm6Δ met22Δ trf4Δ* mutants.** tRNA levels were quantified as in Fig 2B.

To separate the effects of the decay pathways from the presumed competition with Trm6:Trm61, we determined if the lethality of *S*. *cerevisiae trm6Δ* mutants could be rescued by inhibition of either or both of the RTD and nuclear surveillance pathways. As previously shown, the *S*. *cerevisiae trm6Δ* lethality is due to lack of tRNA_i_^Met(CAU)^ [21], as a *trm6Δ* [*TRM6 URA3*] [2μ *IMT1 LEU2*] strain was healthy when the *URA3* plasmid was selected against on media containing 5-FOA, but the corresponding strain with a [2μ *LEU2*] vector died (S17 Fig). We found that deletion of both *MET22* and *TRF4* suppressed the lethality of the *S*. *cerevisiae trm6Δ* mutant on 5-FOA-containing media at 30°C and 33°C, but neither single deletion could rescue the lethality of the *trm6Δ* mutant alone (Fig 8A and 8B). Thus, we conclude that both the RTD pathway and the nuclear surveillance pathway significantly contribute to tRNA_i_^Met(CAU)^ quality control in mutants lacking m^1^A_58_ modification. As tRNA_i_^Met(CAU)^ levels in *trm6Δ met22Δ trf4Δ* strains were only 48% of WT levels, comparable to the tRNA_i_^Met(CAU)^ levels in *trm6-504* mutants and somewhat less than tRNA_i_^Met(CAU)^ levels in mutants lacking one of the four *IMT* genes (Fig 8C and 8D), we infer that tRNA_i_^Met(CAU)^ lacking m^1^A_58_ modification is still being degraded in this strain.

## Discussion

We have provided strong evidence that the rapid tRNA decay pathway has a major role in decay of tRNA_i_^Met(CAU)^ lacking m^1^A_58_ in both *S*. *pombe* and *S*. *cerevisiae*. In *S*. *pombe*, *dhp1* and *tol1* mutations in the RTD pathway suppress the temperature sensitivity and decay of tRNA_i_^Met(CAU)^ in *trm6Δ* and *trm6Δ imt06Δ* mutants. In *S*. *cerevisiae*, *met22*, *rat1*, and *xrn1* mutations in the RTD pathway suppress the temperature sensitivity and tRNA_i_^Met(CAU)^ decay in *trm6-504* mutants, and a *met22Δ* mutation is required, together with a *trf4Δ* mutation in the nuclear surveillance pathway, to restore viability of *S*. *cerevisiae trm6Δ* mutants.

The finding that reduced tRNA_i_^Met(CAU)^ levels are the cause of the defect in both *S*. *pombe* and *S*. *cerevisiae trm6* mutants lacking m^1^A_58_ is consistent with the unique structural properties of eukaryotic tRNA_i_^Met(CAU)^ in the D-loop and the T-loop. In this region, eukaryotic initiator tRNA differs from that of canonical elongator tRNAs in the absence of N_17_, the replacement of canonical residues with A_20_, A_54_, and A_60_, and a unique substructure involving these residues and m^1^A_58_, as well as G_57_ and A_59_ [26, 53]. As Trm6:Trm61 are conserved in eukaryotes [38, 54], we infer that tRNA_i_^Met(CAU)^ levels will be similarly subject to decay in other eukaryotes lacking m^1^A_58_.

These findings establish that the RTD pathway acts on all body modification mutants that have been shown to result in decay of tRNAs in fungi, including *S*. *cerevisiae* mutants lacking m^7^G_46_, ac^4^C_12_, or m^2,2^G_26_, particularly in combination with other body modification mutants [16, 20], *S*. *pombe* mutants lacking m^7^G_46_ [15], and now mutants lacking m^1^A_58_ in both organisms. This set of results suggests that the RTD pathway will mediate decay of other body modification mutants in *S*. *pombe* and *S*. *cerevisiae*. Furthermore, as *S*. *cerevisiae* and *S*. *pombe* diverged about 600 Mya [39], these findings suggest that body modification mutants in other eukaryotes will also undergo decay by the RTD pathway. In support of this suggestion, we note that Rat1/Dhp1, Met22/Tol1, and Xrn1 of the RTD pathway are all conserved in eukaryotes [55, 56], and that WT HeLa cells (without a modification defect) that are incubated at 43°C undergo decay of tRNA_i_^Met(CAU)^ by Rat1 and Xrn1 [35]. As a subset of hypomodified tRNAs are known to be reduced in mammalian cells lacking m^7^G_46_ [57, 58] or m^5^C [59], it is likely that tRNA decay is occurring in these cells, and based on our results, we speculate that this decay is due to the RTD pathway.

It was surprising to find that the decay of tRNA_i_^Met(CAU)^ in *S*. *pombe trm6Δ* mutants was not due to the nuclear surveillance pathway, because of its well established role in decay of tRNA_i_^Met(CAU)^ in *S*. *cerevisiae trm6-504* mutants [13, 22, 23]. We argued above that in *S*. *pombe*, tRNA_i_^Met(CAU)^ lacking m^1^A_58_ is primarily degraded by the RTD pathway, because we had essentially saturated the genetic landscape of suppressors of *S*. *pombe trm6Δ* or *trm6Δ imt06Δ* strains with mutations in *dhp1* or *tol1* of the RTD pathway, and because a *cid14Δ* mutation in the nuclear surveillance pathway did not restore growth or tRNA_i_^Met(CAU)^ levels to an *S*. *pombe trm6Δ* mutant. It is known that the other components of the nuclear surveillance pathway are present and functional in *S*. *pombe* [48, 49, 60, 61]. It is possible that the lack of participation of the nuclear surveillance pathway in decay of tRNA_i_^Met(CAU)^ in *S*. *pombe trm6Δ* mutants is due in some way to the structure of three of the four tRNA_i_^Met(CAU)^ genes, each of which is present as a dimeric tRNA gene, and expressed as a tandem tRNA^Ser^- tRNA_i_^Met^ transcript that is then processed into individual tRNAs [42]. Alternatively, it is possible that the lack of participation of the nuclear surveillance pathway in *S*. *pombe trm6Δ* mutants is due to some, as yet unappreciated, difference in the structure or folding between *S*. *pombe* and *S*. *cerevisiae* tRNA_i_^Met(CAU)^, or to differences in the activity of the nuclear surveillance pathway.

Our finding that *dhp1* and *tol1* mutations significantly restore tRNA_i_^Met(CAU)^ levels at 30°C and 38.5°C in *S*. *pombe trm6Δ* and *trm6Δ imt06Δ* mutants underscores that the RTD pathway is active at all temperatures in *S*. *pombe*, as we also found in *S*. *cerevisiae trm6-504* mutants, and previously found in *S*. *cerevisiae* mutants lacking m^7^G_46_ and m^5^C [16] and in fully modified variants of tRNA^Tyr^ [62]. The relatively healthy growth of *S*. *pombe trm6Δ* mutants and the poor growth of *trm6Δ imt06Δ* mutants at 30°C, with tRNA_i_^Met(CAU)^ levels at ~50% and 26% of WT respectively, is consistent with prior results that *S*. *cerevisiae* strains are healthy with three of four tRNA_i_^Met(CAU)^ genes, generally grow slowly with two of four genes, and can survive with as little as 40% of WT fully modified tRNA_i_^Met(CAU)^ [63, 64].

Our results provide evidence that the nuclear surveillance and the RTD pathways in *S*. *cerevisiae* are in competition with the Trm6:Trm61 m^1^A methyltransferase, as tRNA_i_^Met(CAU)^ was fully modified in *S*. *cerevisiae trm6-504* mutants at both 27°C and 34°C, and inhibition of either decay pathway resulted in more tRNA_i_^Met(CAU)^ that was fully modified. Moreover, as Trm6:Trm61 is a nuclear enzyme [21], and Xrn1 is cytoplasmic [65], the increased levels of fully modified tRNA_i_^Met(CAU)^ in *trm6-504 xrn1Δ* mutants argues that unmodified tRNA_i_^Met(CAU)^ is not immediately degraded, but rather that tRNA_i_^Met(CAU)^ goes to the cytoplasm without m^1^A and returns to the nucleus for another chance at m^1^A modification by Trm6:Trm61. This second chance for m^1^A modification is analogous to the second chance pathway suggested earlier for tRNAs lacking m^2,2^G_26_ [66].

Our finding that tRNA^Phe(GAA)^ in *S*. *cerevisiae trm6-504* mutants had very similar m^1^A_58_ modification at both 27°C and 34°C suggests that Trm6:Trm61 is not temperature sensitive in this strain. If so, the reduced tRNA_i_^Met(CAU)^ levels at high temperature in *trm6-504* mutants would imply that tRNA_i_^Met(CAU)^ lacking m^1^A_58_ is itself temperature sensitive, perhaps becoming partially unfolded at high temperature. One could envision a model in which tRNA_i_^Met(CAU)^ lacking m^1^A_58_ is functioning in the cell cytoplasm (consistent with the viability of *S*. *pombe trm6Δ* mutants and of *S*. *cerevisiae trm6Δ trf4Δ met22Δ* mutants), but is in equilibrium with a state in which the tertiary structure is partially or completely unfolded due to lack of the modification [21, 26]. As tertiary structure unfolding precedes unfolding of the individual helices of tRNA [67], the disrupted tertiary structure due to lack of m^1^A_58_ could lead to unfolding of the acceptor stem and increased availability of the 5’ end to the RTD pathway. This model is very similar to that we proposed previously to explain the increased Xrn1 susceptibility of *S*. *cerevisiae* tRNA^Ser(CGA)^ lacking ac^4^C_12_ and Um_44_ and for tRNA^Val(AAC)^ lacking m^7^G_46_ and m^5^C_49_ [68], and could be tested in subsequent experiments.

## Materials and methods

### Yeast strains

All *S*. *pombe* and *S*. *cerevisiae* strains with integrated markers that are described in this work were made in biological triplicate. *S*. *pombe* strains are shown in S1 Table. *S*. *pombe trm6Δ*::*KanMX* strains were constructed in the *S*. *pombe* WT strain derived from SP286 (*ade6-M210/ade6-M216*, *leu1-32/leu1-32*, *ura4-D18/ura4-D18 h+/h+*) by PCR amplification of the *trm6Δ*::*kanMX* cassette from the *S*. *pombe trm6Δ*::*kanMX* strain of the genomic knockout collection [40], followed by linear transformation using lithium acetate [69], and PCR screening of transformants for the presence of the deletion. Other *S*. *pombe* deletion strains were made similarly, but the DNA containing the drug marker (*KanMX* or *HygR*) was obtained by Gibson assembly of ~ 500 nt 5’ of the target site, the drug marker, and ~ 500 nt 3’ of the target site [70]. The *dhp1*^*+*^ and *imt06*^*+*^ genes were integrated at the chromosomal *ura4-D18* locus using a single *ura4*^*+*^integrating vector containing the corresponding gene under its promoter [71]. *S*. *cerevisiae* deletion strains are shown in S2 Table, and were constructed by linear transformation with PCR amplified DNA from the appropriate knockout strain, followed by PCR amplification to confirm the knockout.

The *S*. *cerevisiae trm6-504* mutant strain was obtained in two ways. We obtained the original *trm6-504* (Y200) and its WT parent (Y190) from Dr. James Anderson. The BY *trm6-504* strain was reconstructed essentially as previously described [72], with three DNA components constructed in a plasmid vector: first, nt 893–1434 of the *TRM6* coding sequence (containing the C1292G mutation of the *trm6-504* mutant) and 204 nt of the 3’ UTR; second, *K*. *lactis URA5*; third, nt 1384–1434 of the *TRM6* coding region. The DNA construct was removed from the vector, transformed into *S*. *cerevisiae* WT cells by linear transformation, confirmed by PCR, and then strains were plated onto media containing 5-FOA to select for Ura^-^ cells obtained by homologous recombination, which were sequence verified.

### Plasmids

Plasmids used in this study are listed in S3 Table. The *S*. *pombe* plasmid expressing *S*. *pombe* P_*trm6*_ *trm6*^*+*^, *S*. *pombe* P_*trm61*_ *trm61*^*+*^, and P_*tol1+*_ *tol1*^*+*^ contained ~ 1000 bp and 500 bp of flanking 5’ and 3’ DNA and were cloned into a pREP3X-derived plasmid, removing the P_*nmt1*_ promoter, as described [15]. Plasmids expressing *S*. *pombe* tRNA genes contained ~ 300 bp of flanking 5’ and 3’ DNA. The *S*. *pombe ura4*^*+*^ single integrating vectors [71] expressing *imt06*^*+*^ or P_*dhp1+*_ *dhp1*^*+*^ were constructed similarly.

### Yeast media and growth conditions

*S*. *pombe* strains were grown in rich (YES) media or Edinburgh minimal complete (EMMC) media, or corresponding dropout media, as described [15]. For temperature shift experiments, cells were grown in YES or EMMC-leu media at 30°C to OD_600_ ~ 0.5, and then diluted to OD_600_ 0.1 in pre-warmed media at the desired temperature, and grown to OD_600_ ~ 0.5, and then aliquots were chilled, harvested at 4°C, washed with ice cold water, frozen on dry ice, and stored at -80°C. *S*. *cerevisiae* strains were grown in rich (YPD) media or minimal complete (SDC) media as described [15], and temperature shift experiments were performed as described for *S*. *pombe*. All experiments with measurements were performed in biological triplicate.

### Spontaneous suppressor isolation

Spontaneous suppressors of *S*. *pombe trm6Δ* and *trm6Δ imt06Δ* mutants were isolated by growing cultures of individual colonies in YES media at 30°C, followed by plating 10^7^ cells on YES and EMMC plates at 39°C (for *trm6Δ* mutants) and on YES plates at 35°C (for *trm6Δ imt06Δ* mutants).

### Bulk RNA preparation and northern blot analysis

For northern analysis, biological triplicates were grown in parallel, aliquots of 3–5 OD were harvested, and then bulk RNA was prepared with glass beads and phenol as described [73], resolved on a 10% polyacrylamide (19:1), 7M urea, 1X TBE gel, transferred to Amersham Hybond-N+ membrane (Cytiva, Marlborough, MA cat# RPN303B), and hybridized with 5’ ^32^P-labeled DNA probes (S4 Table) as described [14], followed by exposure and imaging on an Amersham Typhoon phosphorimager (Cytiva, Marlborough, MA), and quantification using Image Quant v5.2

### Isolation and purification of bulk tRNA

*S*. *pombe* WT and *trm6Δ* mutant strains were grown to ~ 0.5 OD in YES media at 30°C, and then bulk low molecular weight RNA was extracted from ~ 300 OD of pellets by using hot phenol [74], and resolved on an 8% polyacrylamide (19:1) 7M urea, 1X TBE gel to purify bulk tRNA by elution of tRNA from the excised gel slice.

### Isolation and purification of tRNA^Tyr(GUA)^

tRNA^Tyr(GUA)^ was purified from *S*. *pombe* WT strains, and *trm6Δ* and *trm61Δ* mutant strains using 1 mg of bulk RNA (prepared using hot phenol), and the 5’-biotinylated oligonucleotide (TDZ 365; tY(GUA) 76–64; 5’ TGGTCTCCTGAGCCAGAATCGAACTA 3’), as described [74].

### HPLC analysis of nucleosides

Purified tRNA^Tyr(GUA)^ (~ 1.25 μg) was digested to nucleosides by treatment with P1 nuclease, followed by phosphatase, as described [74], and nucleosides were analyzed by HPLC (Waters, Millford, MA) at pH 7.0 as described [75]. To quantify nucleosides in bulk tRNA, relative amounts of modified nucleosides were compared to cytidine.

### Poison primer extension assays

Oligomers used for primer extension are shown in S5 Table. Primers were 5’-end labeled essentially as described [74], with excess label removed using a MicroSpin G-25 chromatography column (Cytiva, Marlborough, MA cat#27532501), and poison primer extension was done as described [76], in 10 μL reactions containing 2 U AMV reverse transcriptase (Promega, Madison, WI cat# M5101), 1X AMV RT buffer, 1 mM ddNTP, and 1 mM of the other three dNTPs. Following extension for 1 h at 50°C, aliquots were resolved on a 15% polyacrylamide gel (29:1) containing 7 M urea in 1× TBE, and the gel was dried on a Model 583 Biorad gel dryer, exposed and analyzed on an Amersham Typhoon phosphorimager, and quantified using Image Quant v5.2.

### Whole genome sequencing

Whole genome sequencing was performed by the University of Rochester Genomics Center at 25–50 fold coverage of the genome, and reads were compared to the corresponding parent strain, and to the reference genome.

## Supporting information

S1 Fig The temperature sensitivity of *S*. *pombe trm6Δ* and *trm61Δ* mutants is complemented by expression of the corresponding gene. ***(A)* The temperature sensitivity of an *S*. *pombe trm6Δ* mutant is complemented by [P**_***trm6***_ ***trm6***^***+***^ ***leu2***^***+***^**] on EMMC-leu media.** WT and *trm6Δ* cells expressing P_*trm6*_ *trm6+* were grown overnight in EMMC-leu media 30°C and analyzed for growth at the indicated temperatures. ***(B)* The temperature sensitivity of *S*. *pombe trm61Δ* is complemented by [P**_***trm61***_ ***trm61***^***+***^ ***leu2***^***+***^**] on EMMC-leu media.** WT and *trm61Δ* cells expressing P_*trm61*_ *trm61+* were grown overnight in EMMC-leu media 30°C and analyzed for growth.
(PDF)

S2 Fig *S*. *pombe trm6Δ* mutants lack m^1^A in their bulk tRNA. ***(A*,*B)* Bulk tRNA from *S*. *pombe trm6Δ* mutants have no detectable m**^**1**^**A.** *S*. *pombe trm6Δ* mutants and WT cells were grown in biological triplicate in YES media at 30°C and bulk tRNA was purified, digested to nucleosides, and analyzed for modifications by HPLC as described in Materials and Methods. ***(A)* A trace of the A**^**258 nm**^ **of eluted nucleosides of bulk tRNA.** *(****B)* Quantification of levels of modified nucleosides of purified tRNA**^**Tyr(GUA)**^. The bar chart depicts the average moles/mol of nucleosides (expressed as a percentage of the moles of cytidine), with associated standard deviation; WT, gray; *S*. *pombe trm6Δ*, red. The data is also tabulated in the table below *(****C)***.
(PDF)

S3 Fig Northern analysis of all tested tRNAs in *S*. *pombe trm6Δ* and WT cells after shift from 30°C to 38.5°C. **(A) Northern blot.** Full analysis is shown of tRNAs analyzed in the northern blot shown in Fig 2A. ***(B*,*C)* Quantification of tRNA levels.** The bar chart depicts relative levels of tRNA species at each temperature, relative to their levels in WT at 30°C.
(PDF)

S4 Fig Northern analysis of tRNA_i_^Met(CAU)^ levels in WT and *trm6Δ* strains expressing *imt06*^*+*^ or *sup9*^*+*^*-imt07*^*+*^ from a [*LEU2*] plasmid. **(A) Northern blot.** Strains were grown and analyzed as in Fig 2D. **(B) Quantification of tRNA levels.** tRNA levels were quantified as in Fig 2B.
(PDF)

S5 Fig Overproduction of tRNA_i_^Met(CAU)^ suppresses the temperature sensitive growth defects of *S*. *pombe trm61Δ* mutants. Strains with plasmids as indicated were grown overnight in EMMC-Leu media at 30°C and analyzed for growth as in Fig 1A on indicated plates and temperatures.
(PDF)

S6 Fig The *dhp1-5* and *dhp1-6* mutations that suppress the *S*. *pombe trm6Δ* growth defect disrupt conserved regions or structures of Dhp1. ***(A)* Alignment of regions around the *dhp1-5* (*S737P)* and *dhp1-6* (*Y669C)* mutations.** *S*. *pombe* Dhp1 was aligned with putative Rat1/Dhp1 orthologs from 12 evolutionarily distinct eukaryotes, using Multalin [77]; http://multalin.toulouse.inra.fr/multalin/). red, more than 80% conservation; blue 40% - 80% conservation. ***(B)* Location of *dhp1-5* (*S737P)* and *dhp1-6* (*Y669C)* mutations mapped onto the *S*. *pombe* structure [78].** magenta, residues in the catalytic center; blue, residues interacting with Rai1. ***(C)* Expression of P**_***dhp1***_ ***dhp1+* integrated in the chromosome restores temperature sensitive growth of the *S*. *pombe trm6Δ dhp1-5* mutant.** WT, *trm6Δ*, and *trm6Δ dhp1-5* cells expressing a chromosomally integrated copy of P_*dhp1*_ *dhp1+* in the *ura4+* locus or the control vector integrant, were grown overnight in YES media at 30°C, and analyzed for growth.
(PDF)

S7 Fig The suppression of the *S*. *pombe trm6Δ* growth defect by a *tol1-1* mutation is complemented by expression of *tol1* on a [P_*tol1*_ *tol1*^*+*^ *leu2*^*+*^] plasmid. *trm6Δ tol1-1* mutants, *trm6Δ* mutants and WT strains were transformed with either [P_*tol1*_ *tol1*^*+*^ *leu2*^*+*^] or empty vector, grown in EMMC-leu, and spotted.
(PDF)

S8 Fig The isolated *tol1-1* mutation that restores growth of an *S*. *pombe trm6Δ* mutant is in a conserved region of the protein. ***(A)* Alignment of the regions around the *tol1-1* (*A151D)* mutation.** *S*. *pombe* Tol1 was aligned with putative Tol1 orthologs from 12 evolutionarily distinct eukaryotes, as in S5 Fig. red, more than 80% conservation; blue 40% - 80% conservation. ***(B)* Location of *tol1-1* (*A151D)* mapped onto the structure of the *S*. *cerevisiae* ortholog Met22 [79].** orange, active site residues.
(PDF)

S9 Fig Deletion of one of the four *S*. *pombe* genes encoding tRNA_i_^Met(CAU)^ in an *S*. *pombe trm6Δ* mutant exacerbates its growth defect and further reduces tRNA_i_^Met(CAU)^ levels. ***(A)* Deletion of the *imt06* gene encoding tRNA**_**i**_^**Met(CAU)**^ **in an *S*. *pombe trm6Δ* mutant severely exacerbates its growth.** Strains from the growth test in Fig 4A are shown after 2 days of growth ***(B)* Complementation of *trm6Δ imt06Δ growth defect* with an integrated *imt06* and a [*leu2***^***+***^ ***imt06***^***+***^**] plasmid.** *trm6Δ imt06Δ* and WT cells expressing tRNA_i_^Met(CAU)^ from a chromosomally integrated copy of *imt06+* and from a [*leu2*^*+*^ *imt06+*] plasmid, and controls were grown overnight in EMMC or EMMC-leu media at 30°C, and analyzed for growth. ***(C)* Levels of tRNA**_**i**_^**Met(CAU)**^ **are significantly reduced in *S*. *pombe trm6Δ imt06Δ* mutants at 30°C.** The Northern blot from Fig 4B is shown.
(PDF)

S10 Fig Suppressor mutations isolated in *S*. *pombe trm6Δ imt06Δ* strains are in conserved regions of Dhp1 and Tol1. *(A)* Alignment of regions around the *dhp1* suppressor mutations. The alignment of *S*. *pombe* Dhp1 was done as in S5A Fig. ***(B)* Location of *dhp1* suppressor mutations mapped onto the *S*. *pombe* structure [78].** magenta, residues in the catalytic center; blue, residues interacting with Rai1. ***(C)* Alignment of the regions around the *tol1-2* (*A297D)* mutation.** The alignment of *S*. *pombe* Tol1 was done as in S7 Fig. ***(D)* Location of *tol1-2* (*A297D)* mapped onto the structure of the *S*. *cerevisiae* ortholog Met22 [79].** orange, active site residues.
(PDF)

S11 Fig Expression of [P_*tol1*_ *tol1*^*+*^ *leu2*^*+*^] fully complements the *S*. *pombe trm6Δ imt06Δ tol1-2* mutants. WT, *trm6Δ imt06Δ*, and *trm6Δ imt06Δ tol1-2* cells expressing P_*tol1*_ *tol1+* or a vector [80] were grown overnight in EMMC-Leu media at 30°C, and analyzed for growth
(PDF)

S12 Fig A *cid14Δ* mutation causes 5-FU sensitivity in *S*. *pombe* WT and *trm6Δ* strains. ***(A)* Analysis of growth of *cid14Δ* strains on YES media with or without 5-FU.** Strains were grown overnight in YES media at 30°C and analyzed for growth as in Fig 1A on indicated plates and temperatures. ***(B)* Complementation of the 5-FU sensitivity of *cid14Δ* strains.** Strains were grown overnight in EMMC-Leu media at 30°C and analyzed for growth on indicated plates and temperatures.
(PDF)

S13 Fig A *cid14Δ* mutation does not suppress the growth defect of *S*. *pombe trm6Δ* mutants and has only a minimal effect on tRNA_i_^Met(CAU)^ levels. ***(A)* A *cid14Δ* mutation does not suppress the growth defect of *S*. *pombe trm6Δ* mutants.** Strains were grown overnight in YES media at 30°C and analyzed for growth as in Fig 1A on indicated plates and temperatures. ***(B*,*C)* A *cid14Δ* mutation has only a minimal effect on tRNA**_**i**_^**Met(CAU)**^ **levels in *S*. *pombe trm6Δ* mutants.**
(PDF)

S14 Fig A *met22Δ* mutation partially restores tRNA_i_^Met(CAU)^ levels in *S*. *cerevisiae* Y190 *trm6-504* mutants. Strains were grown in YPD at 27°C and shifted to 33°C for 6 hours as described in Materials and Methods, and RNA was isolated and analyzed by northern blotting. ***(A)* Northern Blot. *(B)* Quantification of northern.** B; standard BY4741 WT strain background; Y, Y190 background of original *trm6-504* mutant; m, *trm6-504* mutant.
(PDF)

S15 Fig In *S*. *cerevisiae trm6-504* mutants, tRNA_i_^Met(CAU)^ is fully modified to m^1^A_58_ at 27°C and 34°C, while tRNA^Phe(GAA)^ is hypomodified to a similar extent at both temperatures. ***(A)* Primer extension analysis of m**^**1**^**A**_**58**_ **modification in tRNA**_**i**_^**Met(CAU)**^ **and tRNA**^**Phe(GAA)**^. Bulk RNA from *S*. *cerevisiae trm6-504* mutants and WT cells grown for Fig 7B was analyzed by poison primer extension assay, as described in Materials and Methods, with the P1 primer (complementary to tRNA_i_^Met(CAU)^ nt 76–61) and P2 primer (complementary to tRNA^Phe(GAA)^ 76–60) in the presence of ddCTP, producing a stop at G_57_ for both tRNA_i_^Met(CAU)^ and tRNA^Phe(GAA)^, and a stop at N_59_ for m^1^A_58_. ***(B)* Quantification of the poison primer extension.** Values were calculated by first subtracting background levels, as in Fig 1E.
(PDF)

S16 Fig In *S*. *cerevisiae trm6-504* mutants grown at 34°C, tRNA_i_^Met(CAU)^ is fully modified to m^1^A_58_ in derivatives with mutations in the RTD and nuclear surveillance pathways. ***(A)* Primer extension analysis of m**^**1**^**A**_**58**_ **modification in tRNA**_**i**_^**Met(CAU)**^. Bulk RNA from the growth done for Fig 7B was analyzed by poison primer extension assay with the P1 primer in the presence of ddATP, producing a stop at U_55_ or at A_59_ for m^1^A_58_. ***(B)* Primer extension analysis of m**^**1**^**A**_**58**_ **modification in tRNA**^**Phe(GAA)**^. Bulk RNA from the growth done for Fig 7B was analyzed by poison primer extension assay with the P2 primer in the presence of ddCTP, producing a stop at G_57_ and for m^1^A_58_ at U_59_. ***(C)* Quantification of the data from (*A*) and (*B*).**
(PDF)

S17 Fig Overexpression of *S*. *cerevisiae IMT1* fully suppresses *trm6Δ* mutant lethality. *S*. *cerevisiae* WT, *trm6-504*, and *trm6Δ* strains containing [*2μ* P_*GAL*_*TRM6 URA3*] plasmid [81] and [*2μ IMT1 LEU2*] plasmids or empty vector, as indicated, were grown overnight in SD-leu media at 30°C and analyzed by spotting on SD-Leu media containing 5-FOA. Then cells from the 5-FOA plates were streaked for colonies, inoculated into SD-Leu media and grown overnight, and re-spotted on SD-Leu media.
(PDF)

S1 Table *S*. *pombe* strains used in this study. (PDF)

S2 Table *S*. *cerevisiae* strains used in this study. (PDF)

S3 Table Plasmids used in this study. (PDF)

S4 Table Oligomers used for northern analysis. (PDF)

S5 Table Oligomers used for primer extension analysis. (PDF)

## References

[1] Phizicky EM, Hopper AK. tRNA biology charges to the front. Genes Dev. 2010;24(17):1832–60. doi: 10.1101/gad.1956510 20810645 PMC2932967

[2] Boccaletto P, Machnicka MA, Purta E, Piatkowski P, Baginski B, Wirecki TK, et al. MODOMICS: a database of RNA modification pathways. 2017 update. Nucleic Acids Res. 2018;46(D1):D303–D7. doi: 10.1093/nar/gkx1030 29106616 PMC5753262

[3] Hopper AK. Transfer RNA post-transcriptional processing, turnover, and subcellular dynamics in the yeast Saccharomyces cerevisiae. Genetics. 2013;194(1):43–67. doi: 10.1534/genetics.112.147470 23633143 PMC3632480

[4] Ramos J, Fu D. The emerging impact of tRNA modifications in the brain and nervous system. Biochim. Biophys. Acta Gene Regul. Mech. 2019;1862(3):412–28. doi: 10.1016/j.bbagrm.2018.11.007 30529455

[5] Suzuki T. The expanding world of tRNA modifications and their disease relevance. Nat Rev Mol. Cell. Biol. 2021;22(6):375–92. doi: 10.1038/s41580-021-00342-0 33658722

[6] Muramatsu T, Nishikawa K, Nemoto F, Kuchino Y, Nishimura S, Miyazawa T, et al. Codon and amino-acid specificities of a transfer RNA are both converted by a single post-transcriptional modification. Nature. 1988;336(6195):179–81. doi: 10.1038/336179a0 3054566

[7] Johansson MJ, Esberg A, Huang B, Bjork GR, Bystrom AS. Eukaryotic wobble uridine modifications promote a functionally redundant decoding system. Mol. Cell. Biol. 2008;28(10):3301–12. doi: 10.1128/MCB.01542-07 18332122 PMC2423140

[8] Nedialkova DD, Leidel SA. Optimization of Codon Translation Rates via tRNA Modifications Maintains Proteome Integrity. Cell. 2015;161(7):1606–18. doi: 10.1016/j.cell.2015.05.022 26052047 PMC4503807

[9] Urbonavicius J, Qian O, Durand JMB, Hagervall TG, Bjork GR. Improvement of reading frame maintenance is a common function for several tRNA modifications. EMBO J. 2001;20(17):4863–73. doi: 10.1093/emboj/20.17.4863 11532950 PMC125605

[10] El Yacoubi B, Hatin I, Deutsch C, Kahveci T, Rousset JP, Iwata-Reuyl D, et al. A role for the universal Kae1/Qri7/YgjD (COG0533) family in tRNA modification. EMBO J. 2011;30(5):882–93. doi: 10.1038/emboj.2010.363 21285948 PMC3049207

[11] Putz J, Florentz C, Benseler F, Giege R. A single methyl group prevents the mischarging of a tRNA. Nat. Struct. Biol. 1994;1(9):580–2. doi: 10.1038/nsb0994-580 7634096

[12] Helm M, Giege R, Florentz C. A Watson-Crick base-pair-disrupting methyl group (m1A9) is sufficient for cloverleaf folding of human mitochondrial tRNALys. Biochemistry. 1999;38(40):13338–46. doi: 10.1021/bi991061g 10529209

[13] Kadaba S, Krueger A, Trice T, Krecic AM, Hinnebusch AG, Anderson J. Nuclear surveillance and degradation of hypomodified initiator tRNAMet in S. cerevisiae. Genes Dev. 2004;18(11):1227–40. doi: 10.1101/gad.1183804 15145828 PMC420349

[14] Alexandrov A, Chernyakov I, Gu W, Hiley SL, Hughes TR, Grayhack EJ, et al. Rapid tRNA decay can result from lack of nonessential modifications. Mol. Cell. 2006;21(1):87–96. doi: 10.1016/j.molcel.2005.10.036 16387656

[15] De Zoysa T, Phizicky EM. Hypomodified tRNA in evolutionarily distant yeasts can trigger rapid tRNA decay to activate the general amino acid control response, but with different consequences. PLoS Genet. 2020;16(8):e1008893. doi: 10.1371/journal.pgen.1008893 32841241 PMC7473580

[16] Chernyakov I, Whipple JM, Kotelawala L, Grayhack EJ, Phizicky EM. Degradation of several hypomodified mature tRNA species in Saccharomyces cerevisiae is mediated by Met22 and the 5’-3’ exonucleases Rat1 and Xrn1. Genes Dev. 2008;22(10):1369–80. doi: 10.1101/gad.1654308 18443146 PMC2377191

[17] Dichtl B, Stevens A, Tollervey D. Lithium toxicity in yeast is due to the inhibition of RNA processing enzymes. EMBO J. 1997;16(23):7184–95. doi: 10.1093/emboj/16.23.7184 9384595 PMC1170319

[18] Yun JS, Yoon JH, Choi YJ, Son YJ, Kim S, Tong L, et al. Molecular mechanism for the inhibition of DXO by adenosine 3’,5’-bisphosphate. Biochem. Biophys. Res. Commun. 2018;504(1):89–95. doi: 10.1016/j.bbrc.2018.08.135 30180947 PMC6145842

[19] Kotelawala L, Grayhack EJ, Phizicky EM. Identification of yeast tRNA Um(44) 2’-O-methyltransferase (Trm44) and demonstration of a Trm44 role in sustaining levels of specific tRNA(Ser) species. RNA. 2008;14(1):158–69. doi: 10.1261/rna.811008 18025252 PMC2151035

[20] Dewe JM, Whipple JM, Chernyakov I, Jaramillo LN, Phizicky EM. The yeast rapid tRNA decay pathway competes with elongation factor 1A for substrate tRNAs and acts on tRNAs lacking one or more of several modifications. RNA. 2012;18(10):1886–96. doi: 10.1261/rna.033654.112 22895820 PMC3446711

[21] Anderson J, Phan L, Cuesta R, Carlson BA, Pak M, Asano K, et al. The essential Gcd10p-Gcd14p nuclear complex is required for 1-methyladenosine modification and maturation of initiator methionyl-tRNA. Genes Dev. 1998;12(23):3650–62. doi: 10.1101/gad.12.23.3650 9851972 PMC317256

[22] Kadaba S, Wang X, Anderson JT. Nuclear RNA surveillance in Saccharomyces cerevisiae: Trf4p-dependent polyadenylation of nascent hypomethylated tRNA and an aberrant form of 5S rRNA. RNA. 2006;12(3):508–21. doi: 10.1261/rna.2305406 16431988 PMC1383588

[23] Wang X, Jia H, Jankowsky E, Anderson JT. Degradation of hypomodified tRNA(iMet) in vivo involves RNA-dependent ATPase activity of the DExH helicase Mtr4p. RNA. 2008;14(1):107–16. doi: 10.1261/rna.808608 18000032 PMC2151029

[24] LaCava J, Houseley J, Saveanu C, Petfalski E, Thompson E, Jacquier A, et al. RNA degradation by the exosome is promoted by a nuclear polyadenylation complex. Cell. 2005;121(5):713–24. doi: 10.1016/j.cell.2005.04.029 15935758

[25] Vanacova S, Wolf J, Martin G, Blank D, Dettwiler S, Friedlein A, et al. A new yeast poly(A) polymerase complex involved in RNA quality control. PLoS Biol. 2005;3(6):e189. doi: 10.1371/journal.pbio.0030189 15828860 PMC1079787

[26] Basavappa R, Sigler PB. The 3 A crystal structure of yeast initiator tRNA: functional implications in initiator/elongator discrimination. EMBO J. 1991;10(10):3105–11. doi: 10.1002/j.1460-2075.1991.tb07864.x 1915284 PMC453028

[27] Gudipati RK, Xu Z, Lebreton A, Seraphin B, Steinmetz LM, Jacquier A, et al. Extensive degradation of RNA precursors by the exosome in wild-type cells. Mol. Cell. 2012;48(3):409–21. doi: 10.1016/j.molcel.2012.08.018 23000176 PMC3496076

[28] Sonenberg N, Hinnebusch AG. Regulation of translation initiation in eukaryotes: mechanisms and biological targets. Cell. 2009;136(4):731–45. doi: 10.1016/j.cell.2009.01.042 19239892 PMC3610329

[29] Aitken CE, Lorsch JR. A mechanistic overview of translation initiation in eukaryotes. Nat. Struct. Mol. Biol. 2012;19(6):568–76. doi: 10.1038/nsmb.2303 22664984

[30] Sasikumar AN, Perez WB, Kinzy TG. The many roles of the eukaryotic elongation factor 1 complex. Wiley Interdiscip Rev RNA. 2012;3(4):543–55. doi: 10.1002/wrna.1118 22555874 PMC3374885

[31] Hinnebusch AG. Translational regulation of GCN4 and the general amino acid control of yeast. Annu. Rev. Microbiol. 2005;59:407–50. doi: 10.1146/annurev.micro.59.031805.133833 16153175

[32] Dever TE, Yang W, Astrom S, Bystrom AS, Hinnebusch AG. Modulation of tRNA(iMet), eIF-2, and eIF-2B expression shows that GCN4 translation is inversely coupled to the level of eIF-2.GTP.Met-tRNA(iMet) ternary complexes. Mol. Cell.Biol. 1995;15(11):6351–63. doi: 10.1128/MCB.15.11.6351 7565788 PMC230887

[33] Pavon-Eternod M, Gomes S, Rosner MR, Pan T. Overexpression of initiator methionine tRNA leads to global reprogramming of tRNA expression and increased proliferation in human epithelial cells. RNA. 2013;19(4):461–6. doi: 10.1261/rna.037507.112 23431330 PMC3677255

[34] Birch J, Clarke CJ, Campbell AD, Campbell K, Mitchell L, Liko D, et al. The initiator methionine tRNA drives cell migration and invasion leading to increased metastatic potential in melanoma. Biol Open. 2016;5(10):1371–9. doi: 10.1242/bio.019075 27543055 PMC5087684

[35] Watanabe K, Miyagawa R, Tomikawa C, Mizuno R, Takahashi A, Hori H, et al. Degradation of initiator tRNAMet by Xrn1/2 via its accumulation in the nucleus of heat-treated HeLa cells. Nucleic Acids Res. 2013;41(8):4671–85. doi: 10.1093/nar/gkt153 23471000 PMC3632136

[36] Liu F, Clark W, Luo G, Wang X, Fu Y, Wei J, et al. ALKBH1-Mediated tRNA Demethylation Regulates Translation. Cell. 2016;167(3):816–28 e16. doi: 10.1016/j.cell.2016.09.038 27745969 PMC5119773

[37] Macari F, El-Houfi Y, Boldina G, Xu H, Khoury-Hanna S, Ollier J, et al. TRM6/61 connects PKCalpha with translational control through tRNAi(Met) stabilization: impact on tumorigenesis. Oncogene. 2016;35(14):1785–96. doi: 10.1038/onc.2015.244 26234676

[38] Tang J, Jia P, Xin P, Chu J, Shi DQ, Yang WC. The Arabidopsis TRM61/TRM6 complex is a bona fide tRNA N1-methyladenosine methyltransferase. J. Exp. Bot. 2020;71(10):3024–36. doi: 10.1093/jxb/eraa100 32095811 PMC7475180

[39] Parfrey LW, Lahr DJ, Knoll AH, Katz LA. Estimating the timing of early eukaryotic diversification with multigene molecular clocks. Proc Natl Acad Sci U S A. 2011;108(33):13624–9. doi: 10.1073/pnas.1110633108 21810989 PMC3158185

[40] Kim DU, Hayles J, Kim D, Wood V, Park HO, Won M, et al. Analysis of a genome-wide set of gene deletions in the fission yeast Schizosaccharomyces pombe. Nat. Biotechnol. 2010;28(6):617–23. doi: 10.1038/nbt.1628 20473289 PMC3962850

[41] Anderson J, Phan L, Hinnebusch AG. The Gcd10p/Gcd14p complex is the essential two-subunit tRNA(1- methyladenosine) methyltransferase of Saccharomyces cerevisiae. Proc. Natl. Acad. Sci. U. S. A. 2000;97(10):5173–8. doi: 10.1073/pnas.090102597 10779558 PMC25801

[42] Mao J, Schmidt O, Soll D. Dimeric transfer RNA precursors in S. pombe. Cell. 1980;21(2):509–16. doi: 10.1016/0092-8674(80)90488-2 7407924

[43] Seufert W, Jentsch S. Nucleotide sequence of two tRNA(Arg)-tRNA(Asp) tandem genes linked to duplicated UBC genes in Saccharomyces cerevisiae. Nucleic Acids Res. 1990;18(6):1638. doi: 10.1093/nar/18.6.1638 2183198 PMC330544

[44] Schmidt O, Mao J, Ogden R, Beckmann J, Sakano H, Abelson J, et al. Dimeric tRNA precursors in yeast. Nature. 1980;287(5784):750–2. doi: 10.1038/287750a0 6253814

[45] Miyamoto R, Sugiura R, Kamitani S, Yada T, Lu Y, Sio SO, et al. Tol1, a fission yeast phosphomonoesterase, is an in vivo target of lithium, and its deletion leads to sulfite auxotrophy. J Bacteriol. 2000;182(13):3619–25. doi: 10.1128/JB.182.13.3619-3625.2000 10850973 PMC94529

[46] Houseley J, Tollervey D. The nuclear RNA surveillance machinery: The link between ncRNAs and genome structure in budding yeast? Biochim. Biophys. Acta. 2008;1779(4):239–46. doi: 10.1016/j.bbagrm.2007.12.008 18211833

[47] Schneider C, Anderson JT, Tollervey D. The exosome subunit Rrp44 plays a direct role in RNA substrate recognition. Mol. Cell. 2007;27(2):324–31. doi: 10.1016/j.molcel.2007.06.006 17643380 PMC7610968

[48] Win TZ, Draper S, Read RL, Pearce J, Norbury CJ, Wang SW. Requirement of fission yeast Cid14 in polyadenylation of rRNAs. Mol. Cell. Biol. 2006;26(5):1710–21. doi: 10.1128/MCB.26.5.1710-1721.2006 16478992 PMC1430263

[49] Buhler M, Moazed D. Transcription and RNAi in heterochromatic gene silencing. Nat. Struct. Mol. Biol. 2007;14(11):1041–8. doi: 10.1038/nsmb1315 17984966

[50] Keller C, Woolcock K, Hess D, Buhler M. Proteomic and functional analysis of the noncanonical poly(A) polymerase Cid14. RNA. 2010;16(6):1124–9. doi: 10.1261/rna.2053710 20403971 PMC2874164

[51] Hu L, Yao F, Ma Y, Liu Q, Chen S, Hayafuji T, et al. Genetic evidence for involvement of membrane trafficking in the action of 5-fluorouracil. Fungal Genet Biol. 2016;93:17–24. doi: 10.1016/j.fgb.2016.05.007 27255861

[52] Amberg DC, Goldstein AL, Cole CN. Isolation and characterization of RAT1: an essential gene of Saccharomyces cerevisiae required for the efficient nucleocytoplasmic trafficking of mRNA. Genes Dev. 1992;6(7):1173–89. doi: 10.1101/gad.6.7.1173 1628825

[53] Kolitz SE, Lorsch JR. Eukaryotic initiator tRNA: finely tuned and ready for action. FEBS Lett. 2010;584(2):396–404. doi: 10.1016/j.febslet.2009.11.047 19925799 PMC2795131

[54] Ozanick SG, Bujnicki JM, Sem DS, Anderson JT. Conserved amino acids in each subunit of the heteroligomeric tRNA m1A58 Mtase from Saccharomyces cerevisiae contribute to tRNA binding. Nucleic Acids Res. 2007;35(20):6808–19. doi: 10.1093/nar/gkm574 17932071 PMC2175304

[55] Nagarajan VK, Jones CI, Newbury SF, Green PJ. XRN 5’—>3’ exoribonucleases: structure, mechanisms and functions. Biochim. Biophys. Acta. 2013;1829(6–7):590–603. doi: 10.1016/j.bbagrm.2013.03.005 23517755 PMC3742305

[56] Hudson BH, York JD. Roles for nucleotide phosphatases in sulfate assimilation and skeletal disease. Adv. Biol. Regul. 2012;52(1):229–38. doi: 10.1016/j.advenzreg.2011.11.002 22100882 PMC3845023

[57] Lin S, Liu Q, Lelyveld VS, Choe J, Szostak JW, Gregory RI. Mettl1/Wdr4-Mediated m(7)G tRNA Methylome Is Required for Normal mRNA Translation and Embryonic Stem Cell Self-Renewal and Differentiation. Mol. Cell. 2018;71(2):244–55 e5. doi: 10.1016/j.molcel.2018.06.001 29983320 PMC6086580

[58] Orellana EA, Liu Q, Yankova E, Pirouz M, De Braekeleer E, Zhang W, et al. METTL1-mediated m(7)G modification of Arg-TCT tRNA drives oncogenic transformation. Mol Cell. 2021;81(16):3323–38 e14. doi: 10.1016/j.molcel.2021.06.031 34352207 PMC8380730

[59] Tuorto F, Liebers R, Musch T, Schaefer M, Hofmann S, Kellner S, et al. RNA cytosine methylation by Dnmt2 and NSun2 promotes tRNA stability and protein synthesis. Nat Struct Mol Biol. 2012;19(9):900–5. doi: 10.1038/nsmb.2357 22885326

[60] Huang Y, Bayfield MA, Intine RV, Maraia RJ. Separate RNA-binding surfaces on the multifunctional La protein mediate distinguishable activities in tRNA maturation. Nat Struct Mol Biol. 2006;13(7):611–8. doi: 10.1038/nsmb1110 16799560

[61] Weick EM, Puno MR, Januszyk K, Zinder JC, DiMattia MA, Lima CD. Helicase-Dependent RNA Decay Illuminated by a Cryo-EM Structure of a Human Nuclear RNA Exosome-MTR4 Complex. Cell. 2018;173(7):1663–77 e21. doi: 10.1016/j.cell.2018.05.041 29906447 PMC6124691

[62] Guy MP, Young DL, Payea MJ, Zhang X, Kon Y, Dean KM, et al. Identification of the determinants of tRNA function and susceptibility to rapid tRNA decay by high-throughput in vivo analysis. Genes Dev. 2014;28(15):1721–32. doi: 10.1101/gad.245936.114 25085423 PMC4117946

[63] Bystrom AS, Fink GR. A functional analysis of the repeated methionine initiator tRNA genes (IMT) in yeast. Mol. Genet. Genet. 1989;216(2–3):276–86. doi: 10.1007/BF00334366 2664453

[64] von Pawel-Rammingen U, Astrom S, Bystrom AS. Mutational analysis of conserved positions potentially important for initiator tRNA function in Saccharomyces cerevisiae. Mol. Cell. Biol. 1992;12(4):1432–42. doi: 10.1128/mcb.12.4.1432-1442.1992 1549105 PMC369584

[65] Johnson AW. Rat1p and Xrn1p are functionally interchangeable exoribonucleases that are restricted to and required in the nucleus and cytoplasm, respectively. Mol. Cell. Biol. 1997;17(10):6122–30. doi: 10.1128/MCB.17.10.6122 9315672 PMC232462

[66] Kramer EB, Hopper AK. Retrograde transfer RNA nuclear import provides a new level of tRNA quality control in Saccharomyces cerevisiae. Proc. Natl. Acad. Sci. U. S. A. 2013;110(52):21042–7. doi: 10.1073/pnas.1316579110 24297920 PMC3876269

[67] Shelton VM, Sosnick TR, Pan T. Altering the intermediate in the equilibrium folding of unmodified yeast tRNAPhe with monovalent and divalent cations. Biochemistry. 2001;40(12):3629–38. doi: 10.1021/bi002646+ 11297430

[68] Whipple JM, Lane EA, Chernyakov I, D’Silva S, Phizicky EM. The yeast rapid tRNA decay pathway primarily monitors the structural integrity of the acceptor and T-stems of mature tRNA. Genes Dev. 2011;25(11):1173–84. doi: 10.1101/gad.2050711 21632824 PMC3110955

[69] Bahler J, Wu JQ, Longtine MS, Shah NG, McKenzie A 3rd, Steever AB, et al. Heterologous modules for efficient and versatile PCR-based gene targeting in Schizosaccharomyces pombe. Yeast. 1998;14(10):943–51. doi: 10.1002/(SICI)1097-0061(199807)14:10&lt;943::AID-YEA292&gt;3.0.CO;2-Y 9717240

[70] Gibson DG, Young L, Chuang RY, Venter JC, Hutchison CA 3rd, Smith HO. Enzymatic assembly of DNA molecules up to several hundred kilobases. Nat Methods. 2009;6(5):343–5. doi: 10.1038/nmeth.1318 19363495

[71] Vjestica A, Marek M, Nkosi PJ, Merlini L, Liu G, Berard M, et al. A toolbox of stable integration vectors in the fission yeast Schizosaccharomyces pombe. J Cell Sci. 2020;133(1). doi: 10.1242/jcs.240754 31801797

[72] Houston L, Platten EM, Connelly SM, Wang J, Grayhack EJ. Frameshifting at collided ribosomes is modulated by elongation factor eEF3 and by integrated stress response regulators Gcn1 and Gcn20. RNA. 2022;28(3):320–39. doi: 10.1261/rna.078964.121 34916334 PMC8848926

[73] Elder RT, Loh EY, Davis RW. RNA from the yeast transposable element Ty1 has both ends in the direct repeats, a structure similar to retrovirus RNA. Proc. Natl. Acad. Sci. U. S. A. 1983;80(9):2432–6. doi: 10.1073/pnas.80.9.2432 6189122 PMC393839

[74] Jackman JE, Montange RK, Malik HS, Phizicky EM. Identification of the yeast gene encoding the tRNA m1G methyltransferase responsible for modification at position 9. RNA. 2003;9(5):574–85. doi: 10.1261/rna.5070303 12702816 PMC1370423

[75] Guy MP, Podyma BM, Preston MA, Shaheen HH, Krivos KL, Limbach PA, et al. Yeast Trm7 interacts with distinct proteins for critical modifications of the tRNAPhe anticodon loop. RNA. 2012;18(10):1921–33. doi: 10.1261/rna.035287.112 22912484 PMC3446714

[76] Payea MJ, Hauke AC, De Zoysa T, Phizicky EM. Mutations in the anticodon stem of tRNA cause accumulation and Met22-dependent decay of pre-tRNA in yeast. RNA. 2020;26(1):29–43. doi: 10.1261/rna.073155.119 31619505 PMC6913130

[77] Corpet F. Multiple sequence alignment with hierarchical clustering. Nucl. Acids Res. 1988;16:10881–90. doi: 10.1093/nar/16.22.10881 2849754 PMC338945

[78] Xiang S, Cooper-Morgan A, Jiao X, Kiledjian M, Manley JL, Tong L. Structure and function of the 5’—>3’ exoribonuclease Rat1 and its activating partner Rai1. Nature. 2009;458(7239):784–8. doi: 10.1038/nature07731 19194460 PMC2739979

[79] Albert A, Yenush L, Gil-Mascarell MR, Rodriguez PL, Patel S, Martinez-Ripoll M, et al. X-ray structure of yeast Hal2p, a major target of lithium and sodium toxicity, and identification of framework interactions determining cation sensitivity. J. Mol. Biol. 2000;295(4):927–38. doi: 10.1006/jmbi.1999.3408 10656801

[80] Forsburg SL. Comparison of Schizosaccharomyces pombe expression systems. Nucleic Acids Res. 1993;21(12):2955–6. doi: 10.1093/nar/21.12.2955 8332516 PMC309706

[81] Gelperin DM, White MA, Wilkinson ML, Kon Y, Kung LA, Wise KJ, et al. Biochemical and genetic analysis of the yeast proteome with a movable ORF collection. Genes Dev. 2005;19(23):2816–26. doi: 10.1101/gad.1362105 16322557 PMC1315389

