# Supplementary figures and images for: Initiator tRNA lacking 1-methyladenosine is targeted by the rapid tRNA decay pathway in evolutionarily distant yeast species
Source: PLoS Genet. 2022 Jul 28;18(7):e1010215. doi: 10.1371/journal.pgen.1010215 (PMC9362929; doi:10.1371/journal.pgen.1010215)

A

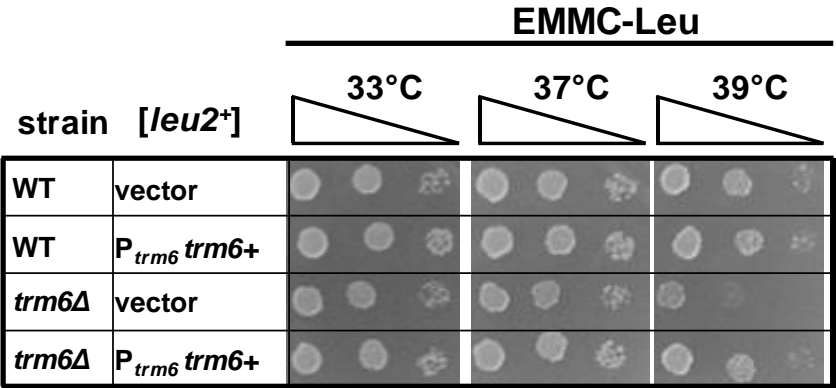

B

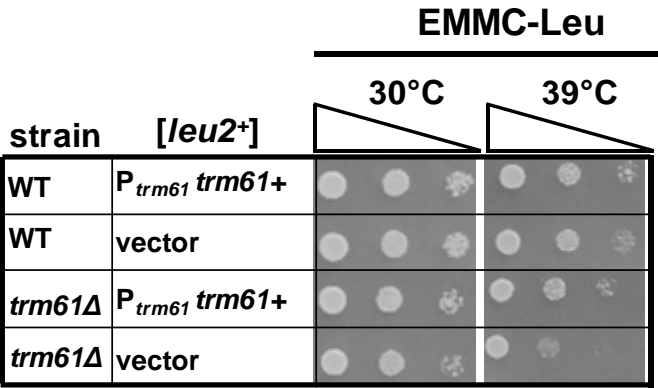

Supplement: S1 Fig — (A) The temperature sensitivity of an S. pombe trm6Δ mutant is complemented by [Ptrm6 trm6+ leu2+] on EMMC-leu media. WT and trm6Δ cells expressing Ptrm6 trm6+ were grown overnight in EMMC-leu media 30°C and analyzed for growth at the indicated temperatures. (B) The temperature sensitivity of S. pombe trm61Δ is complemented by [Ptrm61 trm61+ leu2+] on EMMC-leu media. WT and trm61Δ cells expressing Ptrm61 trm61+ were grown overnight in EMMC-leu media 30°C and analyzed for growth. (PDF) [file pgen.1010215.s001.pdf]

**A**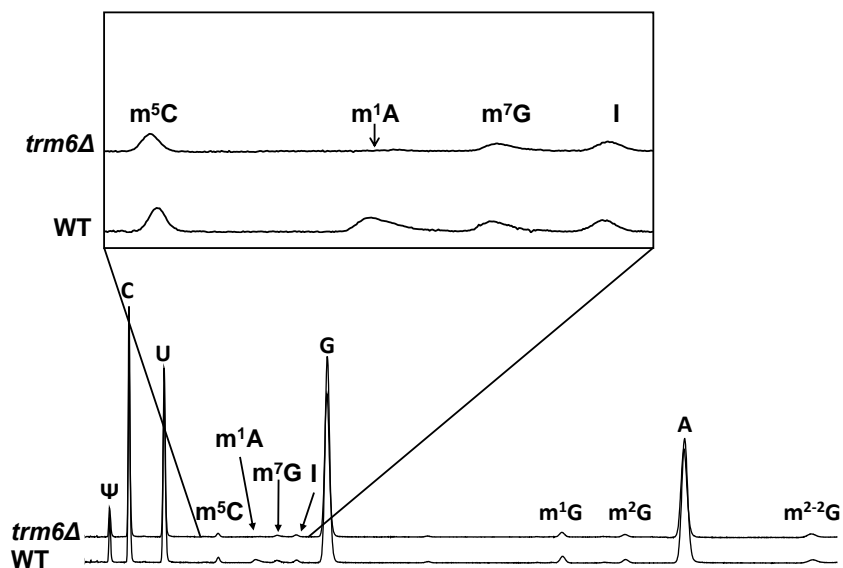**B**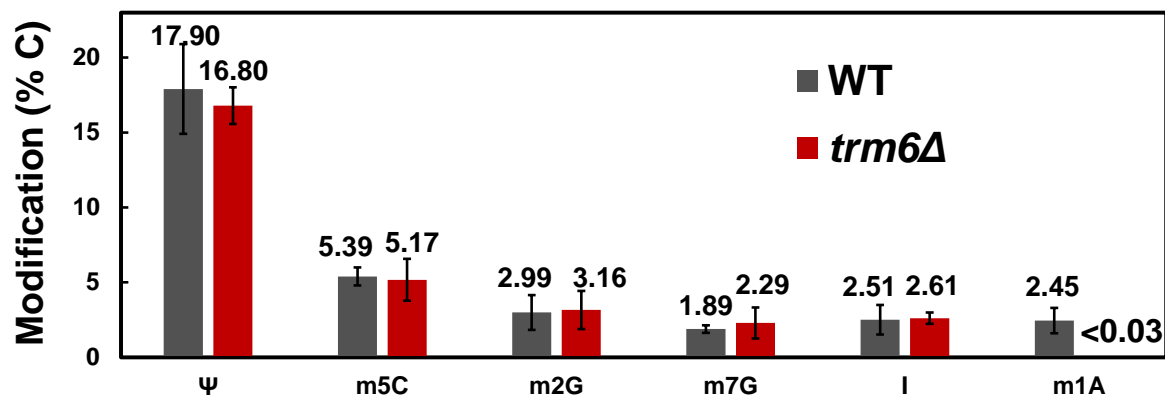**C**

| Modification | WT             | <i>trm6Δ</i>   |
|--------------|----------------|----------------|
| $m^1A$       | $2.5 \pm 0.8$  | $<0.03$        |
| $\psi$       | $17.9 \pm 3.0$ | $16.8 \pm 1.2$ |
| $m^5C$       | $5.4 \pm 0.6$  | $5.2 \pm 1.4$  |
| $m^2G$       | $3.0 \pm 1.2$  | $3.2 \pm 1.3$  |
| $m^7G$       | $1.9 \pm 0.3$  | $2.3 \pm 1.0$  |
| I            | $2.5 \pm 1.0$  | $2.6 \pm 0.4$  |

Supplement: S2 Fig — (A,B) Bulk tRNA from S. pombe trm6Δ mutants have no detectable m1A. S. pombe trm6Δ mutants and WT cells were grown in biological triplicate in YES media at 30°C and bulk tRNA was purified, digested to nucleosides, and analyzed for modifications by HPLC as described in Materials and Methods. (A) A trace of the A258 nm of eluted nucleosides of bulk tRNA. (B) Quantification of levels of modified nucleosides of purified tRNATyr(GUA). The bar chart depicts the average moles/mol of nucleosides (expressed as a percentage of the moles of cytidine), with associated standard deviation; WT, gray; S. pombe trm6Δ, red. The data is also tabulated in the table below (C). (PDF) [file pgen.1010215.s002.pdf]

**A**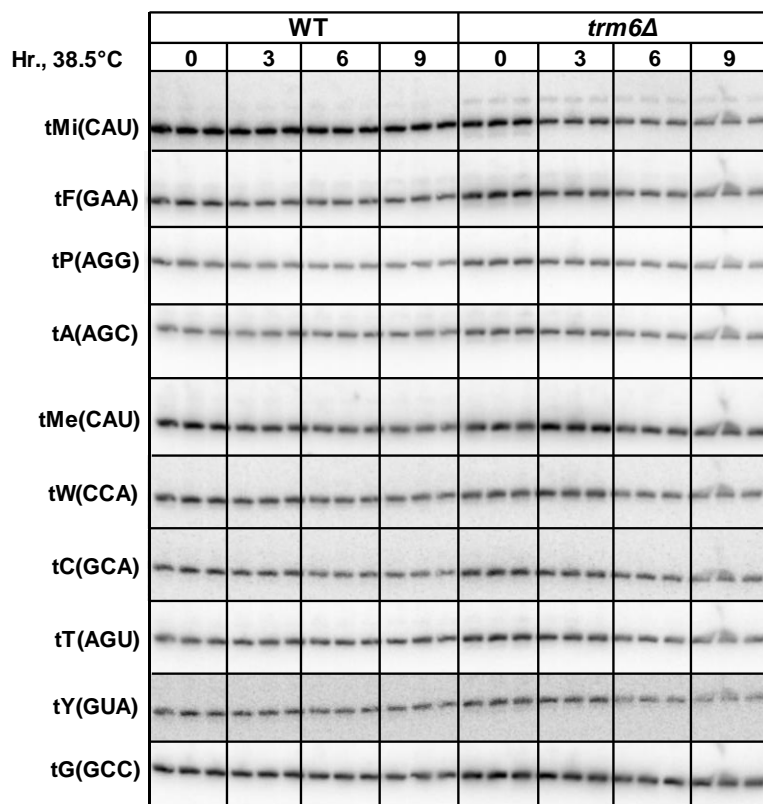**B***trm6Δ*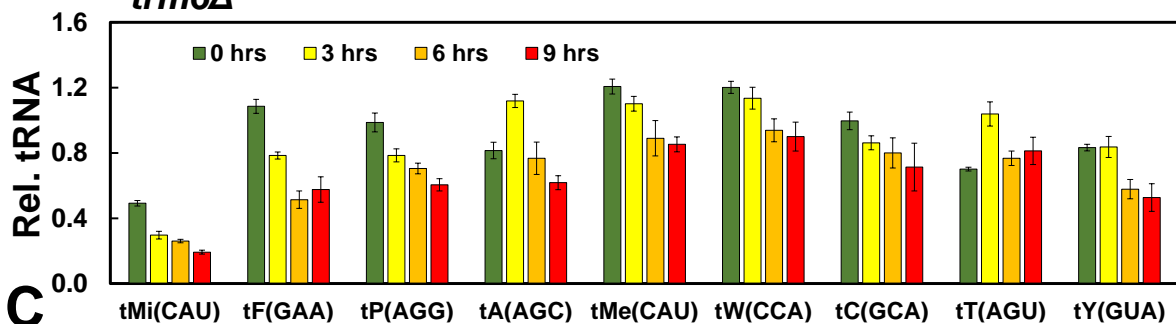**C**

WT

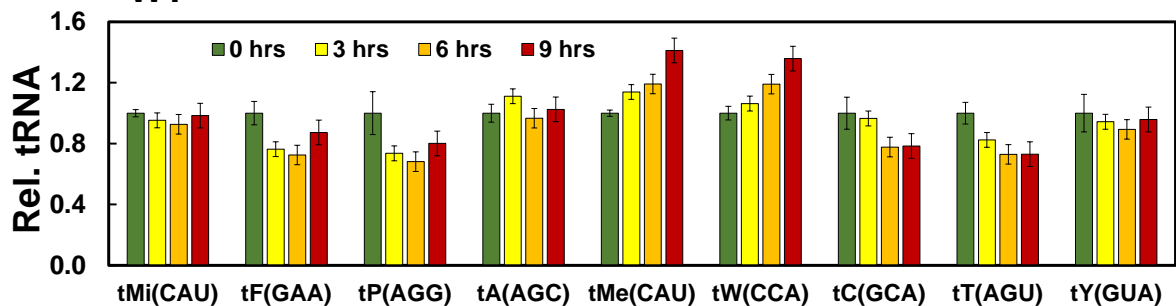

Supplement: S3 Fig — (A) Northern blot. Full analysis is shown of tRNAs analyzed in the northern blot shown in Fig 2A. (B,C) Quantification of tRNA levels. The bar chart depicts relative levels of tRNA species at each temperature, relative to their levels in WT at 30°C. (PDF) [file pgen.1010215.s003.pdf]

**A**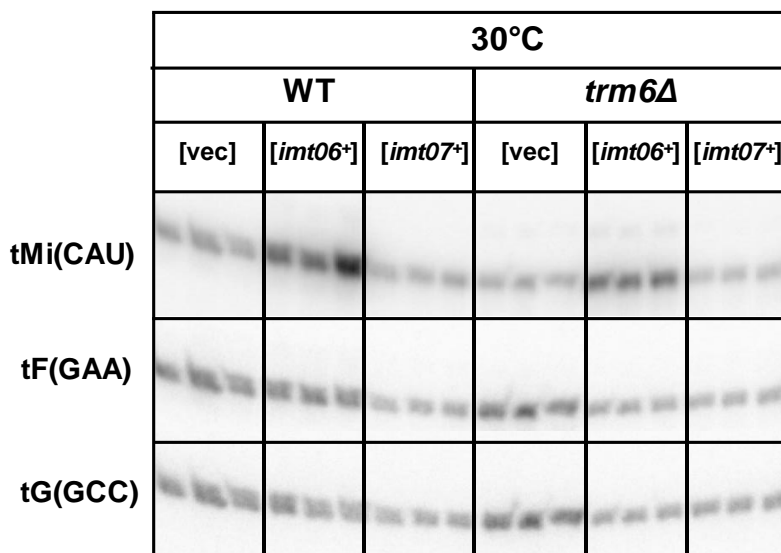**B**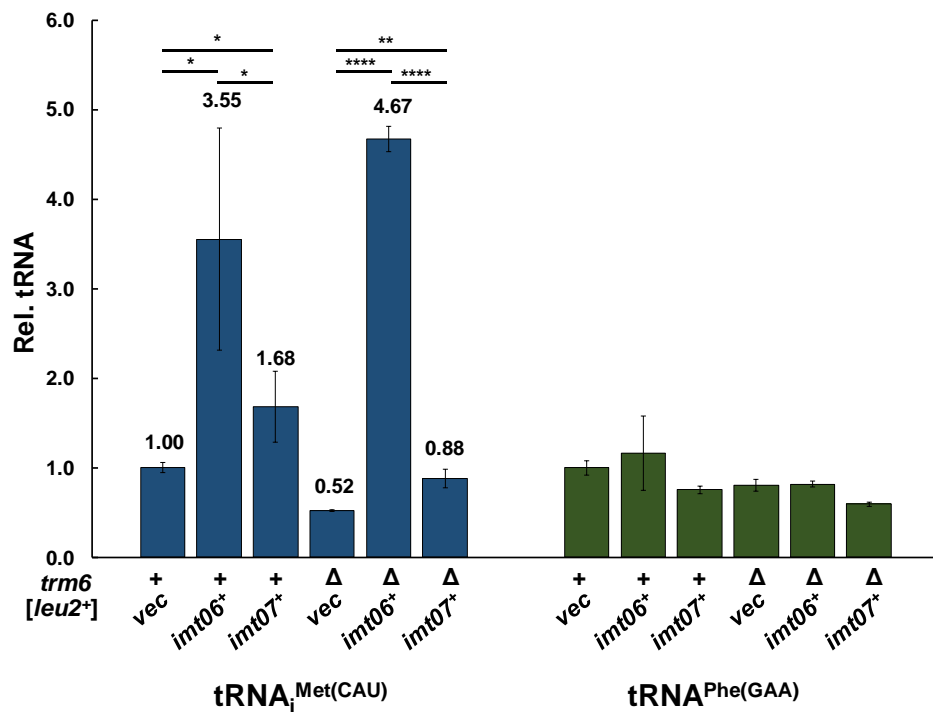

Supplement: S4 Fig — (A) Northern blot. Strains were grown and analyzed as in Fig 2D. (B) Quantification of tRNA levels. tRNA levels were quantified as in Fig 2B. (PDF) [file pgen.1010215.s004.pdf]

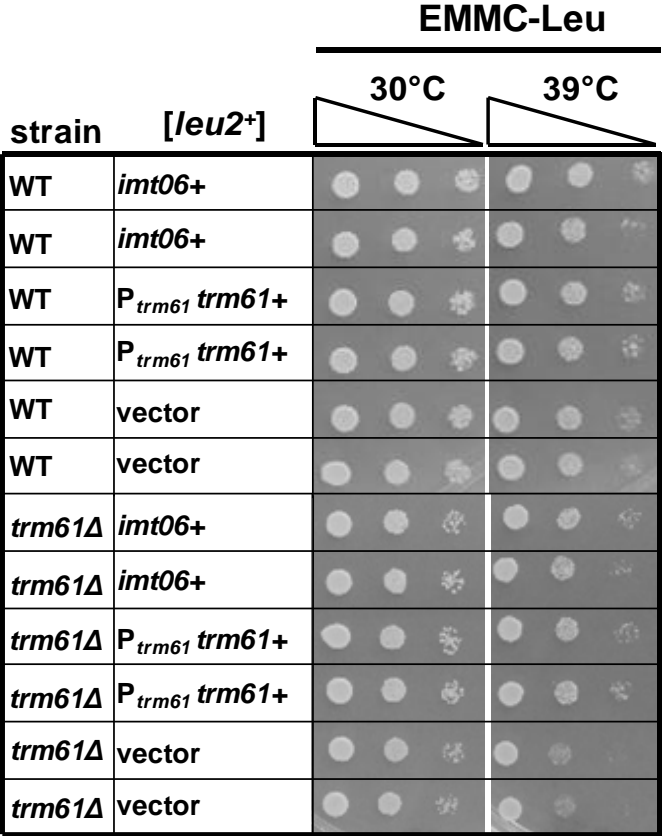

Supplement: S5 Fig — Strains with plasmids as indicated were grown overnight in EMMC-Leu media at 30°C and analyzed for growth as in Fig 1A on indicated plates and temperatures. (PDF) [file pgen.1010215.s005.pdf]

Fig S7

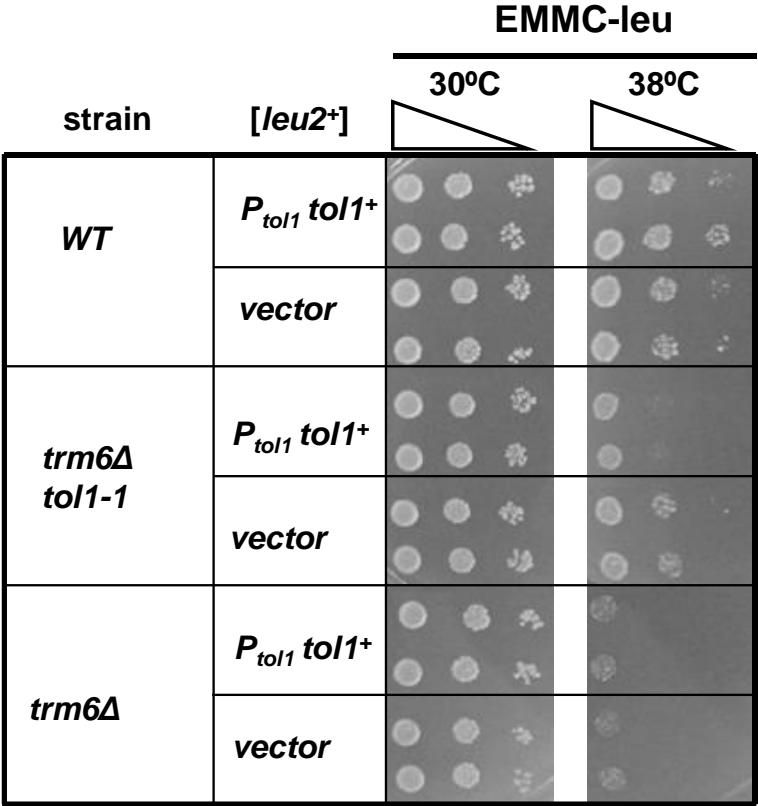

Supplement: S7 Fig — trm6Δ tol1-1 mutants, trm6Δ mutants and WT strains were transformed with either [Ptol1 tol1+ leu2+] or empty vector, grown in EMMC-leu, and spotted. (PDF) [file pgen.1010215.s007.pdf]

A

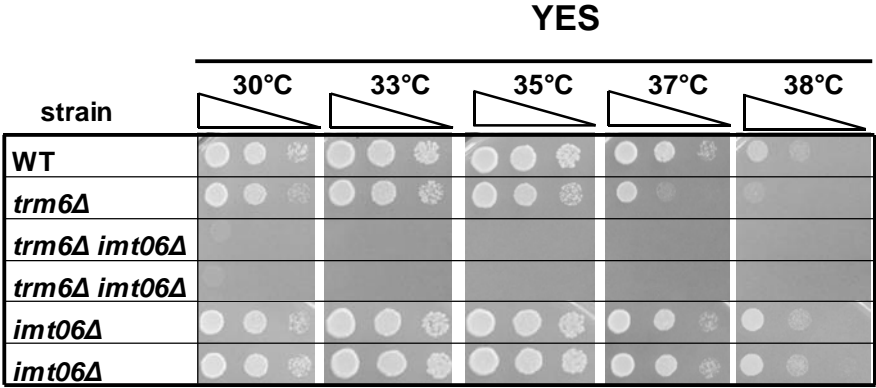

B

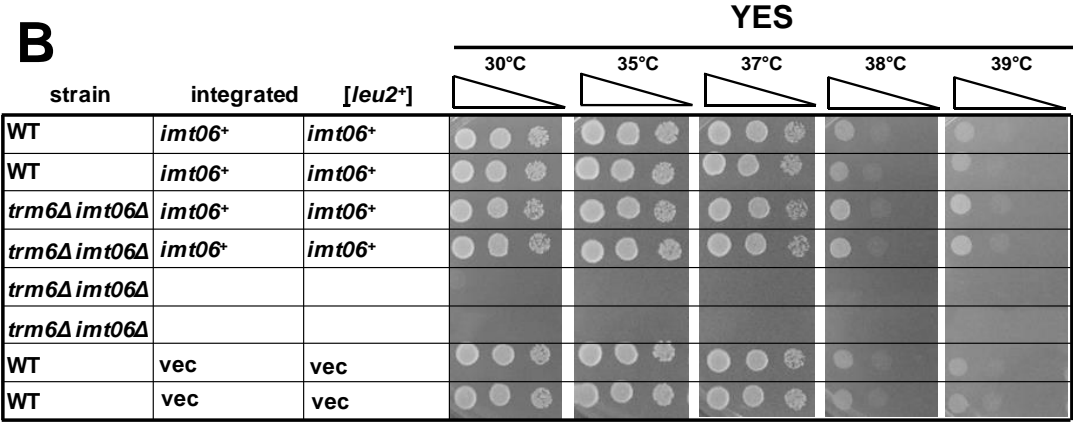

C

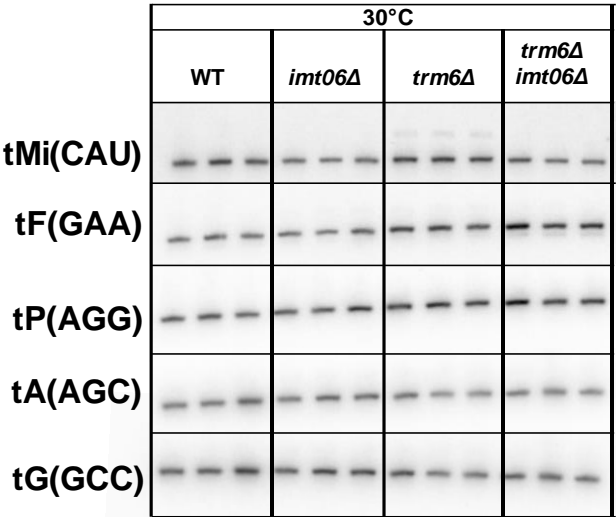

Supplement: S9 Fig — (A) Deletion of the imt06 gene encoding tRNAiMet(CAU) in an S. pombe trm6Δ mutant severely exacerbates its growth. Strains from the growth test in Fig 4A are shown after 2 days of growth (B) Complementation of trm6Δ imt06Δ growth defect with an integrated imt06 and a [leu2+ imt06+] plasmid. trm6Δ imt06Δ and WT cells expressing tRNAiMet(CAU) from a chromosomally integrated copy of imt06+ and from a [leu2+ imt06+] plasmid, and controls were grown overnight in EMMC or EMMC-leu media at 30°C, and analyzed for growth. (C) Levels of tRNAiMet(CAU) are significantly reduced in S. pombe trm6Δ imt06Δ mutants at 30°C. The Northern blot from Fig 4B is shown. (PDF) [file pgen.1010215.s009.pdf]

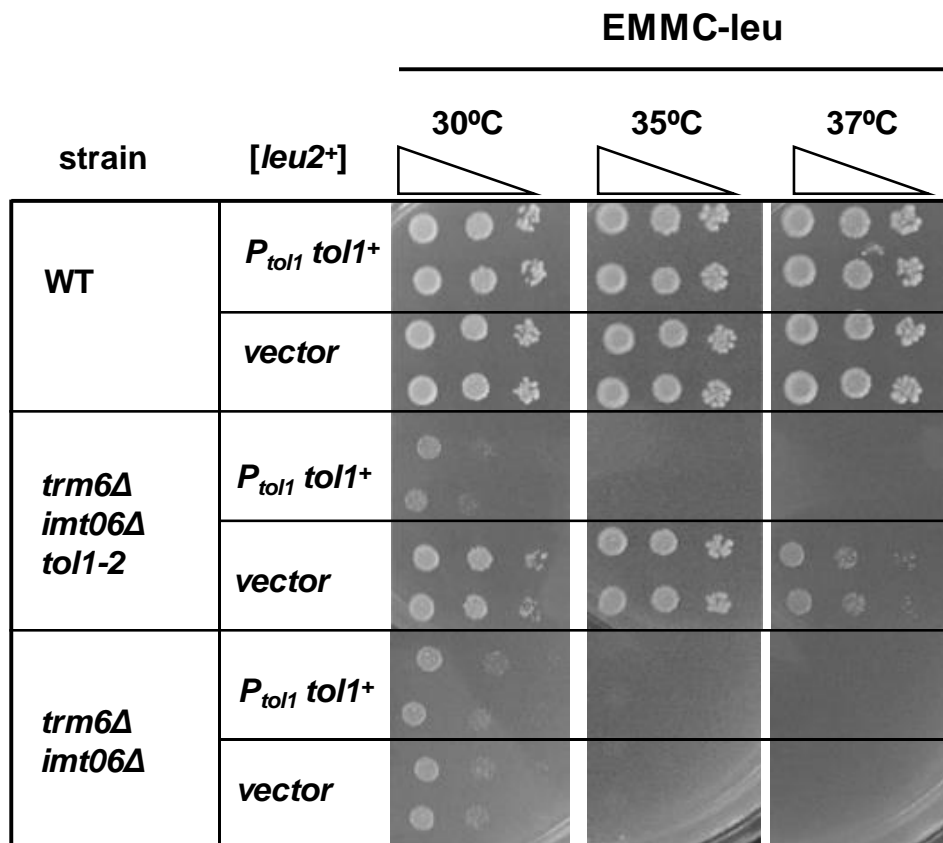

Supplement: S11 Fig — WT, trm6Δ imt06Δ, and trm6Δ imt06Δ tol1-2 cells expressing Ptol1 tol1+ or a vector [80] were grown overnight in EMMC-Leu media at 30°C, and analyzed for growth (PDF) [file pgen.1010215.s011.pdf]

A

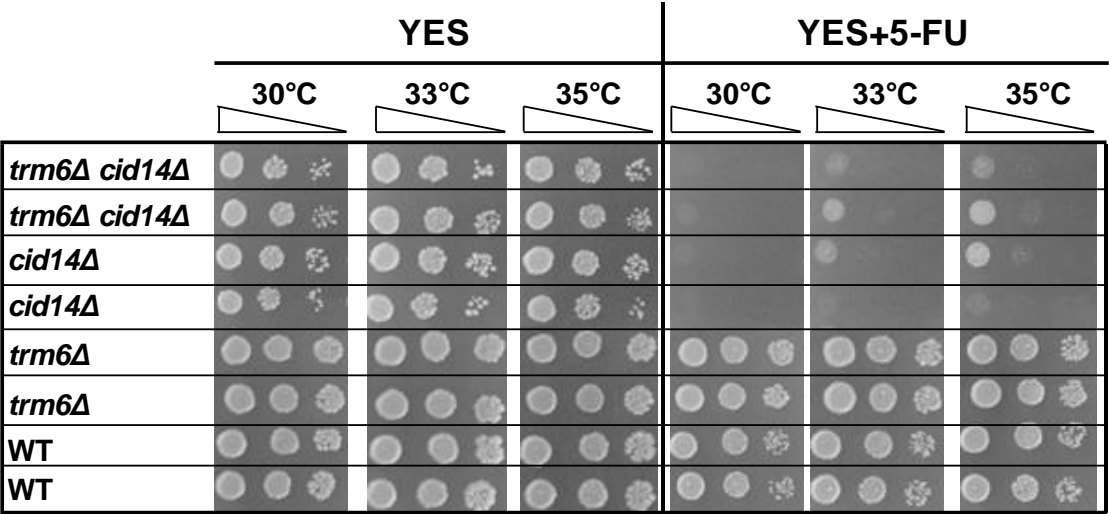

B

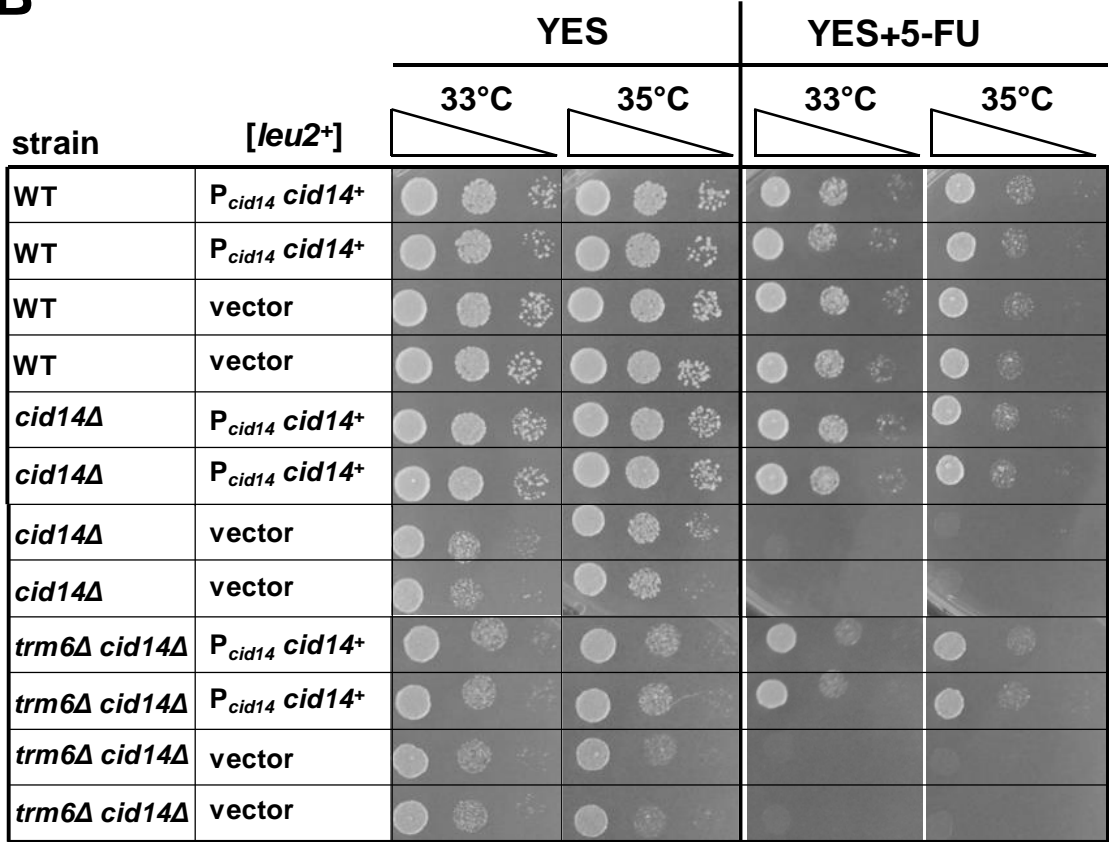

Supplement: S12 Fig — (A) Analysis of growth of cid14Δ strains on YES media with or without 5-FU. Strains were grown overnight in YES media at 30°C and analyzed for growth as in Fig 1A on indicated plates and temperatures. (B) Complementation of the 5-FU sensitivity of cid14Δ strains. Strains were grown overnight in EMMC-Leu media at 30°C and analyzed for growth on indicated plates and temperatures. (PDF) [file pgen.1010215.s012.pdf]

A

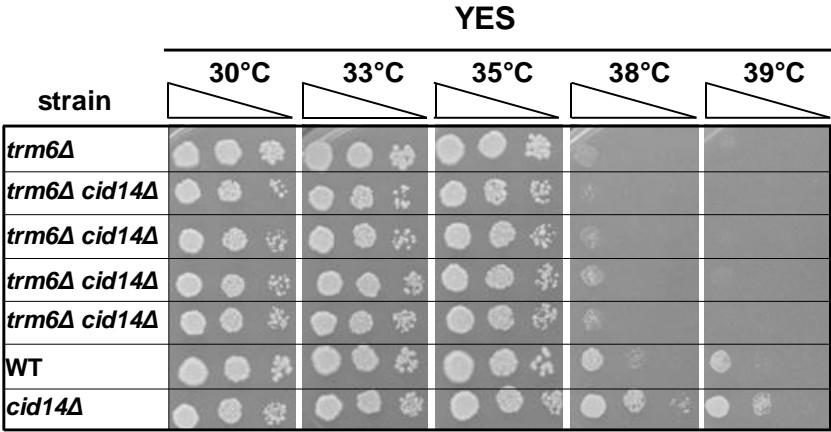

B

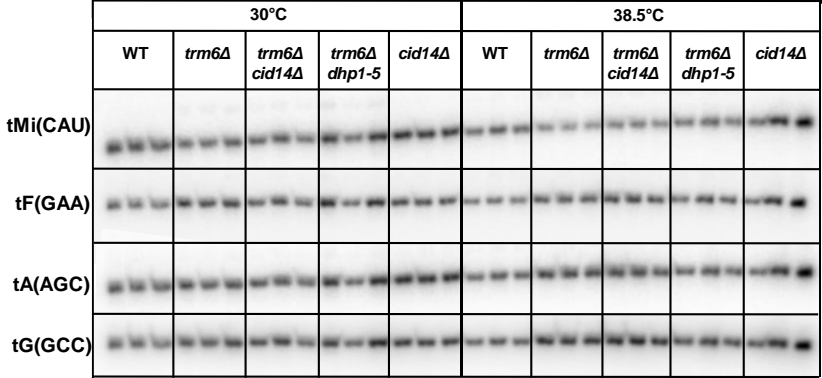

C

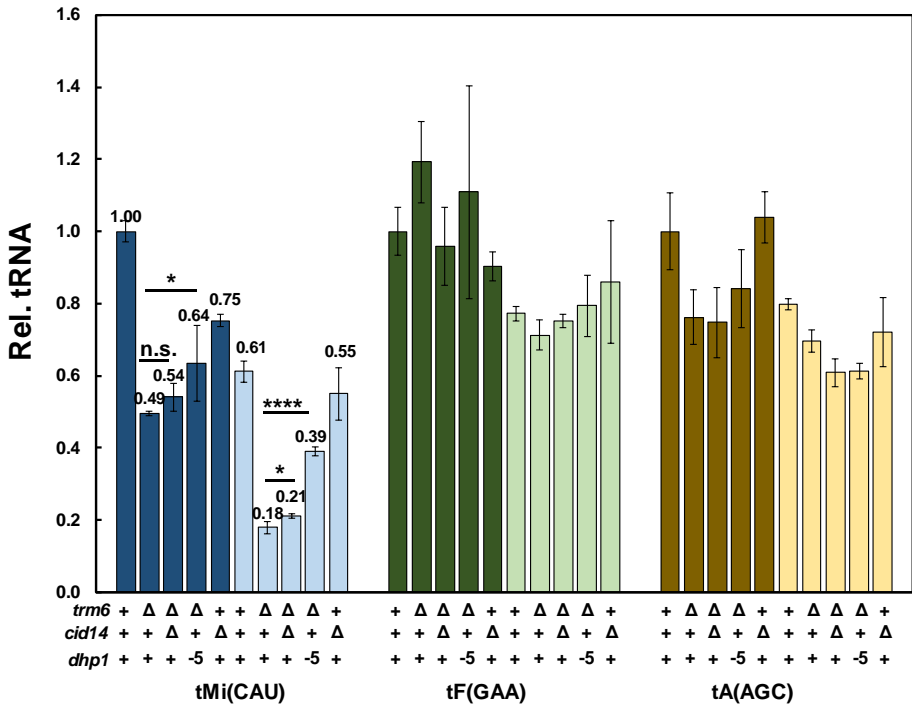

Supplement: S13 Fig — (A) A cid14Δ mutation does not suppress the growth defect of S. pombe trm6Δ mutants. Strains were grown overnight in YES media at 30°C and analyzed for growth as in Fig 1A on indicated plates and temperatures. (B,C) A cid14Δ mutation has only a minimal effect on tRNAiMet(CAU) levels in S. pombe trm6Δ mutants. (PDF) [file pgen.1010215.s013.pdf]

**A**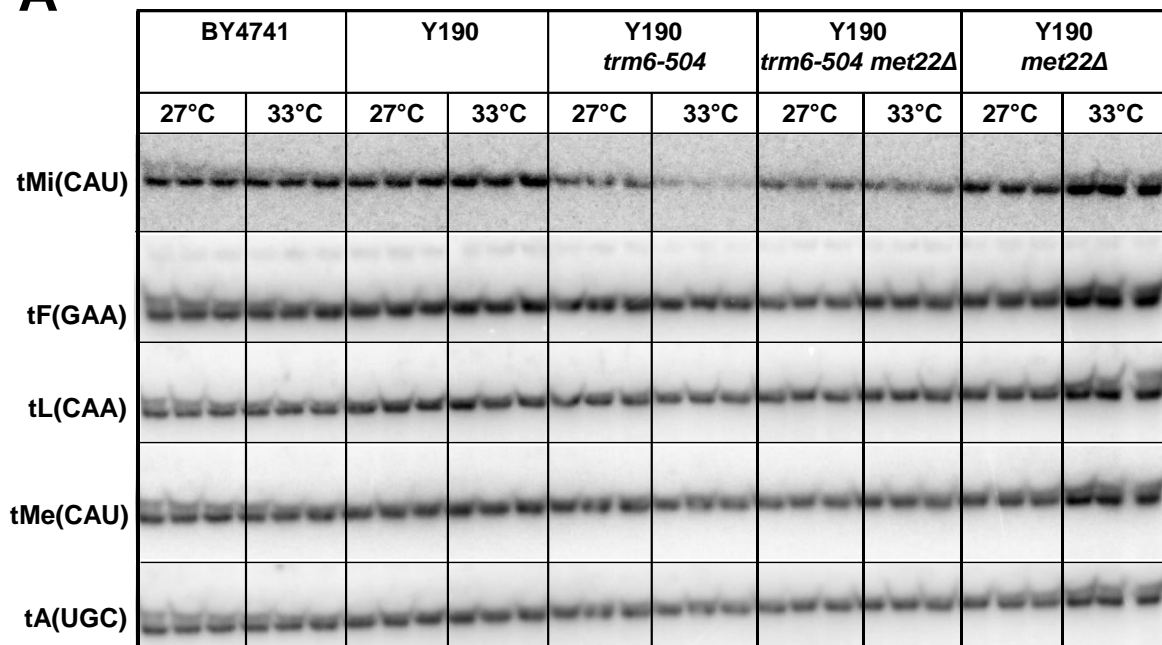**B**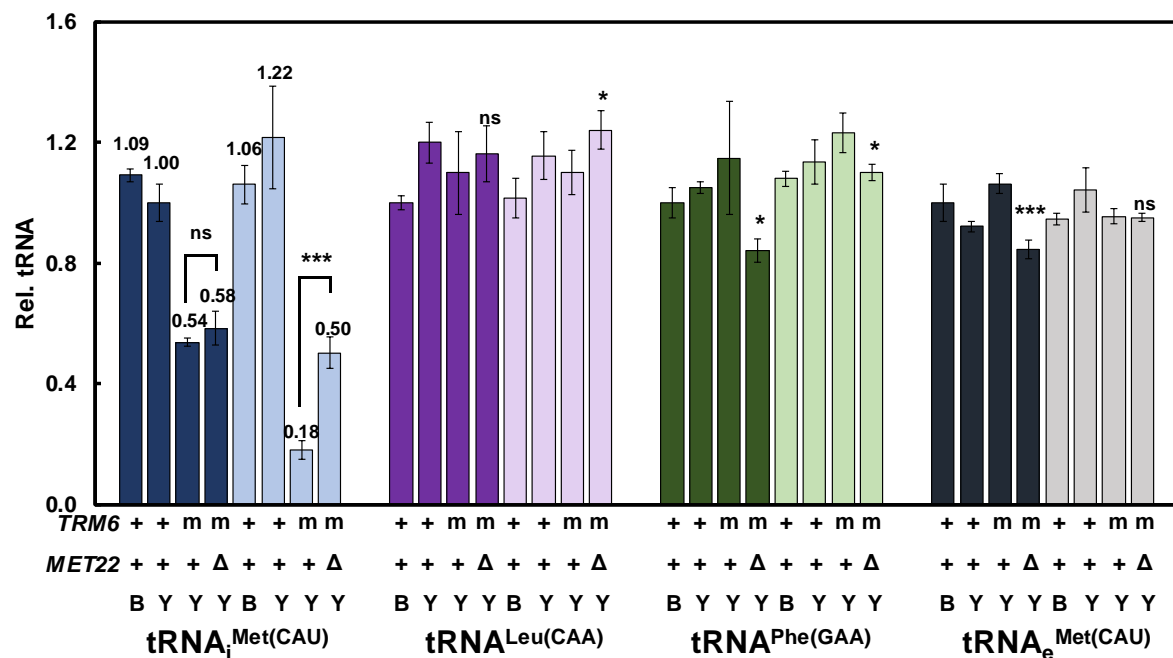

Supplement: S14 Fig — Strains were grown in YPD at 27°C and shifted to 33°C for 6 hours as described in Materials and Methods, and RNA was isolated and analyzed by northern blotting. (A) Northern Blot. (B) Quantification of northern. B; standard BY4741 WT strain background; Y, Y190 background of original trm6-504 mutant; m, trm6-504 mutant. (PDF) [file pgen.1010215.s014.pdf]

**A**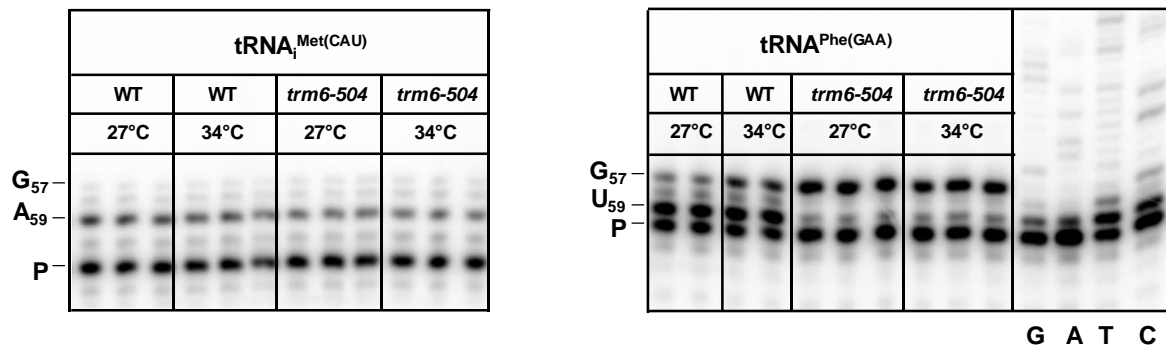**B**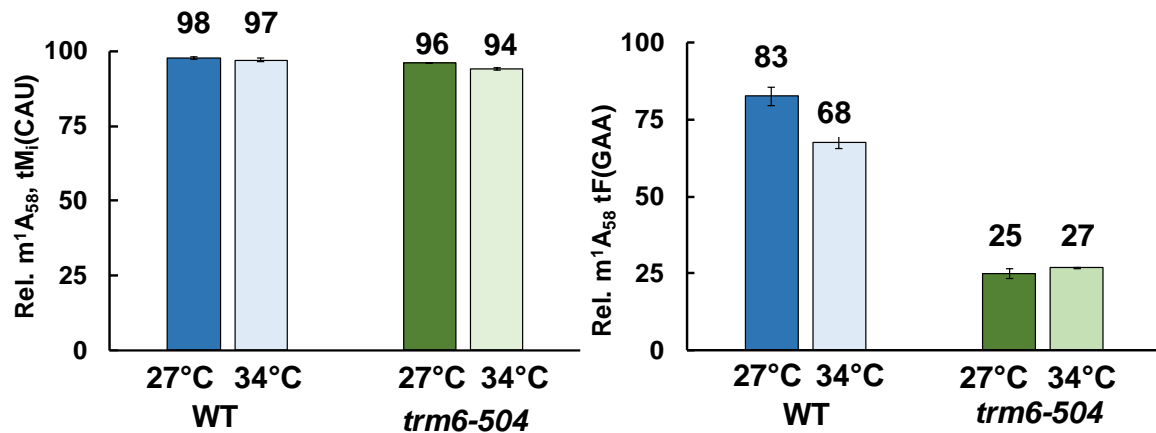

Supplement: S15 Fig — (A) Primer extension analysis of m1A58 modification in tRNAiMet(CAU) and tRNAPhe(GAA). Bulk RNA from S. cerevisiae trm6-504 mutants and WT cells grown for Fig 7B was analyzed by poison primer extension assay, as described in Materials and Methods, with the P1 primer (complementary to tRNAiMet(CAU) nt 76–61) and P2 primer (complementary to tRNAPhe(GAA) 76–60) in the presence of ddCTP, producing a stop at G57 for both tRNAiMet(CAU) and tRNAPhe(GAA), and a stop at N59 for m1A58. (B) Quantification of the poison primer extension. Values were calculated by first subtracting background levels, as in Fig 1E. (PDF) [file pgen.1010215.s015.pdf]

Fig S17

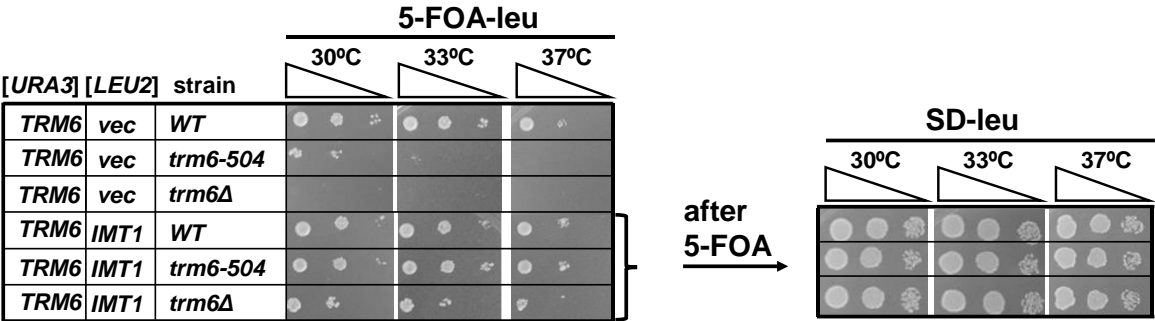

Supplement: S17 Fig — S. cerevisiae WT, trm6-504, and trm6Δ strains containing [2μ PGALTRM6 URA3] plasmid [81] and [2μ IMT1 LEU2] plasmids or empty vector, as indicated, were grown overnight in SD-leu media at 30°C and analyzed by spotting on SD-Leu media containing 5-FOA. Then cells from the 5-FOA plates were streaked for colonies, inoculated into SD-Leu media and grown overnight, and re-spotted on SD-Leu media. (PDF) [file pgen.1010215.s017.pdf]
