# Supplementary material for: Initiator tRNA lacking 1-methyladenosine is targeted by the rapid tRNA decay pathway in evolutionarily distant yeast species
Source: PLoS Genet. 2022 Jul 28;18(7):e1010215. doi: 10.1371/journal.pgen.1010215 (PMC9362929; doi:10.1371/journal.pgen.1010215)

Fig S6

A

	dhp1-6 (Y669C)	*dhp1-5* (S737P)
S. pombe	DLASIDV-KFELNQ-PFKPYEQLLGVLPAA	LLPFIDENRLLNAVSKIYPQLTEEESKRNE
D. discoideum	FFDQFEYPQYEMGE-PFKPFNQLMSVLPPA	LLPFINSIKLLKTISKTEPLLTEEEVDRNT
S. cerevisiae	GFSHLEI-KFEEGT-PFLPYEQLMSVLPAA	LLPFIDQDRLLTAVRAQYPLLSDAERARNI
A. thaliana	DLGEMDI-KFELGT-PFKPFNQLLGVFPAA	KLPFIDERRLLEAVSEVEFTLTDEEKRRNS
O. sativa	GLGQLNI-TFELGS-PFKPFDQLMGVFPAA	KLPFIDEARLLAEIKKVEHTLTPEEARRNS
O. tauri	NLAGIST-DFELGK-PFKPFSQLMGVLPAA	LLPWIDSRRLLEQTDMLEYTLTAEEKRRNS
D. melanogaster	NIQGLST-MFEKGTKPFNPLEQLMGVFPAA	LLPFVDEKRLFKALVPYYDQLTGEEVKRNK
H. sapiens	GIADMPS-DFEKGTKPFKPLEQLMGVFPAA	LLPFVDERRLRAALEEVYPDLTPEETRRNS
M. musculus	GIADMSS-EFEKGTKPFKPLEQLMGVFPAA	LLPFVDERRLRAALEEVYPDLTPEENRRNS
X. tropicalis	NISCLFK-EFEKGTKPFKPLEQLMGVFPAA	LLPFVDERRLRAALEEVYPDLTPDETRRNS
C. elegans	F--VGMRMEFELSE-PFHPFEQLLAVLPEA	LIPFIEEKRLLEAIEAKRSRLTSEENARNS
T. brucei	LPAVAACVKFDPGK-PFLPHQQLLAVLPPM	LIPFIEERTLLAAYETVQDRVTPEERKNNR

B

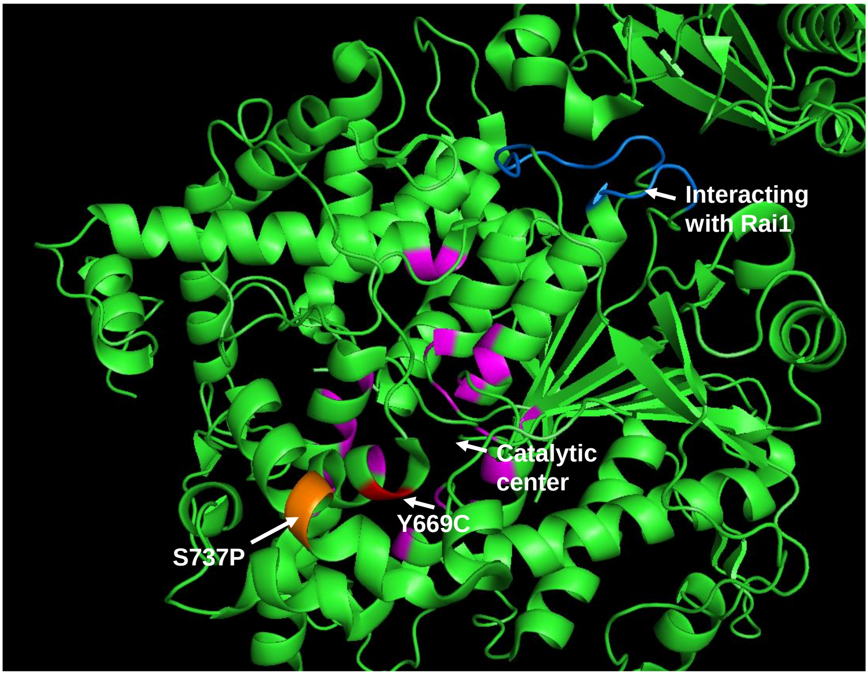

C

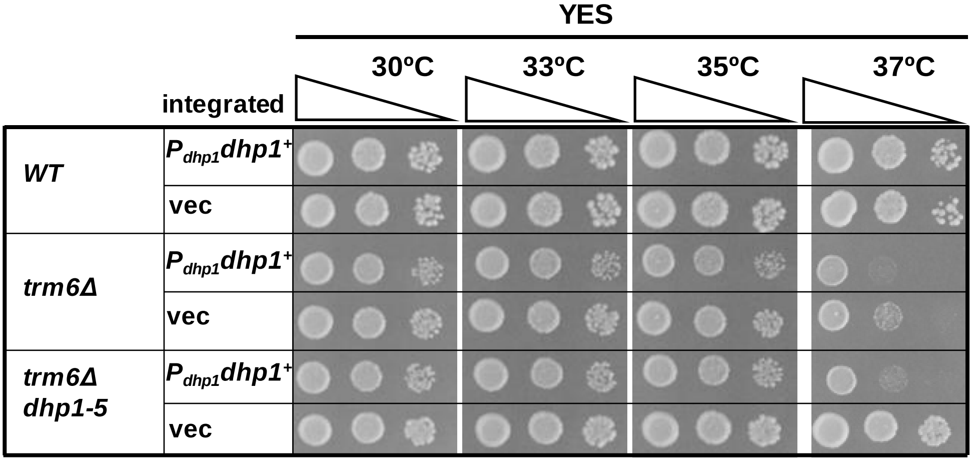

Supplement: S6 Fig — (A) Alignment of regions around the dhp1-5 (S737P) and dhp1-6 (Y669C) mutations. S. pombe Dhp1 was aligned with putative Rat1/Dhp1 orthologs from 12 evolutionarily distinct eukaryotes, using Multalin [77]; http://multalin.toulouse.inra.fr/multalin/). red, more than 80% conservation; blue 40% - 80% conservation. (B) Location of dhp1-5 (S737P) and dhp1-6 (Y669C) mutations mapped onto the S. pombe structure [78]. magenta, residues in the catalytic center; blue, residues interacting with Rai1. (C) Expression of Pdhp1 dhp1+ integrated in the chromosome restores temperature sensitive growth of the S. pombe trm6Δ dhp1-5 mutant. WT, trm6Δ, and trm6Δ dhp1-5 cells expressing a chromosomally integrated copy of Pdhp1 dhp1+ in the ura4+ locus or the control vector integrant, were grown overnight in YES media at 30°C, and analyzed for growth. (PDF) [file pgen.1010215.s006.pdf]
