# Supplementary material for: Initiator tRNA lacking 1-methyladenosine is targeted by the rapid tRNA decay pathway in evolutionarily distant yeast species
Source: PLoS Genet. 2022 Jul 28;18(7):e1010215. doi: 10.1371/journal.pgen.1010215 (PMC9362929; doi:10.1371/journal.pgen.1010215)

Fig S8

A

tol1-1
(A151D)

```
S. pombe          PIDGTKGFLRGA-QYAICLA
S. cerevisiae     PIDGTKGFLRGE-QFAVCLA
A. thaliana       PIDGTKGFLRGD-QYAVALG
O. sativa         PIDGTKGFLRGD-QYAIALA
O. tauri          PVDGTLGFVRGD-QYAIALA
D. discoideum     PIDGTLGFLRKD-QYAVALA
T. brucei         PIDGTMSFVHGSCDCCVSIG
X. tropicalis     PIDGTTNFVHRFPFVAVSIG
H. sapiens        PIDGTTNFVHRFPFVAVSIG
M. musculus       PIDGTCNFVHRFPTVAVSIG
D. melanogaster   PVDGTMNFVHAFPHSCISVG
C. elegans        PIDGTTNFVHRIPMIAICVG
```

B

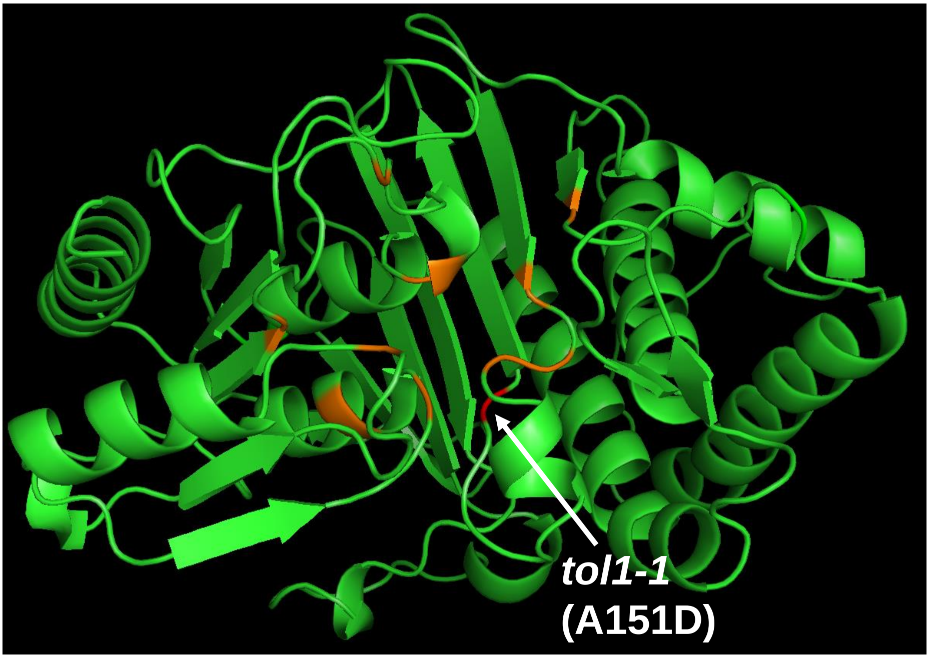

Supplement: S8 Fig — (A) Alignment of the regions around the tol1-1 (A151D) mutation. S. pombe Tol1 was aligned with putative Tol1 orthologs from 12 evolutionarily distinct eukaryotes, as in S5 Fig. red, more than 80% conservation; blue 40% - 80% conservation. (B) Location of tol1-1 (A151D) mapped onto the structure of the S. cerevisiae ortholog Met22 [79]. orange, active site residues. (PDF) [file pgen.1010215.s008.pdf]
