# Supplementary material for: Initiator tRNA lacking 1-methyladenosine is targeted by the rapid tRNA decay pathway in evolutionarily distant yeast species
Source: PLoS Genet. 2022 Jul 28;18(7):e1010215. doi: 10.1371/journal.pgen.1010215 (PMC9362929; doi:10.1371/journal.pgen.1010215)

Fig S10

A

	dhp1-7 (R177P)	dhp1-11 dhp1-14 (Y178S)	dhp1-12 (366ins) / dhp1-13 dhp1-8 (G373D)	dhp1-10 (S697C)	dhp1-9 (L743R)
S. pombe	TLAKSLR	YYIINKL	PHMGGYLTLDGSVNLARAEVILS	DENSEIIDFYPENFTIDLN	LNAVSKIYPQLTEEESKRNE
D. discoideum	RVAESLR	TYVAEKL	PSFDDYLVSNGEVNFPRLSQIFV	VEESPIIEFYPRAFRIDRG	LKTISKTEPLLTEEEVDRNT
S. cerevisiae	KLAAALR	YWTAFKL	PKLKTYMTCDGVLNLPSVETLLQ	EPDSEIIDFYPEEFPIDMN	LTAVRAQYPLLSDAERARNI
A. thaliana	VLSIALQ	YYVHLRL	RSFDGYLTDGCKPNLKRVEQFIQ	DPNSPIIDFYPTDFEVDMN	LEAVSEVEFTLTDEEKRRNS
O. sativa	VLSIALQ	YYIHLRL	PSMGGYLTDACTPDLNKVEHFIQ	DANSPIIDFYPTDFEVDMN	LAEIKKVEHTLTPEEARRNS
O. tauri	RLSEALK	YYVHDRL	PTLGGYLCADGRPNLPVVENFVQ	DKDSPIIDFYPEDFDLDMN	LEQTDMLEYTLTAEEKRRNS
D. melanogaster	RLSKCLH	YFVHDRQ	YKTRGYLTDSGDVNLDRVQLIMT	DPESPIIDFYPEDFKIDLN	FKALVPYYDQLTGEEVKRNK
H. sapiens	NLAKCLR	YYIADRL	HKTGGYLTESGYVNLQRVQMIML	DPDSSIIDFYPEDFAIDLN	RAALEEVYPDLTPEETRRNS
M. musculus	NLAKCLR	YYIADRL	HKTGGYLTESGYVNLQRVQMIML	DPDSSIIDFYPEDFAIDLN	RAALEEVYPDLTPEENRRNS
X. tropicalis	NLAKCLR	YYIADRL	HKTGGYLTDSGFVNLERVQMIML	DPDSSIIDFYPDDFAIDLN	RAALEEVYPDLTPDETRRNS
C. elegans	RLHQKLD	DWIKTKS	PTLDGYINEAGYLNLSRFEAFLA	PAKSPICDFYPANFETDLN	LEAIEAKRSRLTSEENARNS
T. brucei	RISEHFQ	YFIMMKM	LLAHTYLTDMGRIDWRAVEGWLQ	--NSPLARYFPEKLQIDRE	LAAYETVQDRVTPEERKNNR

B

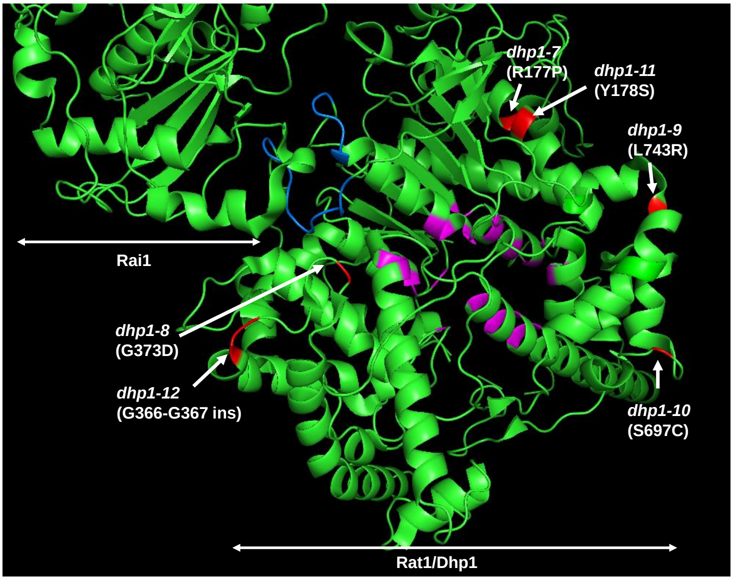

C

	tol1-2 (A297D)
S. pombe	GGSLLVEEAGGVVSD-MFGK
S. cerevisiae	AGNVIVHEAGGIHTDAMEDV
A. thaliana	AGAIVVTEAGGIVTDA-AGK
O. sativa	AGSIVVTEAGGLVTDA-SGN
O. tauri	AGVILVEEAGGTVSDA-GGA
D. discoideum	AGHIIVEEAGGIVTDF-KKQ
T. brucei	SGKIWDVCAGNLLVTEAGGV
X. tropicalis	AASVIIITEAGGVVLDA-TGG
H. sapiens	GAGIIVTEAGGVLMDV-TGG
M. musculus	AATVIIREAGGIVIDT-SGG
D. melanogaster	AGDLIVREAGGVVIDP-AGG
C. elegans	APSIIVTEAGGVVTDP-TGS

D

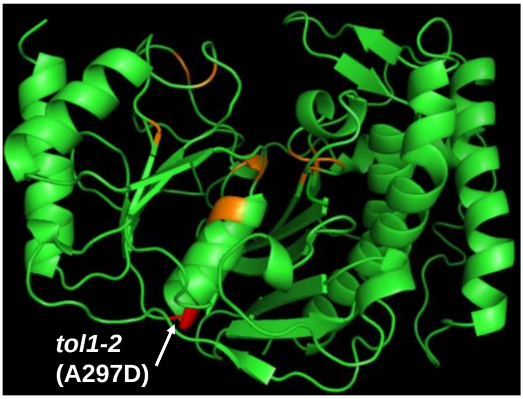

Supplement: S10 Fig — The alignment of S. pombe Dhp1 was done as in S5A Fig. (B) Location of dhp1 suppressor mutations mapped onto the S. pombe structure [78]. magenta, residues in the catalytic center; blue, residues interacting with Rai1. (C) Alignment of the regions around the tol1-2 (A297D) mutation. The alignment of S. pombe Tol1 was done as in S7 Fig. (D) Location of tol1-2 (A297D) mapped onto the structure of the S. cerevisiae ortholog Met22 [79]. orange, active site residues. (PDF) [file pgen.1010215.s010.pdf]
