# Supplementary material for: Initiator tRNA lacking 1-methyladenosine is targeted by the rapid tRNA decay pathway in evolutionarily distant yeast species
Source: PLoS Genet. 2022 Jul 28;18(7):e1010215. doi: 10.1371/journal.pgen.1010215 (PMC9362929; doi:10.1371/journal.pgen.1010215)
Supplement: S16 Fig — (A) Primer extension analysis of m1A58 modification in tRNAiMet(CAU). Bulk RNA from the growth done for Fig 7B was analyzed by poison primer extension assay with the P1 primer in the presence of ddATP, producing a stop at U55 or at A59 for m1A58. (B) Primer extension analysis of m1A58 modification in tRNAPhe(GAA). Bulk RNA from the growth done for Fig 7B was analyzed by poison primer extension assay with the P2 primer in the presence of ddCTP, producing a stop at G57 and for m1A58 at U59. (C) Quantification of the data from (A) and (B). (PDF) [file pgen.1010215.s016.pdf]

A

$tRNA_i^{Met(CAU)}$

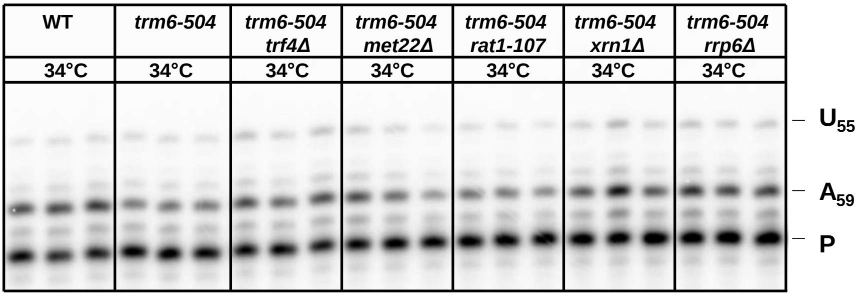

B

$tRNA^{Phe(GAA)}$

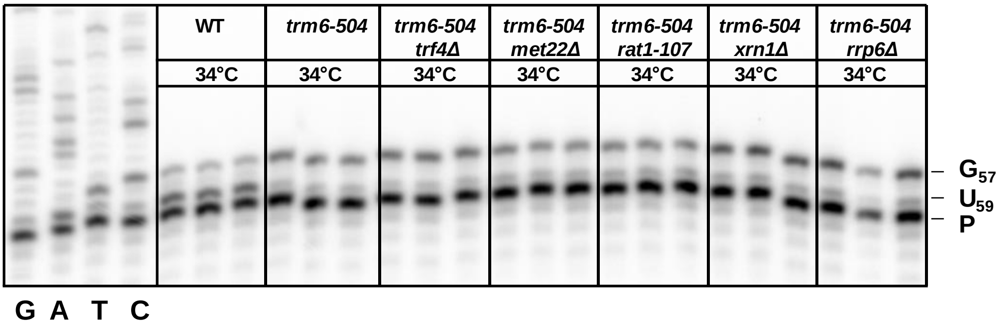

C

	avg % m^1A_{58} of total tRNA	
strain	$tRNA_i^{Met(CAU)}$	$tRNA^{Phe(GAA)}$
WT	97.6 ± 0.3	81.8 ± 0.7
trm6-504	94.8 ± 0.7	37.2 ± 1.2
trm6-504 trf4Δ	92.6 ± 0.9	29.1 ± 2.0
trm6-504 met22Δ	94.5 ± 0.9	44.0 ± 1.5
trm6-504 rat1-107	95.2 ± 0.2	44.0 ± 2.1
trm6-504 xrn1Δ	94.3 ± 1.0	30.0 ± 2.0
trm6-504 rrp6Δ	94.9 ± 0.4	28.3 ± 3.3
