# Supplementary material for: Initiator tRNA lacking 1-methyladenosine is targeted by the rapid tRNA decay pathway in evolutionarily distant yeast species
Source: PLoS Genet. 2022 Jul 28;18(7):e1010215. doi: 10.1371/journal.pgen.1010215 (PMC9362929; doi:10.1371/journal.pgen.1010215)
Supplement: S1 Table — (PDF) [file pgen.1010215.s018.pdf]

Table S1. ***S. pombe*** **strains used in this study**

Strain name	Parent strain	Genotype	Source
YMT 599		WT	[1]
YMT 621	YMT 599	*trm6Δ::kanR*	This study
YMT 654	YMT 599	*trm61Δ::kanR*	This study
YMT 652-4A	YMT 621	*trm6Δ::kanR sup9 dhp1-5* (*dhp1-S737P*)	This study
YMT 828-3A	YMT 621	*trm6Δ::kanR sup18 tol1-1 (A151D)*	This study
YMT 828-6B	YMT 621	*trm6Δ::kanR sup25 dhp1-6 (Y669C)*	This study
YMT 741	YMT 599	*ura4*$^{+}$*::dhp1*$^{+}$	This study
YMT 722	YMT 599	WT *ura4*$^{+}$	This study
YMT 742	YMT 621	*trm6Δ::kanR ura4*$^{+}$	This study
YMT 723	YMT 621	*trm6Δ::kanR ura4*$^{+}$*::dhp1*$^{+}$	This study
YMT 743	YMT 652-4A	*trm6Δ::kanR dhp1-5 ura4*$^{+}$*::dhp1*$^{+}$	This study
YMT 872	YMT 652-4A	*trm6Δ::kanR dhp1-5 ura4*$^{+}$	This study
YMT 731	YMT 599	*imt06Δ::hygR*	This study
YMT 732	YMT 621	*trm6Δ::kanR imt06Δ::hygR*	This study
YMT 807	YMT 732	*trm6Δ::kanR imt06Δ::hygR ura4*$^{+}$*::imt06*$^{+}$	This study
YMT 733-1C	YMT 732	*trm6Δ::kanR imt06Δ::hygR sup26 dhp1-9 (L743R)*	This study
YMT 733-1F	YMT 732	*trm6Δ::kanR imt06Δ::hygR sup27 dhp1-10 (S697C)*	This study
YMT 733-2A	YMT 732	*trm6Δ::kanR imt06Δ::hygR sup29 dhp1-8 (G373D)*	This study
YMT 733-2D	YMT 732	*trm6Δ::kanR imt06Δ::hygR sup30 dhp1-8 (G373D)*	This study
YMT 733-2G	YMT 732	*trm6Δ::kanR imt06Δ::hygR sup31 dhp1-12 (366 ins)*	This study
YMT 733-3A	YMT 732	*trm6Δ::kanR imt06Δ::hygR sup32 dhp1-7 (R177P)*	This study
YMT 733-3B	YMT 732	*trm6Δ::kanR imt06Δ::hygR sup33 dhp1-11 (Y178S)*	This study
YMT 733-3E	YMT 732	*trm6Δ::kanR imt06Δ::hygR sup34 dhp1-11 (Y178S)*	This study
YMT 734-1E	YMT 732	*trm6Δ::kanR imt06Δ::hygR sup36 tol1-2 (A297D)*	This study
YMT 845	YMT 621	*trm6Δ::kanR cid14Δ::hygR*	This study
YMT 846	YMT 599	*cid14Δ::hygR*	This study

References

1. De Zoysa T, Phizicky EM. Hypomodified tRNA in evolutionarily distant yeasts can trigger rapid tRNA decay to activate the general amino acid control response, but with different consequences. PLoS Genet. 2020;16(8):e1008893.
