# Supplementary material for: Initiator tRNA lacking 1-methyladenosine is targeted by the rapid tRNA decay pathway in evolutionarily distant yeast species
Source: PLoS Genet. 2022 Jul 28;18(7):e1010215. doi: 10.1371/journal.pgen.1010215 (PMC9362929; doi:10.1371/journal.pgen.1010215)
Supplement: S2 Table — (PDF) [file pgen.1010215.s019.pdf]

Table S2. *S. cerevisiae* strains used in this study

Strain name	Parent strain	Genotype	Source
BY4741		*MATa his3Δ1 leu2Δ0 met15Δ0 ura3Δ0*	Standard laboratory strain
YMT 412	BY4741	*BY4741*, $HIS3^{+}$	This study
YMT 678	YMT 412	*trm6-504*, $HIS3^{+}$	This study
YMT 681	YMT 678	*trm6-504 met22Δ::kanR*, $HIS3^{+}$	This study
YMT 702	YMT 678	*trm6-504 rat1-107::URA3*, $HIS3^{+}$	This study
YMT 706	YMT 678	*trm6-504 rrp6Δ::kanR*, $HIS3^{+}$	This study
YMT 708	YMT 678	*trm6-504 xrn1Δ::kanR*, $HIS3^{+}$	This study
YMT 736	YMT 678	*trm6-504 trf4Δ::kanR*, $HIS3^{+}$	This study
YMT 655		*MATa gcn2-101, ino1-29, ura3-52, ino1 (HIS4-lacZ, ura3-52)*	[1]
YMT 656		MATa trm6-504, gcn2-101, ino1-29, ura3-52, ino1 (HIS4-lacZ, ura3-52)	[1]
YMT 661	YMT 656	Y190 *trm6-504 met22Δ::kanR*	This study
YMT 667	BY4741	BY4741 [P_{GAL} *TRM6 URA3 2μ*]	This study
YMT 668	BY4741	*BY4741* [*URA3 2μ*]	This study
YMT 673	YMT 667	*trm6Δ::bleR* [P_{GAL} *TRM6 URA3 2μ*]	This study
YMT 754	YMT 717	*trm6Δ::bleR met22Δ::kanR trf4Δ::hygR* [P_{GAL} *TRM6 URA3 2μ*]	This study
YMT 755	YMT 673	*trm6Δ::bleR trf4Δ::hygR* [P_{GAL} *TRM6 URA3 2μ*]	This study
YMT 717	YMT 673	*trm6Δ::bleR met22Δ::kanR* [P_{GAL} TRM6 URA3 2μ]	This study
YMT 660	BY4741	*met22Δ::kanR*	This study
YMT 756	YMT 001	*trf4Δ::hygR*	This study
YMT 757	YMT 660	*met22Δ::kanR trf4Δ::hygR*	This study
YMT 716	YMT 668	*met22Δ::kanR* [*URA3 2μ*]	This study
YMT 839	YMT 660	*met22Δ::kanR* [P_{GAL} *TRM6 URA3 2μ*]	This study
YMT 763	YMT 756	*trf4Δ::hygR* [*URA3 2μ*]	This study
YMT 840	YMT 756	*trf4Δ::hygR* [P_{GAL} *TRM6 URA3 2μ*]	This study
YMT 764	YMT 757	*met22Δ::kanR trf4Δ::hygR* [*URA3 2μ*]	This study
YMT 823	YMT 754	*trm6Δ::bleR met22Δ::kanR trf4Δ::hygR*	This study
YMT 765	YMT 667	*BY4741* [P_{GAL} *TRM6 URA3 2μ*] [*LEU2 2μ*]	This study
YMT 766	YMT 678	*trm6-504* [P_{GAL} *TRM6 URA3 2μ] [LEU2 2μ]*, $HIS3^{+}$	This study
YMT 767	YMT 673	*trm6Δ::bleR* [P_{GAL} *TRM6 URA3 2μ*] [*LEU2 2μ*]	This study
YMT 770	YMT 667	BY4741 [P_{GAL} *TRM6 URA3 2μ*] [*IMT1 LEU2 2μ*]	This study
YMT 771	YMT 678	*trm6-504* [P_{GAL} *TRM6 URA3 2μ*] [*IMT1 LEU2 2μ*], $HIS3^{+}$	This study
YMT 772	YMT 673	*trm6Δ::bleR* [P_{GAL} *TRM6 URA3 2μ*] [*IMT1 LEU2 2μ*]	This study

References

1. Kadaba S, Krueger A, Trice T, Krecic AM, Hinnebusch AG, Anderson J. Nuclear surveillance and degradation of hypomodified initiator tRNAMet in S. cerevisiae. Genes Dev. 2004;18(11):1227-40.
