# Supplementary material for: Initiator tRNA lacking 1-methyladenosine is targeted by the rapid tRNA decay pathway in evolutionarily distant yeast species
Source: PLoS Genet. 2022 Jul 28;18(7):e1010215. doi: 10.1371/journal.pgen.1010215 (PMC9362929; doi:10.1371/journal.pgen.1010215)
Supplement: S3 Table — (PDF) [file pgen.1010215.s020.pdf]

Table S3 Plasmids used in this study

Plasmid	Parent	Description	Source
pREP3x		*leu2*$^{+}$ P$_{nmt1}$	[1]
AB 553-1	pREP3x	*leu2*$^{+}$ with a NotI between PstI and P$_{nmt1}$	[2]
EMT 256-1	pREP3x	*leu2*$^{+}$ P$_{trm6}$ *trm6*$^{+}$	This study
EMT 257-2	pREP3x	*leu2*$^{+}$ P$_{trm61}$ *trm61*$^{+}$	This study
EMT 265-1	AB 553-1	*leu2*$^{+}$ *imt06*$^{+}$	This study
EMT 264-1	AB 553-1	*leu2*$^{+}$ *sup9*$^{+}$-*imt07*$^{+}$	This study
EMT 327-1	AB 553-1	*leu2*$^{+}$ P$_{tol1}$ *tol1*$^{+}$	This study
EMT 292		*ura4*$^{+}$ single integrating vector (SIV)	[3]
EMT 301-3	EMT 292	*ura4*$^{+}$::*dhp1*$^{+}$	This study
EMT 304-1	EMT 292	*ura4*$^{+}$::*imt06*$^{+}$	This study
EMT 324		*URA3* P$_{GAL}$ *TRM6* 2µ	[4]
EMP 881		*URA3* 2µ	Laboratory strain
EMT 306-1		*LEU2* 2µ *IMT1*	this study
EMP 824		*LEU2* 2µ	Laboratory strain
EMT 386		*leu2*$^{+}$ P$_{cid14}$ *cid14*$^{+}$	this study

References

1. Forsburg SL. Comparison of Schizosaccharomyces pombe expression systems. Nucleic Acids Res. 1993;21(12):2955-6.
2. De Zoysa T, Phizicky EM. Hypomodified tRNA in evolutionarily distant yeasts can trigger rapid tRNA decay to activate the general amino acid control response, but with different consequences. PLoS Genet. 2020;16(8):e1008893.
3. Vjestica A, Marek M, Nkosi PJ, Merlini L, Liu G, Berard M, et al. A toolbox of stable integration vectors in the fission yeast Schizosaccharomyces pombe. J Cell Sci. 2020;133(1).
4. Gelperin DM, White MA, Wilkinson ML, Kon Y, Kung LA, Wise KJ, et al. Biochemical and genetic analysis of the yeast proteome with a movable ORF collection. Genes Dev. 2005;19(23):2816-26.
