# Supplementary material for: Initiator tRNA lacking 1-methyladenosine is targeted by the rapid tRNA decay pathway in evolutionarily distant yeast species
Source: PLoS Genet. 2022 Jul 28;18(7):e1010215. doi: 10.1371/journal.pgen.1010215 (PMC9362929; doi:10.1371/journal.pgen.1010215)
Supplement: S4 Table — (PDF) [file pgen.1010215.s021.pdf]

Table S4. Oligomers used for northern analysis

Name	Target tRNA	Probe 5'-3'	Sequence
OMT 530	tF(GAA)	58-37	TCGAACCGATGACCAACAGATC
OMT 602	tMi(CAU)	57-38	CGATCCTGGGACCTACGGGT
OMT 623	tA(AGC)	57-39	CGAATCTGGGACCTCTTCC
OMT 624	tT(AGU)	19-1	ACCACTGAGCTACAAGAGC
OMT 625	tG(GCC)	76-59	TGGTGCTTTGGCCGGGAA
OMT 626	tY(GUA)	57-39	CGAACTAGCGACCAACCGG
TDZ 123	tMe(CAU)	20-1	ACCGACTGAGCTACAGAAGC
TDZ 177	tP(AGG)	27-5	GGTATCATACCACTAGACCAAAC
TDZ 184	tC(GCA)	22-1	CTAACCACTGAGCTATGACCCC
TDZ 185	tW(CCA)	22-1	CTACCAACTGAGTTAAGGGGCC
OMT 384	*tF(GAA)*	*76-59*	*TGGTGCGAATTCTGTGGA*
OMT 596	*tMi(CAU)*	*57-38*	*CGATCCGAGGACATCAGGGT*
OMT 603	*tA(UGC)*	*73-53*	*TGGACGCAACCGGAATCGAAC*
OMT 604	*tL(CAA)*	*62-e3*	*AGATTCGAACTCTTGCATCTT*
OMT 617	*tMe(CAU)*	*56-38*	*GAACTCTCGACCTTCAGAT*

[a]Note that *S. cerevisiae* oligomers are indicated in italics
