# Supplementary material for: Initiator tRNA lacking 1-methyladenosine is targeted by the rapid tRNA decay pathway in evolutionarily distant yeast species
Source: PLoS Genet. 2022 Jul 28;18(7):e1010215. doi: 10.1371/journal.pgen.1010215 (PMC9362929; doi:10.1371/journal.pgen.1010215)
Supplement: S5 Table — (PDF) [file pgen.1010215.s022.pdf]

Table S5. Oligomers used for primer extension analysis

Name	Target tRNA	Probe 5'-3'	Sequence
OMT 775	tMi(CAU)	76-61	TGGTTGCGCGGCCAGG
OMT 477	tY(GUA)	76-61	TGGTCTCCTGAGCCAG
OMT 630	*tMi(CAU)*	*76-61*	*TGGTAGCGCCGCTCGG*
OMT 631	*tF(GAA)*	*76-60*	*TGGTGCGAATTCTGTGG*

[a]Note that *S. cerevisiae* oligomers are indicated in italics
