# Supplementary material for: Initiator tRNA lacking 1-methyladenosine is targeted by the rapid tRNA decay pathway in evolutionarily distant yeast species
Source: PLoS Genet. 2022 Jul 28;18(7):e1010215. doi: 10.1371/journal.pgen.1010215 (PMC9362929; doi:10.1371/journal.pgen.1010215)
Supplement: Attachment — Submitted filename: response to reviewers 06-20-22b.docx [file pgen.1010215.s023.docx]

**Response to reviewers**

**"Initiator tRNA lacking 1-methyladenosine is unexpectedly targeted by the rapid tRNA decay pathway in evolutionarily distant yeast species"**

**Reviewer #1:**

**Comment 1:** line 280-281: shouldn’t the trm6Δ imt06Δ mutant with the integrated imt06+ gene show poor growth at 39C compared to WT cells (the phenotype of trm6∆?)

**Response:** We have changed the text as shown below to indicate more unambiguously that the *S. pombe* *trm6Δ imt06Δ* mutant growth defect is fully suppressed when tRNA_i_^Met(CAU)^ is expressed simultaneously from two sources: an integrated copy and a plasmid.

"Moreover, the *trm6Δ imt06Δ* growth defect was strictly due to the loss of tRNA_i_^Met(CAU)^ because *trm6Δ imt06Δ* strains expressing both an integrated copy of *imt06^+^* and a [*leu2^+^* *imt06^+^*] plasmid grew as well as WT on YES media at all temperatures up to 39°C (S9B Fig)."

**Comment 2:** lines 417-419: The claim that decay of tRNAiMet(CAU) in S. pombe trm6Δ mutants does not involve the nuclear surveillance pathway is not fully justified by the results in Fig. S11. They need to measure tRNAiMet(CAU) levels in the trm6∆ and trm6∆cid14∆ mutants examined for growth in Fig. S11 and show no detectable increase in tRNA level in the trm6∆cid14∆ double mutant vs trm6∆ mutant, including a trm6∆dhp1-7 double mutant as a positive control. Also, can they verify the successful deletion of CID14 in their double mutant by showing a growth or biochemical phenotype of cid14∆ that can be complemented by WT CID14?

**Response:** The reviewer makes a good point. We now show additional experiments documenting that the both *trm6Δ cid14Δ* and *cid14Δ* mutants are sensitive to 5-fluorouracil (5-FU) (Fig S12A), and that this phenotype is complemented by expression of a [*CID14*] plasmid (Fig S12B).

We also show an additional experiment (Fig. S13), showing that a *cid14Δ* mutation has almost no effect on restoring tRNA_i_^Met(CAU)^ levels in a *trm6Δ* mutant at high temperature, as tRNA_i_^Met(CAU)^ levels are only very marginally increased in a *trm6Δ cid14Δ* mutant relative to a *trm6Δ* mutant (21% vs 18%) compared to 39% for a *trm6Δ dhp1-5* mutant.

The accompanying text for both of these experiments is shown below:

"Further examination suggests the lack of participation of the Trf4 ortholog Cid14 of the nuclear surveillance pathway (48-50) in quality control of tRNA_i_^Met(CAU)^ in *S. pombe trm6Δ* mutants. A *cid14Δ* mutation was introduced into WT and *trm6Δ* strains and independent isolates were confirmed by PCR (Materials and Methods), after which the resulting strains were shown to be sensitive to 5-fluorouracil (5-FU) (S12 Fig A, B), as previously reported (48, 51). We observed little, if any, suppression of the *trm6Δ* growth defect in the *trm6Δ* *cid14Δ* strains (S13 Fig A), and only very minor restoration of tRNA_i_^Met(CAU)^ levels at high temperature, relative to levels in *trm6Δ* mutants (21% vs 18%, compared to 39% in the *trm6Δ dhp1-5* strain) (S13 Fig B, C). Thus, we infer that tRNA_i_^Met(CAU)^ is degraded in *S. pombe trm6Δ* and *trm6Δ imt06Δ* mutants primarily by the RTD pathway, and not appreciably by the TRAMP complex of the nuclear surveillance pathway."

**Comment 3:** lines 303-306: no mutations in the MET22 ortholog were recovered. Doesn’t this argue against saturation ot trm6∆ suppressors in S. pombe? Does deletion of the MET22 ortholog suppress the trm6∆ growth defect in S. pombe?

**Response:** We have re-written the passage as shown below to further emphasize that *tol1* is the *MET22* ortholog, and that *tol1* is essential in *S. pombe*.

"These findings underscore the crucial role of Dhp1 and Tol1, and thus of the RTD pathway, in quality control of tRNA_i_^Met(CAU)^ in *S. pombe trm6Δ* mutants. Indeed, the isolation of eight genetically distinct *dhp1/rat1* alleles (two in *trm6Δ* mutants and six in *trm6Δ imt06Δ* mutants) and two distinct *tol1/met22* alleles (one each in the *trm6Δ* mutant and the *trm6Δ imt06Δ* mutant) argues that the genetic landscape of *trm6Δ* suppressors has been nearly saturated, particularly considering that both *dhp1* and *tol1* are essential in *S. pombe*."

**Comment 4:** the finding in Fig. S13A-B, that A58 of tRNAiMet(CAU) is nearly fully modified at both 27°C and 34°C in trm6-504 mutants is initially surprising, considering the genetic and biochemical evidence for reduced stability of tRNAiMet(CAU) in this strain, shown here and previously.
They state that “The nearly complete modification of tRNAiMet(CAU) in all of the trm6-504 strains with mutations in the nuclear surveillance or the RTD pathway argues for competition between the Trm6:Trm61 enzyme and the decay pathways.” It would be helpful to elaborate on this conclusion by reminding the reader in this context that the levels of tRNAiMet(CAU) are greatly reduced by trm6-504, to <20% of WT levels, and that the remaining ~20% is fully modified. This would be expected if trm6-504 strongly impairs, but does not abolish, m1A modification of tRNAiMet(CAU) and the unmodified species are essentially eliminated from the cells by degradation. Otherwise, there is an impression from the data in Fig. S13A-B that trm6-504 has no impact on tRNAiMet(CAU) methylation.

**Response:** We agree with the reviewer's point that we needed to remind the reader of the tRNA_i_^Met(CAU)^ decay in trm6-504 mutants, and that our results refer to the undegraded tRNA_i_^Met(CAU)^. We re-wrote two passages as shown below to emphasize that the *trm6-504* mutants have reduced levels of tRNA_i_^Met(CAU)^, although it is all modified with m^1^A_58_:

"By using poison primer extension to measure m^1^A_58_ modification, we found that A_58_ of tRNA_i_^Met(CAU)^ was nearly fully modified at both 27°C and 34°C in both *trm6-504* mutants (96% and 94%) and WT strains (98% and 97%) (S15 Fig), although tRNA_i_^Met(CAU)^ levels are reduced in *trm6-504* mutants. "

"Thus, although tRNA_i_^Met(CAU)^ decay at 34°C is inhibited in *trm6-504* strains with mutations in the nuclear surveillance or the RTD pathway, the nearly complete modification of the remaining, undegraded tRNA_i_^Met(CAU)^ in all of these *trm6-504* derivative strains argues for competition between the Trm6:Trm61 enzyme and the decay pathways."

**Comment 5:** There are unexplained data in Fig. 8C-D involving deletions of IMT1, 2, 3 genes. Also, the data for the trm6-504 mutant in this experiment were not mentioned in the text.

**Response:** We have modified the text to explain the data for the imt1Δ, imt2Δ, and imt3Δ strains, as well as for the *trm6-504* strain, as shown below:

"As tRNA_i_^Met(CAU)^ levels in *trm6Δ met22Δ trf4Δ* strains were only 48% of WT levels, comparable to the tRNA_i_^Met(CAU)^ levels in *trm6-504* mutants and somewhat less than tRNA_i_^Met(CAU)^ levels in mutants lacking one of the four *IMT* genes (Fig 8C and 8D), we infer that tRNA_i_^Met(CAU)^ lacking m^1^A_58_ modification is still being degraded in this strain."

**Other comments:**

**Comment 6** -p. 9: Is the failure of high-copy imt07+ to suppress the trm6/61 mutants due to a lack of overexpression of tRNAiMet(CAU, as judged by Northern?

**Response:** We have included an additional experiment, to explicitly show that strains with an [*imt06*] plasmid overexpress tRNA_i_^Met(CAU)^ to higher levels than strains with an [*imt07*] plasmid (Fig. S4). The accompanying new text is shown within the passage below:

"We found that the temperature sensitive growth defect of *S. pombe* *trm6Δ* mutants on EMMC-leu media was completely suppressed by expression of the stand-alone *imt06^+^* gene, growing identically to that of an *S. pombe* *trm6Δ* [P*_trm6_ trm6^+^*] strain at high temperature (Fig 2C), whereas expression of *imt07^+^* from the tRNA^Ser^-tRNA_i_^Met^ tandem gene only modestly suppressed the *trm6Δ* temperature sensitivity. Consistent with this result, northern analysis showed that levels of tRNA_i_^Met(CAU)^ are much higher in strains overexpressing *imt06^+^* than in strains overexpressing *imt07^+^* (S4 Fig)."

**Comment 7** -the methods used for isolating the spontaneous suppressors of trm6∆ should be described.

**Response:** This is now included in Materials and Methods, as shown below:

"Spontaneous suppressors of *S. pombe trm6Δ* and *trm6Δ imt06Δ* mutants were isolated by growing cultures of individual colonies in YES media at 30°C, followed by plating 10^7^ cells on YES and EMMC plates at 39°C (for *trm6Δ* mutants) and on YES plates at 37°C (for *trm6Δ imt06Δ* mutants)."

**Comment 8** -Fig. 5B: typo: met06∆ should be imt06∆

**Response:** done -- thanks for noticing this

**Reviewer #2:**

**Specific comments:**

**Comment 1:** I wonder if it would be good to mention to readers the original names for TRM6 (GCD10) and TRM61 (GCD14) in yeast. Perhaps the field has fully adapted to the TRM nomenclature; however, some readers might still search for papers using the old GCD names.

**Response:** good point -- we added this information in the abstract and in the introduction

**Comment 2:** Lines 270-1: editorial issue, should “(13, 22) (24, 46)” be combined to a single citation for all four papers?

**Response:** done

**Comment 3:** Line 309: is there a positive control that could be used to confirm the cid14-deletion mutation? While PCR assays were used to confirm the mutations, it would be reassuring to see an expected phenotypic effect of the cid14-deletion. However, I am not aware if such a test exists.

**Response:** As described more fully above in our response to Comment 2 of Reviewer 1, we have now included an additional figure (Fig S12 A and S12B) showing that our *cid14Δ* and *trm6Δ cid14Δ* mutants were 5-FU sensitive, as previously reported in the original characterization of the *cid14Δ* mutant strain, and later shown in a high throughput analysis.

**Comment 4:** Lines 347-348: At this point in the paper, is it possible that the RTD mutants might be indirectly affecting tRNA(iMet) levels through the nuclear surveillance pathway? While later data rule this out, it seems possible that RTD mutants could impair the nuclear surveillance pathway (perhaps impair expression of an enzyme in the pathway) and thereby affect tRNA(iMet) levels. Might want to word soften the conclusion here.

**Response:** good point. We softened the text as shown below:

"A parsimonious interpretation of these results is that all components of the RTD pathway are involved in tRNA_i_^Met(CAU)^ quality control in *trm6-504* mutants, and that the nuclear surveillance pathway and RTD pathway each contribute substantially to this tRNA_i_^Met(CAU)^ quality control."

**Comment 5:** Lines 359-360: based on the observation that all tRNA(iMet) has the m1A58 modification, the authors posit that tRNA(iMet) is a preferred substrate of Trm6:Trm61. However, an alternative hypothesis is that tRNA(iMet) lacking the m1A58 modification is turned over more rapidly than other tRNAs lacking this modification. Thus, it is not more rapid modification, but more rapid turnover of unmodified tRNA that is responsible for high levels of modified tRNA(iMet) in the mutant cells.

**Response:** We had accounted for the more rapid turnover of tRNA_i_^Met(CAU)^ in the sentence, but we have now re-worded it to clarify our reasoning, as shown below:

"As tRNA_i_^Met(CAU)^ is 96% modified at low temperature and present at 39% of WT levels, whereas tRNA^Phe(GAA)^ is 25% modified and present at 97% of WT levels, these findings suggest that tRNA_i_^Met(CAU)^ is the preferred substrate of Trm6:Trm61. "

**Comment 6:** Lines 386-387: while the results in the trm6-deletion mutant demonstrate that both the RTD and nuclear surveillance pathways contribute to turnover of hypomodified tRNA(iMet), I am wondering if the two pathways are fully separate or if there might be some overlap. In Figure 7C it is shown that tRNA(iMet) levels are at 0.16 in the trm6-504 mutant and increase to 0.32 in the met22 mutant, 0.67 in the xrn1 mutant, and 0.52 in the trf4 mutant. I am wondering what happens in the trm6-504 background when both pathways are inactivated (met22 trf4 or xrn1 trf4 double mutants). Are the effects of the mutants additive or perhaps less than additive because the two pathways, even though physically separated, can sometimes act on the same tRNAs?

**Response:** While we appreciate the suggestion and the reasoning of the reviewer, we have not attempted this proposed experiment because it is fraught with difficulties in measurement and interpretation. This experiment is essentially calling for a quantitative epistasis analysis of tRNA_i_^Met(CAU)^ levels at high temperature in a triple mutant strain having a conditional *trm6-504* mutation and also lacking two major decay pathways. While our data generally has low error bars and is fairly rigorous, we do not feel like we could properly distinguish additive effects from partially additive effects, as would be predicted if the two pathways influence one another to some extent. This is particularly a problem because adding two measured tRNA_i_^Met(CAU)^ levels (to compare to the triple mutant) requires a propagated error. There is simply not enough accuracy in our measurements to obtain a meaningful result, without at least several additional supporting experiments to be able to make a claim that the two decay pathways might influence one another. Thus, although an intriguing idea, this topic is better left for a much more complete analysis.

**Reviewer #3:**

**Comment 1:** Line 455 – the authors should include a figure for their model.

**Response:** The reviewer is correctly suggesting that the model needed clarification, so we have re-written the passage to clarify our description of the model, as shown below:

"Our finding that tRNA^Phe(GAA)^ in *S. cerevisiae trm6-504* mutants had very similar m^1^A_58_ modification at both 27°C and 34°C suggests that Trm6:Trm61 is not temperature sensitive. If so, the reduced tRNA_i_^Met(CAU)^ levels at high temperature in *trm6-504* mutants would imply that tRNA_i_^Met(CAU)^ lacking m^1^A_58_ is itself temperature sensitive, perhaps becoming partially unfolded at high temperature. One could envision a model in which tRNA_i_^Met(CAU)^ lacking m^1^A_58_ is functioning in the cell cytoplasm (consistent with the viability of *S. pombe trm6Δ* mutants and of *S. cerevisiae trm6Δ trf4Δ met22Δ* mutants), but is in equilibrium with a state in which the tertiary structure is partially or completely unfolded due to lack of the modification (21, 26). As tertiary structure unfolding precedes unfolding of the individual helices of tRNA (67), the disrupted tertiary structure due to lack of m^1^A_58_ could lead to unfolding of the acceptor stem and increased availability of the 5' end to the RTD pathway. This model is very similar to that we proposed previously to explain the increased Xrn1 susceptibility of *S. cerevisiae* tRNA^Ser(CGA)^ lacking ac^4^C_12_ and Um_44_ and for tRNA^Val(AAC)^ lacking m^7^G_46_ and m^5^C_49_ (68), and could be tested in subsequent experiments."

**Comment 2:** Line 546 - Details on how whole genome sequencing was done at UofR and should be included in the Materials and methods.

**Response:** This is now included in Materials and Methods, as shown below:

"Whole genome sequencing was performed by the University of Rochester Genomics Center at 25-50 fold coverage of the genome, and reads were compared to the corresponding parent strain, and to the reference genome."

**Comment 3:** As m1A is regulated by Alkbh in humans and these are not found in budding yeast (not sure about fission yeast), the authors could speculate on these differences. Is that a potential rational for why S pombe do not use the nuclear surveillance pathway in decay of tRNAiMet(CAU)?

**Response:** Although this is an intriguing suggestion, this speculation is beyond the scope of our study.

Minor comments.

**Comment 4:** I suggest removing “unexpected” from the title.

**Response:** We removed the word "unexpected" from the title.

**Comment 5:** I suggest the authors omit or refine this sentence from the abstract “In contrast studies have shown that S. cerevisiae trm6 mutants with reduced 1-methyladenosine (m1A58) specifically target pre-tRNAiMet(CAU) to the nuclear surveillance pathway for 3’-5’ exonucleolytic decay 35 by the TRAMP complex and nuclear exosome. “ Keep it simple in the abstract and address the re-examined findings in the introduction.

**Response:** We disagree with this suggestion, as this sentence in the abstract accurately describes the known involvement of the nuclear surveillance pathway in decay of tRNA_i_^Met(CAU)^ lacking m^1^A_58_ in *S. cerevisiae*, and sets the stage for the novelty of our findings.

**Comment 6:** In materials and methods please include city, state for all company names and catalog numbers for specific products.

**Response:** done

**Comment 7:** Line 164: move “fig” to before the numbers. Similar for lines 264, 266, 293 and 297. Check throughout.

**Response:** this format is the accepted style for *PLOS Genetics*

**Comment 8:** Line 168 – indicate how the tRNA modification was examined. HPLC

**Response:** done, as follows:

"Examination by HPLC of the nucleoside composition of purified tRNA^Tyr(GUA)^ from *trm6Δ* and *trm61Δ* mutants revealed that…"

**Comment 9:** Line 180 – delete “all” as there may be some very minor residual m1A (0.03%)

**Response:** We indicate that m1A levels are less than 0.03 % of cytidine levels because we simply cannot measure values less than this. To clarify the text, we have changed the sentence as follows:

"These results show that *S. pombe trm6^+^* and *trm61^+^* are required for all detectable m^1^A_58_ modification of cytoplasmic tRNAs."

**Comment 10:** Line 209 – Define for clarity – stand-alone tRNA met gene? - SPBTRNAMET.06 (imt06+) gene

**Response:** We clarified the passage as shown below:

"In *S. pombe*, only one of the four genes encoding tRNA_i_^Met(CAU)^ is expressed in the usual manner, as a stand-alone tRNA gene. By contrast, the other three tRNA_i_^Met(CAU)^ genes are expressed in tandem with a tRNA^Ser^ species as a tRNA^Ser^-tRNA_i_^Met^ dimeric transcript, which is then processed into single tRNAs {Mao, 1980 #5777}, similar to the tRNA^Arg^-tRNA^Asp^ tandem genes in *S. cerevisiae* {Seufert, 1990 #5754;Schmidt, 1980 #216}."
